# A growth factor–expressing macrophage subpopulation orchestrates regenerative inflammation via GDF-15

**DOI:** 10.1084/jem.20210420

**Published:** 2021-11-30

**Authors:** Andreas Patsalos, Laszlo Halasz, Miguel A. Medina-Serpas, Wilhelm K. Berger, Bence Daniel, Petros Tzerpos, Máté Kiss, Gergely Nagy, Cornelius Fischer, Zoltan Simandi, Tamas Varga, Laszlo Nagy

**Affiliations:** 1 Departments of Medicine and Biological Chemistry, Johns Hopkins University School of Medicine, Institute for Fundamental Biomedical Research, Johns Hopkins All Children’s Hospital, St. Petersburg, FL; 2 Department of Biochemistry and Molecular Biology, Faculty of Medicine, University of Debrecen, Debrecen, Hungary; 3 Max Delbrück Center for Molecular Medicine, Berlin, Germany; 4 Sanford Burnham Prebys Medical Discovery Institute, Orlando, FL

## Abstract

Muscle regeneration is the result of the concerted action of multiple cell types driven by the temporarily controlled phenotype switches of infiltrating monocyte–derived macrophages. Pro-inflammatory macrophages transition into a phenotype that drives tissue repair through the production of effectors such as growth factors. This orchestrated sequence of regenerative inflammatory events, which we termed regeneration-promoting program (RPP), is essential for proper repair. However, it is not well understood how specialized repair-macrophage identity develops in the RPP at the transcriptional level and how induced macrophage–derived factors coordinate tissue repair. Gene expression kinetics–based clustering of blood circulating Ly6C^high^, infiltrating inflammatory Ly6C^high^, and reparative Ly6C^low^ macrophages, isolated from injured muscle, identified the TGF-β superfamily member, GDF-15, as a component of the RPP. Myeloid GDF-15 is required for proper muscle regeneration following acute sterile injury, as revealed by gain- and loss-of-function studies. Mechanistically, GDF-15 acts both on proliferating myoblasts and on muscle-infiltrating myeloid cells. Epigenomic analyses of upstream regulators of *Gdf15* expression identified that it is under the control of nuclear receptors RXR/PPARγ. Finally, immune single-cell RNA-seq profiling revealed that *Gdf15* is coexpressed with other known muscle regeneration–associated growth factors, and their expression is limited to a unique subpopulation of repair-type macrophages (growth factor–expressing macrophages [GFEMs]).

## Teaser

Regenerative inflammation in skeletal muscle drives macrophage specification via a regeneration-promoting program. A growth factor–expressing macrophage population develops, producing GDF-15 under the control of RXR and PPARγ. GDF-15 acts as an autocrine and paracrine factor coordinating myoblast proliferation and myeloid cell invasion and activity.

## Graphical Abstract

**Figure:**
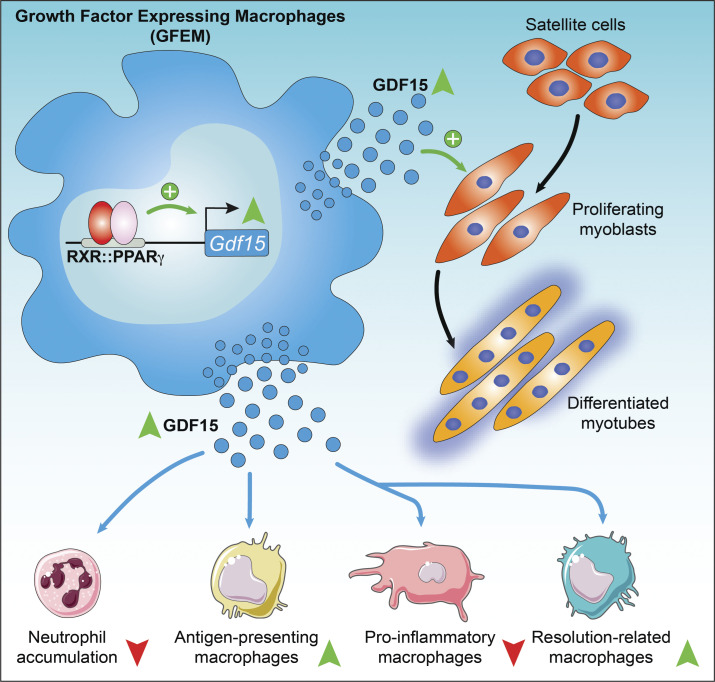

## Introduction

Tissues frequently undergo acute damage during an organism’s lifetime. To maintain the body’s integrity and homeostasis, it is critically important to achieve complete regeneration. In highly regenerative tissues such as skeletal muscle, a straightforward sensory-effectors paradigm is applied whereby organ injury induces changes detectable by distinct cell types. These changes lead to activation of effector mechanisms promoting expansion and differentiation of a quiescent population of tissue-specific stem cell–like progenitors. Strikingly, the immune system appears to have key roles in this process both as a sensor and as an effector (Arnold et al., 2007; Yona et al., 2013; Chazaud, 2014; Okabe and Medzhitov, 2014; Wang et al., 2014), which amounts to regenerative immune response. Dysregulated injury-induced immune response has been shown to impair regeneration in several tissues such as the liver, central nervous system, or skeletal muscle (Rapalino et al., 1998; Duffield et al., 2005; Laflamme and Murry, 2011; Chazaud, 2014). Importantly, immune cells, and in particular, monocyte-derived macrophages (MFs), have a dual role during damage and regeneration (Tidball, 2017; Chazaud, 2020). First, these cells sense and react to the injury, remove necrotic debris, and then transition to initiate restoration of tissue integrity as effectors via promoting resolution of inflammation and repair mechanisms acting on both the infiltrating immune cell population and the regenerating stem cell pool. The widely accepted paradigm about the two main MF populations posits that the initially appearing lymphocyte antigen 6 complex (Ly6C)^high^ MFs are inflammatory, while Ly6C^low^ MFs are repairing in cellular character (Varga et al., 2013; Varga et al., 2016a). During the regeneration phase, Ly6C^low^ repair MFs secrete cytokines and growth factors such as insulin-like growth factor 1 (IGF-1), GDF-3, IL-10, and TGF-β that act in a paracrine and/or autocrine manner and can contribute to the repair cell milieu (Fadok et al., 1998; Lu et al., 2011; Deng et al., 2012; Tonkin et al., 2015; Varga et al., 2016b). It is assumed that during this latter phase, the regenerative immune response regulates the activation of tissue progenitor cell populations to support cellular growth and differentiation. It is also likely that the microenvironment and reciprocal inter-cellular interactions mediated by local autocrine and paracrine mechanisms are driving the inflammatory-to-repair phenotypic switch (Patsalos et al., 2017). Our understanding is still incomplete on how MFs change their phenotype, employ sensory and regulatory mechanisms, and use effector functions to serve such complex reparatory roles. This is particularly important because the proper signaling between the participating cell types ensures the precisely timed progression of repair while avoiding asynchrony, which can lead to delay, fibrosis, and chronic inflammation (Tidball and Villalta, 2010; Dadgar et al., 2014). We sought to identify novel integrated sensory, regulatory, and effector mechanisms and transcriptional programs equipping the relevant MF subpopulations with the capacity to contribute to the timed progression of repair.

Here, we used the cardiotoxin (CTX)-induced skeletal muscle injury model, which is a highly reproducible in vivo model of sterile physiological inflammation (Hardy et al., 2016), to carry out an unbiased transcriptomic analysis of the circulating monocytes and the derived dynamically changing infiltrating MF subpopulations involved in regeneration. This integrated time course–based profiling revealed several transient, and remarkably, some sustained transcriptional programs during the monocyte to inflammatory and then to repair the MF continuum of cellular phenotypes. We identified growth/differentiation factor-15 (GDF-15; Bootcov et al., 1997; Lawton et al., 1997), a secreted growth factor, and a divergent member of the TGF-β superfamily. GDF-15 is being induced and then steadily and continuously up-regulated, reaching its highest level of expression in the repair MF populations within injured muscles. Importantly, mice with a hematopoietic deletion of *Gdf15* showed a pronounced delay in skeletal muscle regeneration and delayed the inflammatory to repair subtype conversion of MFs. In addition, we found that peroxisome proliferator–activated receptor γ (PPARγ) and retinoid X receptor α (RXRα) regulated the expression of *Gdf15* at the transcriptional level in repair MFs. Myeloid RXR deficiency impaired muscle regeneration, and recombinant GDF-15 could enhance the proliferation of primary myogenic precursor cells in in vitro cultures and increase the expression of antigen-presenting molecules in repair MFs in vivo*.* In summary, our data reveal a novel integrated pathway in repair MFs with sensory, gene regulatory, and effector components that includes the RXR–PPARγ–GDF-15 regulatory axis that ensures the timely onset and progression of regenerative inflammation during skeletal muscle regeneration. This finding was further corroborated and refined by single-cell RNA sequencing (scRNA-seq) data revealing a novel and functionally distinct growth factor–expressing MF (GFEM) subtype within the regenerating cell milieu, marked by growth factors GDF-15, IGF-1, and GDF-3. These data identify the cellular source and support a role for GDF-15 as a local, autocrine, and paracrine signal that participates in sustained transcriptional regeneration-promoting programs (RPPs) in repair MFs during tissue injury.

## Results

### The dynamically changing transcriptional landscape during in situ monocyte to inflammatory and repair MF transition

**Figure 1.**
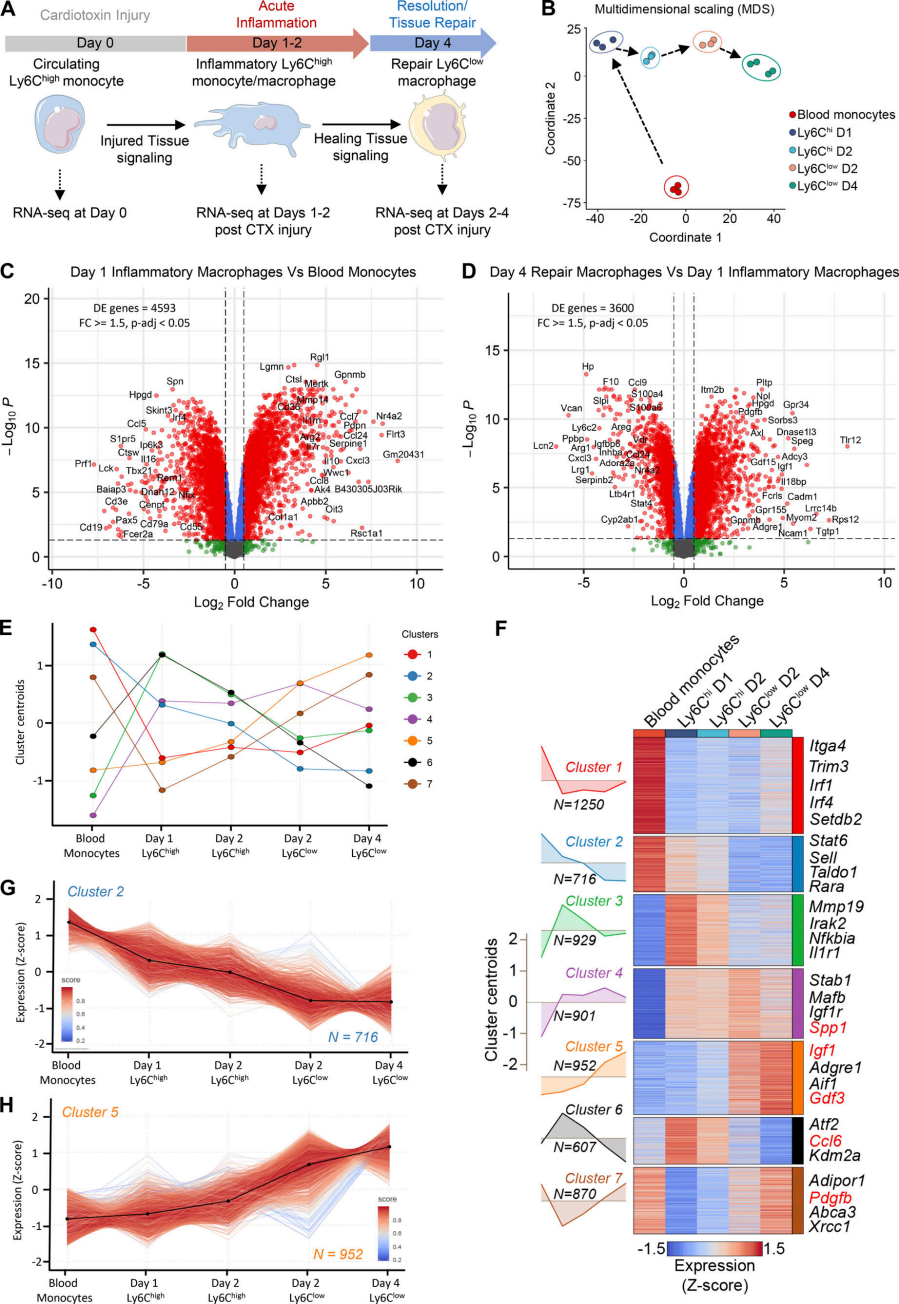
**Transcriptional changes during in situ monocyte to inflammatory and repair MF phenotype transition.** **(A)** Experimental design overview. Experimental setup used to study transcriptional dynamics in WT circulating monocytes and muscle-infiltrating MFs. Cell suspensions were collected from either blood or injured TA muscles, FACS-sorted at indicated time points after CTX injury (gating strategy is shown in Fig. S1, A–D), and subjected to RNA-seq (*n* = 3 or 4 samples per population), followed by downstream analyses. **(B)** Multidimensional scaling plot on normalized mRNA expression values of blood monocytes, Ly6C^high^, and Ly6C^low^ muscle-infiltrating MFs reflecting the overall relationship between datasets. Arrows indicates the developmental trajectory during the injury and regeneration time course. **(C)** Differential gene expression (assessed by RNA-seq) between sorted inflammatory day 1 Ly6C^high^ MFs versus blood monocytes (*n* = 3 biological replicates per group). Gating strategy for the MF subsets isolation is shown in Fig. S1, A and B. A volcano plot (log_2_ FC versus negative log of P value) was used to visualize statistically significant gene expression changes (fold ≥1.5 and adjusted P value <0.05). Statistically significant difference was considered FDR <0.05 from GLM test. Representative top regulated genes are labeled. The number of DE genes is indicated in the upper left corner. **(D)** Differential gene expression (assessed by RNA-seq) between reparatory day 4 Ly6C^low^ versus inflammatory day 1 Ly6C^high^ sorted MF populations (*n* = 3 or 4 biological replicates per group). Gating strategy for the MF subsets isolation is shown in Fig. S1, B and D. A volcano plot (log_2_ FC versus negative log of P value) was used to visualize statistically significant gene expression changes (fold ≥1.5 and adjusted P value <0.05). Statistically significant difference was considered FDR <0.05 from GLM test. Representative top regulated genes are labeled. The number of DE genes is indicated in the upper left corner. **(E)** Line plot showing the dynamics of gene expression and cluster centroids identified by *k-means* in sorted blood monocytes and muscle-infiltrating MFs after CTX injury. **(F)** Heatmap representation of seven defined clusters with differential gene expression (scaled expression; row Z-score) dynamics in blood monocytes and muscle-infiltrating MF populations. Area plots (left) show the overall gene expression dynamics of the clusters (visualized in relation to cluster centroids). The heatmap illustrates all the genes per cluster with representative genes (with high membership scores) for each cluster shown on the right side. Highlighted in red are the genes that translate to secreted proteins as defined in the VerSeDa. **(G and H)** Line plots showing the dynamics of all genes (expression Z-score) within clusters 2 (G) and 5 (H). Centroids are represented with black lines. Color density shows the correlation of a given gene with its centroid. The number of genes within each cluster is shown in the bottom right corner. D, day.

**Figure S1.**
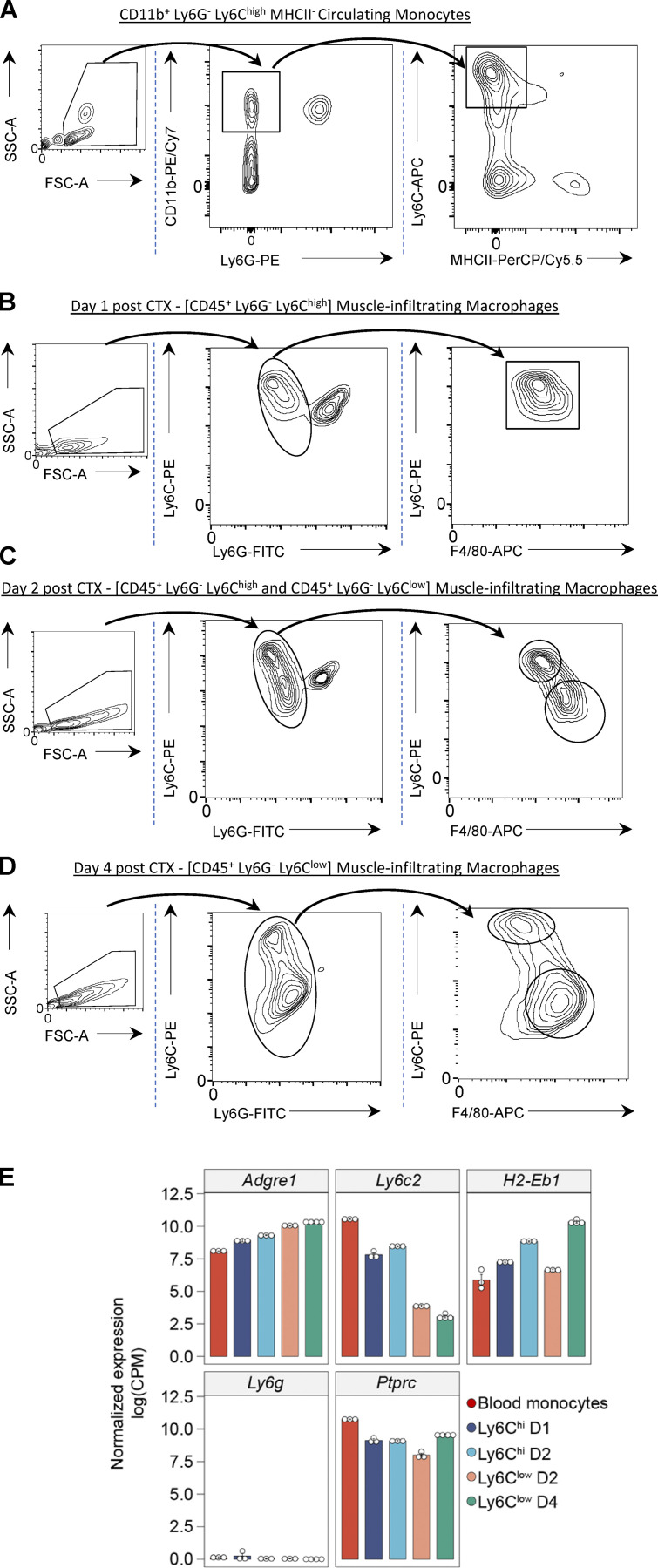
**Circulating monocytes and muscle-infiltrating MFs sorting/gating strategy. (A**) FACS gating strategy for the analysis and sorting of circulating monocytes. Leukocytes were CD45^+^-purified by magnetic bead selection and gated on forward scatter (FSC)/side scatter (SSC) to discriminate live cells, and then markers for CD11b, Ly6G, Ly6C, and MHCII were used to isolate them. x and y axis numbers indicate the fluorescence intensity (on the log_10_ scale) of the indicated fluorescent-labeled antibodies for all the plotted events. FSC and SSC axes are shown in arbitrary linear scale of increasing intensity signal. **(B–D)** FACS gating strategy for the analysis and sorting of MF subsets from CTX-injured muscles at days 1 (B), 2 (C), and 4 (D). Leukocytes were CD45^+^-purified by magnetic bead selection and gated on FSC/SSC to discriminate live cells, and then markers for Ly6G, F4/80, and Ly6C were used to isolate them. x and y axis numbers indicate the fluorescence intensity (on the log_10_ scale) of the indicated fluorescent-labeled antibodies for all the plotted events. FSC and SSC axes are shown in arbitrary linear scale of increasing intensity signal. **(E)** *Adgre1* (*F4/80*), *Ly6c2*, *H2-Eb1* (*MHCII*), *Ly6g*, and *Ptprc* (*CD45*) normalized gene expression (in log_2_[CPM]) in the RNA-seq datasets from sorted MF populations validates the FACS gating and sorting strategy. PE, phycoerythrin; PE/Cy7, PE-Cyanine7; PerCP/Cy5.5, peridinin-chlorophyll-protein complex–Cyanine5.5; APC, allophycocyanin.

To provide an unbiased and robust foundation for our study, we systematically profiled the in situ differentiation of circulating blood monocytes to inflammatory Ly6C^high^ and then to repair-Ly6C^low^ MFs during sterile inflammation and muscle regeneration with the goal of identifying distinct transcriptional patterns across these two transitions (Fig. 1 A; reviewed recently by Chazaud, 2020; Patsalos et al., 2021). In this model, sterile inflammation is caused by a single intramuscular CTX injection, which in turn triggers severe muscle fiber death. The inflammation is accompanied by a rapid and robust infiltration of neutrophils and circulating monocytes, and the generation of MF subpopulations in the regenerating muscle comprising first Ly6C^high^ EGF-like module-containing mucin-like hormone receptor-like 1 (F4/80)^low^ and then Ly6C^low^ F4/80^high^ subsets (Varga et al., 2013; Varga et al., 2016a), which exhibit a dynamic transition in cellular phenotypes (Fig. S1). The robust accumulation of these MFs enabled us to profile these cellular subsets by RNA-seq and extend the gene expression profiles, and analysis of the muscle-infiltrating MFs we obtained previously using microarrays, and CX3CR1 (instead of F4/80) as a marker for infiltrating MFs (Varga et al., 2016a; Varga et al., 2016b). More recent studies by others (Arnold et al., 2015; Jin et al., 2018; Panduro et al., 2018; Iavarone et al., 2020) used a similar gating strategy (Fig. S1) but only for single time points (i.e., day 4 or 5 after CTX injury) or by using different specialized markers like MGL1, CD64, MerTK, and MHCII that characterize only certain aspects of the functional spectrum of infiltrating MFs. The CTX model uses a standard time course (days 1, 2, and 4 after injury) based on convention and experience. Circulating monocytes were sorted (purity >98%) according to their CD11b, Ly6G, Ly6C, and MHCII (*H2-Eb1*) expression (Fig. S1 A) and muscle-infiltrating MFs according to CD45 (*Ptprc*), Ly6C, Ly6G, and F4/80 (*Adgre1*) expression at days 1, 2, and 4 after CTX injury (Fig. S1, B–D). mRNA expression of these markers validates the purity and effectiveness of the sorting and gating strategy (Fig. S1 E). Results obtained previously by our laboratory (Varga et al., 2016a; Varga et al., 2016b; Patsalos et al., 2017; Giannakis et al., 2019) and others (Arnold et al., 2007; Mounier et al., 2013; Panduro et al., 2018) show that inflammation and in particular the numbers of Ly6C^high^ and Ly6C^low^ MFs in regenerating muscle significantly decline after day 4 after injury (Giannakis et al., 2019). During regeneration, the initial Ly6C^high^ F4/80^low^ MF population (Fig. S1 B) rapidly disappears and gets replaced by a population of Ly6C^low^ F4/80^high^ MFs starting at day 2 after injury (Fig. S1 C), whereas the neutrophil infiltration is cleared. 4 d after muscle injury, at a stage that is characterized by active muscle regeneration, the Ly6C^high^ population has almost completely transitioned to a well-defined Ly6C^low^ repair phenotype (Fig. S1 D).

**Figure S2.**
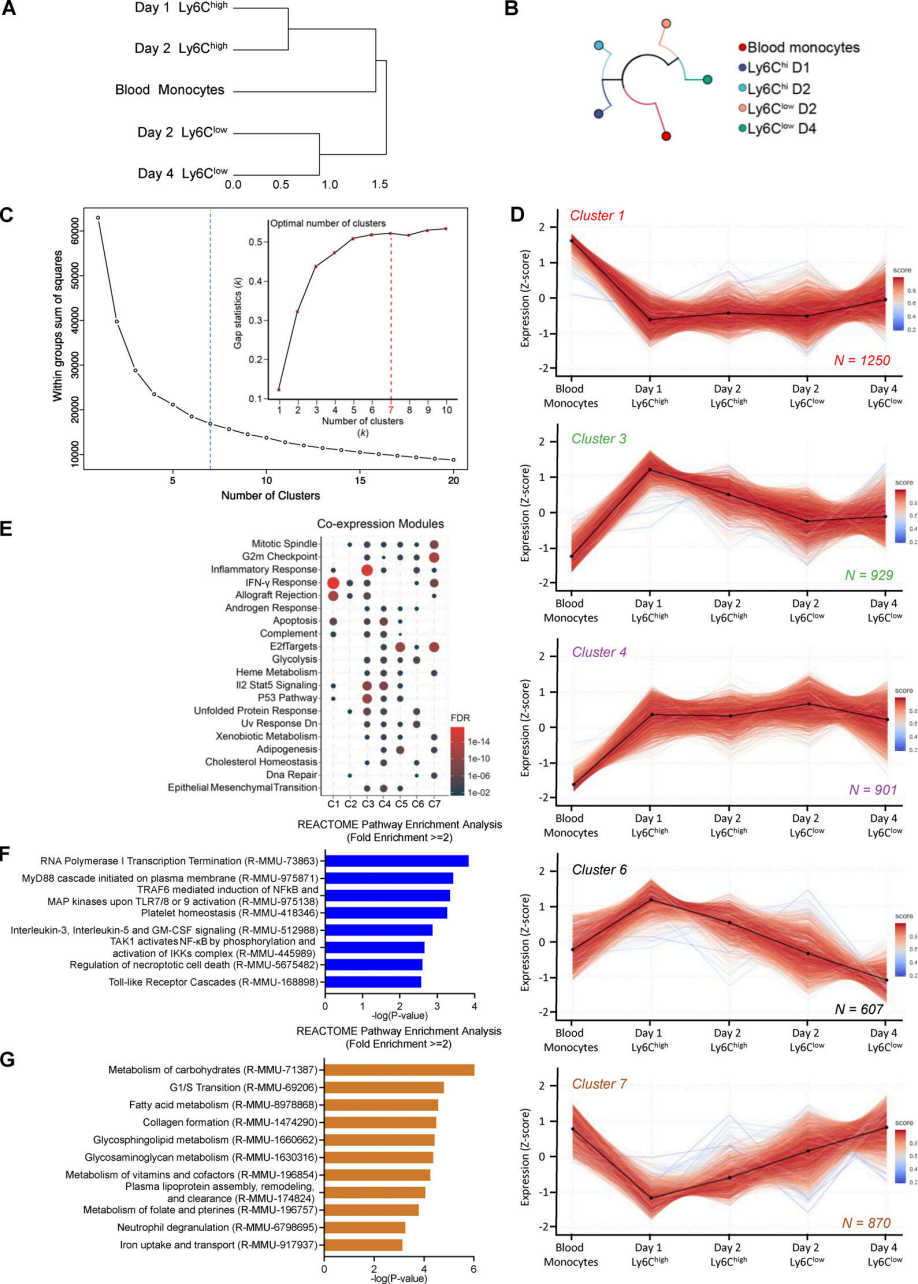
**Muscle-infiltrating MFs RNA-seq clustering (related to** **Fig. 1****). (A)** Hierarchical clustering with distance information on normalized mRNA expression values of blood monocytes, Ly6C^high^, and Ly6C^low^ muscle-infiltrating MFs reflecting the overall relationship between datasets. **(B)** Dendrogram using hierarchical clustering showing the relationship between RNA-seq datasets from blood monocytes, Ly6C^high^, and Ly6C^low^ muscle-infiltrating MFs. **(C)** Elbow-plot of the sum of squares between groups with increasing number of clusters used to determine the optimal number of clusters for *k-means* in the RNA-seq datasets from blood monocytes, Ly6C^high^, and Ly6C^low^ muscle-infiltrating MFs. The optimal number of clusters (*k* = 7) is indicated by the blue dotted line. Inset shows the results of applying the “gap statistic” method for estimating the number of clusters by comparing the change in within-cluster dispersion with that expected under an appropriate reference null distribution (the red dotted line indicates optimal number of clusters, *k* = 7). **(D)** Line plots showing the expression dynamics of all genes per cluster (row Z-score). Centroids are represented with black lines. Color density represents the correlation of a given gene with its centroid. Total number of genes within each cluster is indicated at the bottom right corner. **(E)** Co-expression signature modules detected in clusters 1 to 7 using a hypergeometric gene enrichment workflow (*hypeR* with FDR <0.05 and background population gene set at 25,000). **(F)** Gene enrichment analysis (REACTOME database) of the genes that are part of cluster 2. All terms shown have fold enrichment ≥2 and P value <0.001 (Fisher’s exact test with FDR correction). **(G)** Gene enrichment analysis (REACTOME database) of the genes that are part of cluster 5. All terms shown have fold enrichment ≥2 and P value <0.001 (Fisher’s exact test with FDR correction). C, cluster; D, day.

Principal component analysis revealed that muscle MFs formed well-circumscribed groups, ranked according to their (1) specific sorting markers (Ly6C high/low status) and (2) day of isolation (Fig. 1 B). Day 1 and 2 Ly6C^high^ MFs clustered closer, whereas day 2 and 4 Ly6C^low^ MFs, as well as circulating monocytes, clustered farther apart, corresponding to the proinflammatory phase (days 1–2 after injury), the resolving/repair phase (day 4 after injury) of muscle regeneration, and steady-state (day 0), respectively (Fig. 1 B and Fig. S2 A). Importantly, hierarchical clustering analysis is compatible with the notion that the sorted immune cell lineages may be viewed and interpreted as a hierarchical continuum of cell states (Fig. 1 B and Fig. S2 B), starting with infiltrating circulating monocytes, and ending with repair-type MFs. The comparison between circulating monocytes and Ly6C^high^ and Ly6C^low^ MF subsets at each time point yielded robust changes (Fig. 1, C and D). More specifically, volcano plots of gene expression changes between MF subsets indicate that most of the transcriptional changes occurred (1) between day 0 and day 1 (Fig. 1 C), which corresponds to the maturation from circulating monocytes to Ly6C^high^ infiltrating inflammatory MFs, and (2) between day 1 and day 4 transition from Ly6C^high^ inflammatory to Ly6C^low^ repair MFs (Fig. 1 D; 4,593 and 3,600 differentially expressed [DE] protein-coding genes, respectively), further underscoring that these are the major transitions in the hierarchical continuum of phenotypes (corrected P value < 0.05 and fold change [FC] ≥ 1.5; Table S1). A series of inflammatory molecules (i.e., *Spp1*, *Ptgs2*, *Il10*), or ones associated with promoting myogenesis, such as *Igf1*, are among the top DE genes and show increased expression as regeneration proceeds (Table S1; Bondesen et al., 2004; Uaesoontrachoon et al., 2013; Tonkin et al., 2015; Capote et al., 2016). However, our analysis in Fig. 1 is primarily focused on showing the magnitude and the quantitative and dynamic features of gene expression changes taking place in an unbiased manner, agnostic of gene function. These data (1) confirm that circulating monocytes and inflammatory Ly6C^high^ and repair Ly6C^low^ MFs are clearly different myeloid subsets (validating previous studies using microarrays; Varga et al., 2016b); (2) they represent the extremes of a full spectrum of MF activation states; and (3) they underwent large transcriptomic changes during the time course of sterile physiological inflammation, and particularly during the phenotypic transitions at days 1 and 4 after injury (Fig. 1, C and D).

### Transient and steadily changing transcriptional programs in regenerative inflammation

Next, we sought to identify broad patterns and transitions among the transcriptional changes using clustering. Protein-coding genes were subjected to *k-means* clustering algorithm based on their centered and scaled average expression values (Fig. 1 E), using calculated optimal cluster number (*k* = 7) via gap statistics (Fig. S2 C). Heatmap (Fig. 1 F) and line plots (Fig. 1 E, G, and H; and Fig. S2 D) show the dynamically changing transcriptomic profile of immune cell subsets after CTX injury, and Table S2 provides the gene lists and membership score for each cluster (representative examples are shown in Fig. 1 F). Among the seven clusters, we can distinguish transcriptional programs with transient (clusters 1, 3, 4, 6, and 7) or steadily changing (clusters 2 and 5) dynamics (Fig. 1, E and F). Clusters 1 (red) and 7 (brown) contain 1,250 and 870 protein-coding genes, respectively, which are expressed primarily at the steady-state in circulating monocytes and correspond to transient transcriptional programs that are down-regulated during the early inflammatory phase while they return to baseline during the repair phase (Fig. 1, E and F; and Fig. S2 D). In a reverse fashion, clusters 3 (green) and 6 (black) are composed of 929 and 607 genes, respectively, and correspond primarily to the acute inflammation phase. These clusters present a transient expression pattern with the genes being up-regulated during this phase while later (by day 4) they return to baseline (Fig. 1, E and F; and Fig. S2 D). Similarly, cluster 4 (purple) contains 901 genes that are up-regulated during the early inflammatory phase but then remain unchanged between the muscle-infiltrating subsets through day 4 (Fig. 1, E and F; and Fig. S2 D). Although every cluster identified here represents an opportunity to study the inflammation and regeneration dynamics of MF gene expression (as evidenced by the coexpression modules on Fig. S2 E), we found intriguing the existence of continuous/nontransient changes. Thus, we decided to focus on clusters 2 and 5 (containing 716 and 952 protein-coding genes, respectively) with a steadily increasing or decreasing gene expression pattern (Fig. 1, G and H). We hypothesized that genes in these two clusters contribute in a deterministic way to establish the repair MF lineage identity and can reveal the sensory and regulatory events associated with this cellular phenotype. In fact, our systematic gene set enrichment analysis (GSEA) found that several known regulators and effectors of MF activation/maturation (i.e., *Adgre1*, *Aif1*, *Stat6*, *Rara*, *Sell*) and repair function (i.e., *Igf1*, *Gdf3*; Fig. 1 F) belong to these two clusters, and may play key roles in shaping repair MF identity. Functional classification with gene ontology (GO) analysis (REACTOME pathways database) revealed categories belonging to MF activation, function, metabolism, and immune system regulation (Fig. S2, F and G). Specifically in cluster 2, we observed an enrichment in pathways such as those associated with IL signaling (i.e., *Stat5a*, *Il17ra*, *Csf2ra*, *Il31ra1*, *Il6ra*, *Stat6*), NF-κB activity–regulating pathways (i.e., *Nfkb1*, *Myd88*, *Irak3*, *Map3k6*), regulation of necroptotic cell death and macroautophagy (i.e., *Atg9a*, *Atg4c*, *Mlst8*, *Prkab2*, *Tomm5*), and platelet homeostasis (i.e., *Pecam1*, *Itpr1*, *Fgr*; Fig. S2 F). In cluster 5, we observed an enrichment in innate immune activation (i.e., *C1qa*, *C1qc*, *Cd4*, *Cd81*, *Ctsa*, *Itgax*, *Tlr8*, *Tlr1*, *Tlr3*, *Tlr12*, *Trem2*, *Fcgr4*), neutrophil degranulation (i.e., *Folr2*, *Sirpa*, *Psap*, *Alad*), lipid, carbohydrate, and vitamin metabolism (i.e., *Mdh1*, *Lyve1*, *Slc25a10*, *Tkfc*, *Ndst1*, *Galns*, *Cspg4*, *Bgn*, *Apoe*, *Lpl*, *Vkorc1*, *Hexa*, *Ltc4s*, *Hpgds*), collagen biosynthesis (i.e., *Col1a2*, *Col11a2*, *Col15a1*, *Col1a1*, *Col3a1*, *P3h1*, *Colgalt1*, *Crtap*), iron metabolism (i.e., *Slc40a1*, *Hmox2*, *Slc46a1*, *Atp6v0d2*), and cell cycle phase transition (i.e., *Mcm4*, *Cdk1*, *Pola1*; Fig. S2 G). Thus, focusing on the top DE genes that follow cluster’s 2 and 5 kinetics could reveal novel regulators and effectors that establish and maintain the repair/resolution phases of the MF-mediated regeneration process in a continuous fashion without necessarily being involved in the early inflammatory phase of the process.

**Figure S3.**
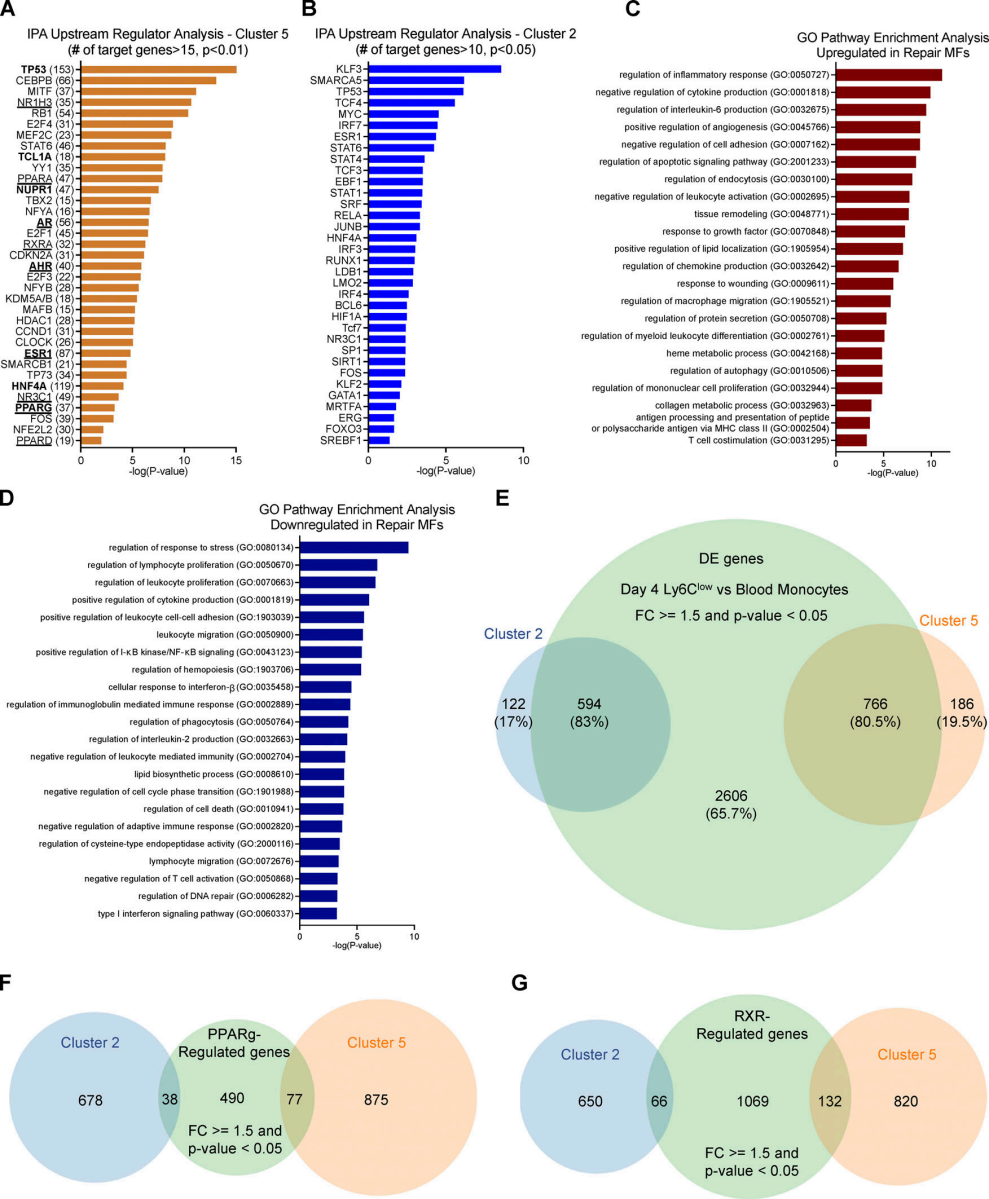
**GO pathway analysis of DE genes between blood monocytes and muscle-infiltrating repair MFs (related to** **Figs. 2**** and ****5****). (A)** IPA Upstream Regulator Analysis on genes participating in cluster 5. Top 35 transcriptional regulators are shown, ranked based on P value (P < 0.01; Fisher’s exact test). In parentheses are the number of known target genes included in the cluster. Highlighted in bold are the regulators predicted to target *Gdf15* based on the literature (IPA Knowledge Base). Underline indicates the ligand-dependent nuclear receptors. **(B)** IPA Upstream Regulator Analysis on genes participating in cluster 2. Top 35 transcriptional regulators with at least 10 known target genes in the cluster are ranked based on P value (P < 0.05; Fisher’s exact test with FDR correction). **(C)** Gene enrichment/GO analysis of the up-regulated genes in day 4 reparatory Ly6C^low^ MFs versus circulating monocytes. All terms shown have P value <0.001 (Fisher’s exact test with FDR correction). **(D)** Gene enrichment/GO analysis of the down-regulated genes in day 4 reparatory Ly6C^low^ MFs versus circulating monocytes. All terms shown have P value <0.001 (Fisher’s exact test with FDR correction). **(E)** Venn diagram illustrating the overlap of DE genes (FC ≥1.5 and P value <0.05) in day 4 reparatory Ly6C^low^ MFs versus circulating monocytes and genes belonging to clusters 2 and 5 following the *k-means* classification. Percentage of overlap and number of genes are shown. Statistically significant difference was considered FDR <0.05 from GLM test. **(F)** Venn diagram illustrating the overlap of PPARγ regulated genes in muscle-infiltrating MFs (FC ≥1.5 and P value <0.05 determined by *hypeR*) and genes belonging to clusters 2 and 5 following the *k-means* classification. The number of genes is shown. The microarray dataset of PPARγ null muscle-infiltrating MFs used for this analysis is publicly available (GEO accession no. GSE71155; Varga et al., 2016b). **(G)** Venn diagram illustrating the overlap of RXR-regulated genes in unstimulated BMDMs (FC ≥1.5 and P value <0.05 determined by *hypeR*) and genes belonging to clusters 2 and 5 following the *k-means* classification. The number of genes is shown. The RNA-seq datasets of RXR-null BMDMs used for this analysis are deposited under GEO accession no. GSE164722.

In addition, the genes sharing the same expression kinetics in clusters 2 and 5 may be regulated by the same or similar regulators, in particular transcription factors (TFs). Thus, in a complementary analysis and to further illuminate the biological activities represented in clusters 2 and 5, we used the Ingenuity Pathway Analysis (IPA) Upstream Regulator Analysis to identify the cascade of potential upstream transcriptional regulators that could explain the observed gene expression kinetics. This analysis examines how many known targets of each transcription regulator are present in our clusters. We identified 86 upstream transcriptional regulators with at least 15 known regulated target molecules in cluster 5, including several ligand-dependent nuclear receptors such as PPARα/δ, NR1H3, AR, RXRa, AHR, ESR1, NR3C1, and PPARγ (Fig. S3 A, underlined) and other transcriptional regulators involved in proliferation/cell cycle (TP53, HNF4a, NUPR1, TBX2, CDKN2A, E2F4), and inflammation/MF maturation (AP-1 factors, CEBPB, MAFB, NFE2L2, STAT6, RB1, SMARCB1, TCL1A, E2F1, MITF, YY1, HDAC1, and KDM5; Fig. S3 A). Similarly, in cluster 2, we identified 65 upstream transcriptional regulators, some observed in the previous analysis, such as TP53, HNF4a, STAT family members, and others such as IFN regulatory factor (IRF) and Krüppel-like factor family members, FOXO3, RUNX1, GATA1, and SP1 (Fig. S3 B). Next, we prioritized and grouped the identified genes for further analyses, focusing on potential new effectors.

### Identification of GDF-15 as a prototypic and novel secreted effector in Ly6C^low^ repair MFs

**Figure 2.**
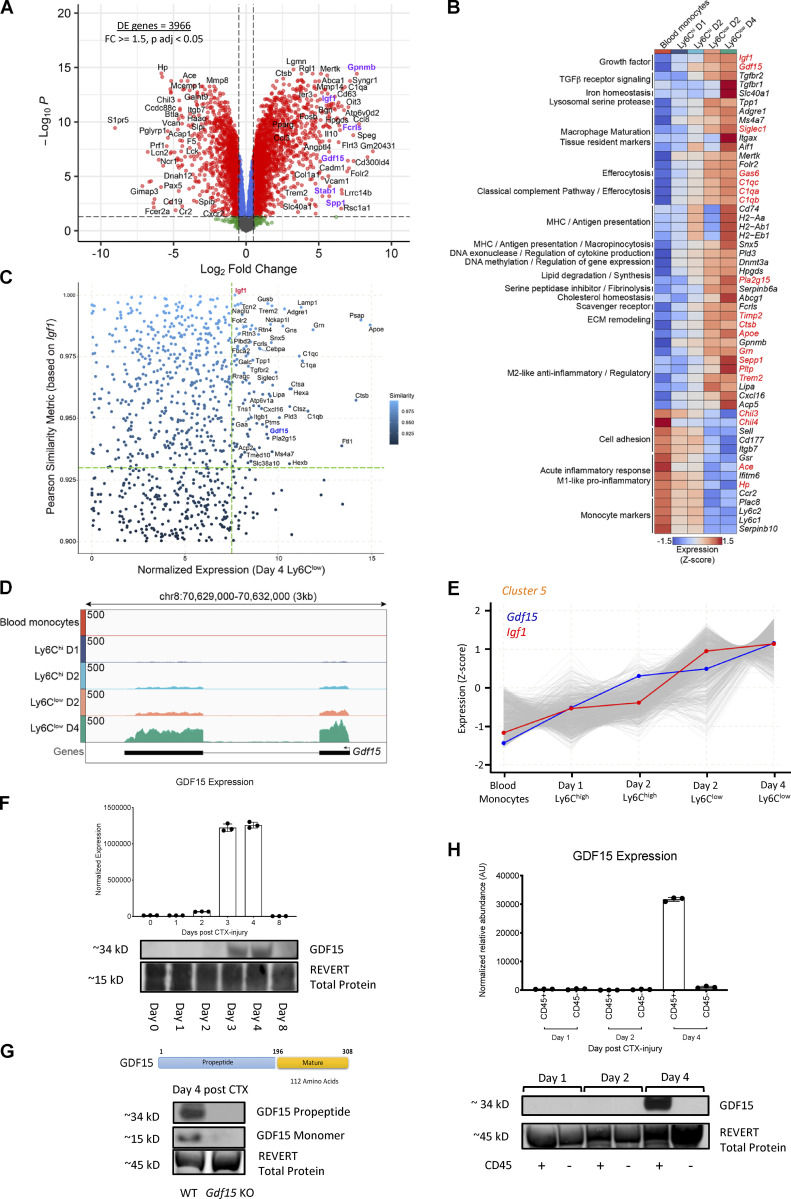
**Identification of GDF-15 as a novel effector in muscle-infiltrating MFs during regeneration. (A)** Differential gene expression (assessed by RNA-seq) between repair day 4 Ly6C^low^ MFs versus blood monocytes. A volcano plot (log_2_ FC versus negative log of P value) was used to visualize statistically significant gene expression changes (fold ≥1.5 and adjusted P value <0.05). Representative top regulated genes are labeled in black. Highlighted in purple labels are known scavenger receptors and inflammation/repair-related genes. The number of DE genes is indicated in the upper left corner. **(B)** Heatmap showing the mRNA expression pattern of the top 50 genes passing a set of criteria (1) being DE in the analysis in A, (2) included in cluster 2 or 5, and (3) high expression (in CPM reads mapped) in blood monocytes, Ly6C^high^, or Ly6C^low^ muscle-infiltrating MFs. RNA-seq expression values are visualized as normalized expression (log_2_[CPM]), and each gene shown is clustered into functional categories. Highlighted in red are the genes that translate to secreted proteins defined in the VerSeDa. **(C)** Dot plot showing genes with similar trend and rate to *Igf1* (Pearson similarity 0.9–1.0). Labeled genes have Pearson similarity >0.93 and high normalized expression (log_2_[CPM] >7.5] in day 4 Ly6C^low^ repair MFs. *Igf1* and *Gdf15* are highlighted in red and blue, respectively, and green dotted lines indicate the labeling cutoffs. **(D)** Genome browser view of the *Gdf15* locus from blood monocytes, Ly6C^high^, and Ly6C^low^ muscle-derived MFs RNA-seq datasets. **(E)** Line plot illustrating the gene expression dynamics of all genes (row Z-score) within cluster 5 in blood monocytes, Ly6C^high^, and Ly6C^low^ muscle infiltrating MFs. The expression dynamics of *Gdf15* and *Igf1* are highlighted with blue and red, respectively. **(F)** GDF-15 protein expression in whole-muscle lysates of regenerating TA muscles from WT male mice at indicated time points. Three biological replicates are quantified for each time point with normalized values to total protein of each sample (*n* = 3 per time point). REVERT total protein was used for loading control and signal normalization. **(G)** GDF-15 protein expression (in propeptide and monomer form) in whole-muscle lysates of regenerating muscles from WT and *Gdf15 KO* mice at day 4 after CTX. Top: An illustration of GDF-15 peptide structure. REVERT total protein was used for loading control and signal normalization. **(H)** GDF-15 protein expression in CD45^+^ and CD45^−^ cells isolated at indicated time points after CTX injury from WT mice (*n* = 3 per time point). REVERT total protein was used for loading control and signal normalization.

The complete longitudinal time course analysis allowed us to identify the unexpected, steadily changing clusters of genes (C2 and C5) and an enrichment of secreted growth regulating factors in the latter (Fig. 1 F). Next, we decided to follow up this lead and carry out a direct comparison of repair Ly6C^low^ cells to circulating monocytes, which is its precursor. We argued that this analysis can provide insights into how nontransient lineage transcriptional programs establish the repair MF subset from a naive monocytic state (Fig. 2 A). The up-regulated genes in this comparison (Table S1) were associated with protein secretion, the regulation of endothelial cell proliferation, and GO categories related the late steps of regeneration such as response to growth factors, wounding, tissue remodeling, endocytosis, autophagy, leukocyte differentiation, inflammation (IL-6, TNF, and regulation of TGF-β pathways), and negative regulation of leukocyte migration, adhesion, and apoptosis (Fig. S3 C). This analysis also showed down-regulation of genes associated with early stages of regeneration, such as leukocyte migration, adhesion, cell motility, necrotic cell death, and intracellular signal transduction associated with immune responses and cell communication (regulation of cytokine production; response to IL-2, and IFN-γ; Fig. S3 D). Interestingly, the top genes that were up-regulated at day 4 in the Ly6C^low^F4/80^high^ MFs versus blood monocytes comparison are known inflammation/repair secreted effector molecules (i.e., *Igf1*, *Gdf15*, *Spp1*, *Gpnmb*; Uaesoontrachoon et al., 2013; Tonkin et al., 2015; Capote et al., 2016; Silva et al., 2018) and scavenger receptors (i.e., *Stab1*, *Fcrls*; Fig. 2 A; Palani et al., 2011; Rantakari et al., 2016). This observation is entirely consistent with the role of Ly6C^low^ repair MFs in the initiation of the resolution and repair phase of the inflammatory response following injury. To prioritize this extensive DE gene list (3,966 genes, FC ≥ 1.5) and identify new pathways that could impact MF identity, we overlapped it with the genes identified previously in clusters 2 and 5. We found 766 genes from cluster 5 (80.5% overlap) and 594 genes from cluster 2 (83% overlap) belonging to this DE gene list (Fig. S3 E), validating the approach. Next, we filtered this list based on high levels of expression in either the blood monocytes (represented by cluster 2) or Ly6C^low^ repair MFs of day 4 (represented by cluster 5). The top 50 genes passing our criteria were manually curated and grouped into functional categories (Fig. 2 B). Among these genes, we found a series of molecules involved in the interactions with adaptive immunity/antigen presentation (*Cd74*, *H2-Aa*, *H2-Ab1*, *H2-Eb1*, *Snx5*), anti-inflammatory/regulatory factors (*Apoe*, *Sepp1*, *Grn*, *Pltp*, *Trem2*, *Lipa*, *Cxcl16*, *Acp5*, *Chil3*, *Chil4*, *Gpnmb*), secreted growth factors (*Igf1*, *Gdf15*), effectors and enzymes involved in iron (*Slc40a1*) and lipid/cholesterol (*Pla2g15*, *Abcg1*, *Hpgds*) homeostasis, lysosomal proteases (*Tpp1*), DNA methylation (*Dnmt3a*), exonucleases (*Pld3*), extracellular matrix remodeling (*Timp2*, *Ctsb*), as well as receptors involved in TGF-β signaling (*Tgfbr2*, *Tgfbr1*), scavenging (*Fcrls*), efferocytosis (*Gas6*, *C1qc*, *C1qa*, *C1qb*), and importantly MF maturation/tissue resident markers (*Adgre1*, *Ms4a7*, *Siglec1*, *Itgax*, *Aif1*, *Mertk*, *Folr2*). We also observed a substantial decrease in the expression of genes involved in cell adhesion (*Sell*, *Cd177*, *Itgb7),* acute phase/pro-inflammatory responses (*Gsr*, *Ace*, *Ifitm6*, *Hp*), and monocyte identity markers (*Ccr2*, *Ly6c2*, *Ly6c1*, *Serpinb10*, *Plac8*), as expected (Fig. 2 B). Using the UniProtKB and Vertebrate Secretome Database (VerSeDa) mouse protein databases (we considered records with information extracted from literature and curator-evaluated computational analysis), we could further curate 19 of these genes into molecules with reported secreted effector function like *Gdf15*, *Igf1*, *Siglec1*, *Gas6*, *C1qc*, *C1qa*, *C1qb*, *Pla2g15*, *Timp2*, *Ctsb*, *Apoe*, *Sepp1*, *Grn*, *Pltp*, *Trem2*, *Chil3*, *Chil4*, *Ace*, and *Hp* (Fig. 2 B, highlighted in red).

A well-established effector in the context of MF-mediated muscle repair is IGF-1. IGF-1 is a growth factor secreted by repair MFs and is a potent enhancer of tissue regeneration (Lu et al., 2011). It is involved in the activation, proliferation, and differentiation of satellite cells (Hill and Goldspink, 2003; Mourkioti and Rosenthal, 2005), but can also act as a key factor in the resolution of inflammation and the inflammatory to repair MF phenotype switch during muscle injury and regeneration (Tonkin et al., 2015). To date, this is the only secreted factor with a bivalent role in sterile inflammation and tissue repair, by acting in both a paracrine and an autocrine manner. Thus, in an independent analysis and to identify targets with a similarly strong predictive power for repair Ly6C^low^ MF functionality, we performed a Pearson similarity metric analysis to find genes that follow similar expression trend and rate to *Igf1*, through the entire course of regeneration (similarity cutoff set at ≥0.9). Altogether, 918 genes were identified (Fig. 2 C), among which were 26 genes from the above top-ranked gene list (Fig. 2 B), which is essentially the molecular signature of Ly6C^low^ repair MFs. At the top of these lists, the only other secreted growth factor was *Gdf15* (Fig. 2 D); that is also a member of the TGF-β superfamily like IGF-1 (Bootcov et al., 1997; Lawton et al., 1997), with Pearson similarity of 0.937 (Fig. 2 C) and almost identical expression levels and kinetics to *Igf1* (Fig. 2, B and E). At the protein level, GDF-15 becomes detectable at day 3 after CTX injury and peaks at day 4 in whole-muscle lysates (Fig. 2 F), both as a propeptide and as a mature monomer (Fig. 2 G). The protein expression closely followed the induction seen at the mRNA level in MFs of day 4 (Fig. 2 D), at a time when inflammation subsides, and regenerative processes start to dominate within the injured muscle. Notably, the induction of GDF-15 expression was detectable only in the CD45^+^ (hematopoietic) compartment, suggesting that CD45^+^ cells are the sole local source of active GDF-15 during the regeneration process (Fig. 2 H). It is also important to note that the GDF-15 protein induction during CTX injury was undetectable in muscle samples from *Gdf15* KO animals (Fig. 2 G), validating our detection method and reagents. Based on these findings, GDF-15’s role in MF-mediated regeneration warranted further investigation. We hypothesized that GDF-15 could be a novel repair MF–derived factor acting similarly to IGF-1 by influencing the outcome of skeletal muscle regeneration either as a regulator of repair MF function and/or as an effector/growth factor acting on the muscle tissue itself.

### GDF-15 is required for proper muscle regeneration

**Figure 3.**
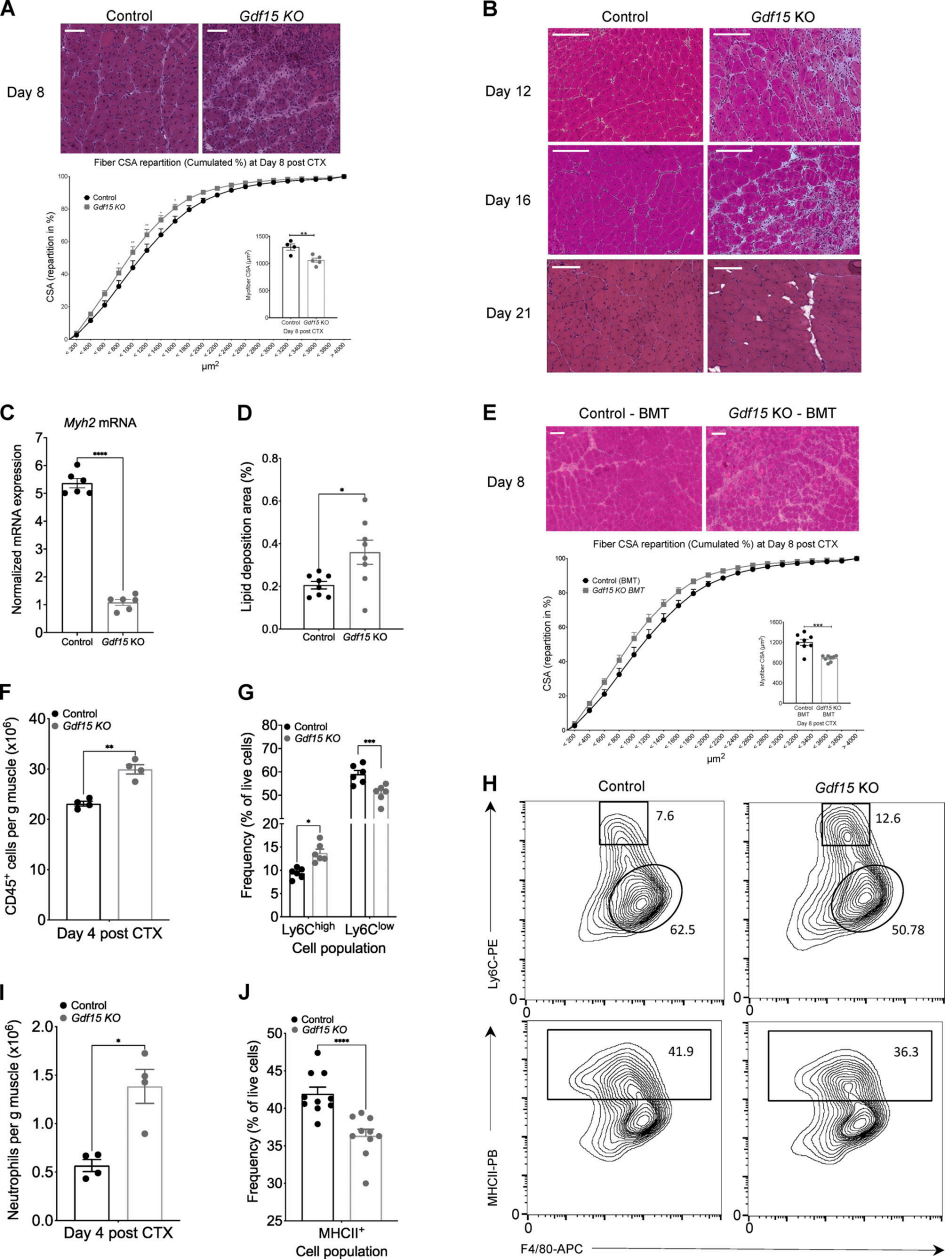
**GDF-15 deficiency leads to impaired muscle regeneration and impacts the MF phenotype switch.** **(A)** Top: Representative images of H&E-stained skeletal muscle (TA) from WT-control and *Gdf15 KO* animals at day 8 after CTX-induced injury. Scale bars in the upper left corner represent 100 µm. Bottom: Fiber size repartition of regenerating muscle in WT-control and *Gdf15 KO* animals at day 8 after CTX injury (two-way ANOVA with multiple comparison test). Inset shows the average fiber CSA of regenerating muscle at day 8 after CTX injury (*n* = at least 4 mice per group). **(B)** Representative images of H&E-stained skeletal muscle (TA) from WT-control and *Gdf15 KO* animals at days 12, 16, and 21 after CTX-induced injury. Scale bars in the upper left corner represent 100 µm. **(C)** *Myh2* mRNA expression in WT-control and *Gdf15 KO* muscles at day 8 after CTX injury (*n* = 6 muscles per group). *Myh2* was normalized over *Rpl32* (*n* = 3 independent experiments). **(D)** Percentage of ectopic lipid deposition relative to the muscle regeneration area at day 21 of regeneration in WT-control and *Gdf15 KO* muscles is shown (*n* = 8 mice per group). **(E)** Top: Representative images of H&E-stained TA skeletal muscle 8 d after CTX injury from chimeric WT BoyJ BMT animals (CD45.1 recipients) that received either WT (CD45.2) or *Gdf15 KO* BM. Scale bars in the upper left corner represent 100 µm. Bottom: Cumulated myofiber CSA repartition (two-way ANOVA with multiple comparison test) and mean CSA (inset) at day 8 after CTX injury from BMT animals (*n* = at least 8 mice per group, two-way ANOVA). **(F)** Number of infiltrating myeloid (CD45^+^) cells in regenerating muscle from WT-control and *Gdf15 KO* muscles at day 4 after CTX injury (*n* = 4 mice per group). **(G)** Frequency (in %) of CD45^+^ inflammatory (Ly6C^high^ F4/80^low^) and repair (Ly6C^low^ F4/80^high^) MFs from WT-control and *Gdf15 KO* mice at day 4 following CTX injury (*n* = 6 mice per group). **(H)** Representative flow cytometry 10% quantile contour plots of inflammatory and repair MFs from WT-control and *Gdf15 KO* at day 4 after CTX injury. Shapes indicate the gating used for cell frequency quantification (square = Ly6C^high^ inflammatory MFs, circle = Ly6C^low^ repair MFs, rectangle = MHCII^+^ MFs). Representative frequencies for each cell population are shown adjacent on inside each gate. x and y axis numbers indicate the fluorescence intensity (on the log_10_ scale) of the indicated fluorescent-labeled antibodies for all the plotted events. PB, Pacific Blue; APC, allophycocyanin. **(I)** Number of infiltrating neutrophils (CD45^+^ Ly6G^+^ Ly6C^int^ F4/80^−^) cells in regenerating muscle from WT-control and *Gdf15 KO* muscles at day 4 after CTX injury (*n* = 4 mice per group). **(J)** Frequency (in %) of CD45^+^ F4/80^+^ MHCII^+^ MFs from WT-control and *Gdf15 KO* mice at day 4 following CTX injury (*n* = 10 mice per group). In all bar graphs, bars represent mean ± SEM. Exact P values were determined using unpaired Student’s *t* test unless otherwise noted. *, P < 0.05; **, P < 0.01; ***, P < 0.001; ****, P < 0.0001. PE, phycoerythrin.

**Figure S4.**
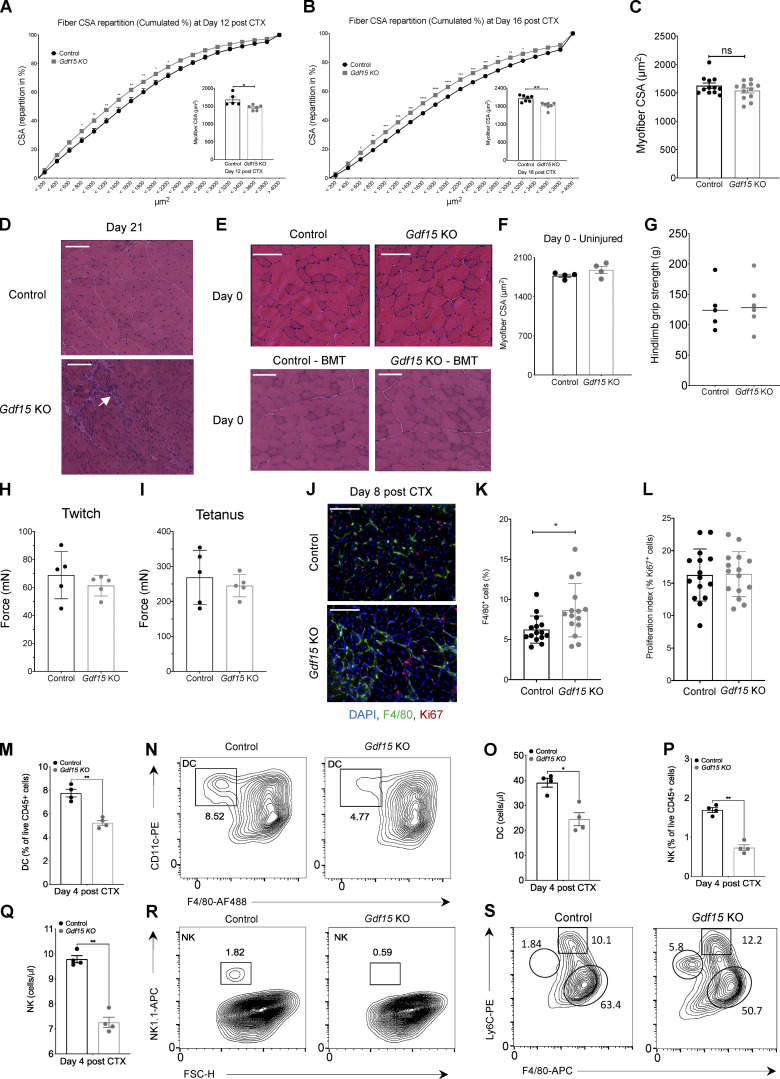
**GDF-15 ablation allows normal muscle development and muscle growth in uninjured animals (related to** **Fig. 3****). (A and B)** Fiber size repartition of regenerating muscle in WT-control and *Gdf15 KO* mice at days 12 (A) and 16 (B) after CTX injury (two-way ANOVA with multiple comparison test). Insets show the average fiber CSA of regenerating muscle at indicated time points after CTX injury (*n* = at least 5 mice per group). **(C)** Average fiber CSA of regenerating muscle in WT-control and *Gdf15 KO* mice at day 21 after CTX injury (*n* = 12 muscles per group). **(D)** Representative images of increased infiltration in H&E-stained skeletal muscle (TA) from WT-control and *Gdf15 KO* animals at day 21 after CTX-induced injury. Arrow indicates area of persistent immune cell infiltration. Scale bars in the upper left corner represent 100 µm. **(E)** Top: Representative images of H&E-stained skeletal muscle (TA) from uninjured WT-control and *Gdf15 KO* animals. Bottom: Representative images of H&E-stained skeletal muscle (TA) from uninjured control–BMT and *Gdf15 KO*–BMT chimeras. Scale bars in the upper left corner represent 100 µm. **(F)** Average fiber CSA of uninjured muscle in WT-control and *Gdf15 KO* animals (*n* = 4 mice per group). **(G)** In vivo hindlimb grip strength in uninjured WT-control and *Gdf15 KO* adult male mice. Mean of five measurements per mouse is plotted (*n* = 6 mice per group). **(H)** Quantification of in vivo muscle twitch force in uninjured WT-control and *Gdf15 KO* mice (*n* = 5 mice per group). **(I)** Quantification of in vivo muscle tetanus force in uninjured WT-control and *Gdf15 KO* mice (*n* = 5 mice per group). **(J)** Representative immunofluorescence images of regenerating muscles in WT-control and *Gdf15 KO* animals at day 8 after injury (red marks proliferation marker Ki67, green marks MF marker F4/80, and blue indicates nuclei). Scale bars in the upper left corner represent 100 µm. **(K)** Increased presence of F4/80^+^ cells in the *Gdf15 KO* at day 8 after CTX. Values are expressed as percentage of total cells (*n* = 15 representative fields of view per group). **(L)** Quantification shows the proliferation index of F4/80^+^ cells. The values represent the percentage of Ki67^+^ cells over F4/80^+^ cells in the respective field of view (*n* = 15 representative fields of view per group). **(M)** Frequency (in %) of DCs from WT-control and *Gdf15* KO mice at day 4 following CTX injury (*n* = 4 animals per group). x and y axis numbers indicate the fluorescence intensity (on the log_10_ scale) of the indicated fluorescent-labeled antibodies for all the plotted events. **(N)** Representative FACS contour plots of DCs (gated as CD45^+^ CD11c^+^ F4/80^−^ Ly6G-MHCII^+^) at day 4 after CTX in WT-control and *Gdf15* KO animals. x and y axis numbers indicate the fluorescence intensity (on the log_10_ scale) of the indicated fluorescent-labeled antibodies for all the plotted events. AF488, Alexa Fluor 488. **(O)** Absolute number of infiltrating DCs in regenerating muscle from WT-control and Gdf15 KO muscles at day 4 after CTX injury using counting beads (*n* = 4 animals per group). **(P)** Frequency (in %) of NK cells from WT-control and *Gdf15* KO mice at day 4 following CTX injury (*n* = 4). **(Q)** Absolute number of infiltrating NK cells in regenerating muscle from WT-control and *Gdf15* KO muscles at day 4 after CTX injury using counting beads (*n* = 4 animals per group). **(R)** Representative FACS analysis of NK cells (gated as CD45^+^ F4/80^−^ Ly6G^−^ Nk1.1^+^) at day 4 after CTX in control and *Gdf15* KO animals. y axis numbers indicate the fluorescence intensity (on the log_10_ scale) of the indicated fluorescent-labeled antibody for all the plotted events. x axis scale is an arbitrary linear scale representing increasing intensity of forward scatter (FSC) signal. APC, allophycocyanin. **(S)** Representative flow cytometry contour plots of inflammatory and repair MFs (without excluding Ly6G^+^ cells) from WT-control and *Gdf15 KO* at day 4 after CTX injury. Shapes indicate the gating used for cell frequency quantification (circle = neutrophils, square = Ly6C^high^ inflammatory MFs, oval = Ly6C^low^ repair MFs). Representative frequencies for each cell population are shown adjacent on inside each gate. x and y axis numbers indicate the fluorescence intensity (on the log_10_ scale) of the indicated fluorescent-labeled antibodies for all the plotted events. APC, allophycocyanin; PE, phycoerythrin. In all graphs, bars and lines represent mean ± SEM. Exact P values were determined using unpaired Student’s *t* test unless otherwise noted. *, P < 0.05; **, P < 0.01.

To assess the role of GDF-15 during muscle regeneration, we used the CTX injury model and used an established genetic GDF-15 ablation model (Hsiao et al., 2000). In this model, muscle regeneration was severely impaired at day 8 after CTX, in comparison to control muscles as shown by histological analysis (Fig. 3 A, top). Morphometric analysis validates this impairment, as illustrated by a shift to the left (toward small fiber sizes) of the distribution of the myofiber cross-sectional area (CSA; Fig. 3 A, bottom), a 19% decrease in the mean CSA of regenerating myofibers (Fig. 3 A, inset), and a decrease in myosin heavy chain 2 (*Myh2*) expression (Fig. 3 C), all indicative of an impairment in regenerating myocyte organization and fiber content. Next, we wanted to determine whether regeneration was still impaired at later stages of the process in the *Gdf15* KO. Intriguingly, both at day 12 and day 16 after CTX, the *Gdf15* KO failed to recover to control levels, as illustrated by histological analysis (Fig. 3 B), a shift to the left of the distribution of the myofiber CSA (Fig. S4, A and B), and a 13.3% or 10.8% decrease in the mean CSA of regenerating myofibers at day 12 and day 16, respectively (Fig. S4, A and B, insets). Although no significant difference in the CSA was observed at day 21 after injury (Fig. S4 C), we did observe a significant increase in ectopic lipid accumulation (Fig. 3, B and D) and cell infiltration (Fig. S4 D), both being hallmarks of defective muscle regeneration. It is important to note that no preexisting developmental musculature impairment was observed in myeloid or full-body *Gdf15* KO uninjured muscles (day 0), as assessed by histological analysis (Fig. S4 E), fiber CSA measurement (Fig. S4 F), hindlimb grip strength (Fig. S4 G), and in vivo force measurements (Fig. S4, H and I), suggesting that GDF-15 is not required for embryonic muscular development and that the muscle regeneration/growth impairment is only evident after an acute injury.

### Myeloid GDF-15 impacts both infiltration and phenotypic transition of MFs following CTX injury

To exclude the involvement of confounding or compensatory mechanisms in other tissue compartments and to determine whether GDF-15 deficiency in the hematopoietic/myeloid compartment is the major contributor to the observed delayed regeneration phenotype, we generated chimeric animals reconstituted with *Gdf15 KO* bone marrow (BM). In this model, BM from *Gdf15 KO* or control mice was used to reconstitute the hematopoietic compartment of total body–irradiated WT-control animals. Compared with animals that received WT BM, GDF-15–deficient BM chimeras exhibited a profound impairment in regeneration at day 8 after injury (Fig. 3 E), similar to the full-body *Gdf15 KO* (Fig. 3 A). When compared with WT BM-transplanted (BMT) animals, *Gdf15 KO* chimeras contained more regenerating myofibers with smaller CSA as illustrated by a shift to the left (toward small fiber sizes) of the distribution of the myofiber CSA (Fig. 3 E, bottom), and a 24.5% decrease in the mean CSA of regenerating myofibers (Fig. 3 E, inset). Altogether, the results from the two distinct loss-of-function genetic models and the high expression of GDF-15 in the repair Ly6C^low^ MF compartment of the hematopoietic niche indicated that myeloid-derived GDF-15 critically contributes to muscle regeneration.

Next, we asked whether the impaired muscle regeneration was caused by a defect in the cellular dynamics of the myeloid cell infiltrate during muscle regeneration. The regenerative areas contained increased inflammatory infiltrations (F4/80^+^ cells) in *Gdf15 KO* muscles at day 8, as assessed by immunohistochemistry (Fig. S4, J and K), and were independent of local proliferation (Fig. S4 L), suggesting that the infiltration and resolution of inflammation were impaired. Interestingly, we also observed differences in the numbers of invading myeloid cells (CD45^+^) at day 4 after CTX injury using CD45^+^ magnetic bead selection (Fig. 3 F), which is in line with GDF-15’s role in regulating immune cell infiltration (Kempf et al., 2011). However, this finding did not exclude the possibility of a change in the cellular composition and subtype specification of the infiltrating myeloid cells as well. Since *Gdf15* is expressed highly in repair MFs, we decided to follow the differentiation dynamics of MFs at day 4, as the observed effect of GDF-15 deficiency must derive from these MFs subsets. Therefore, we examined the dynamics of the infiltrating myeloid cell populations (inflammatory Ly6C^high^ F4/80^low^ and repair Ly6C^low^ F4/80^high^ MFs) during the regeneration phase by flow cytometry (Fig. 3, G and H). Ly6C^high^ inflammatory MFs are progressively differentiating into Ly6C^low^ repair MFs by day 4 after CTX injury (Fig. S1, B–D). In the case of the *Gdf15 KO*, the frequencies of both inflammatory Ly6C^high^ F4/80^low^ and Ly6C^low^ F4/80^high^ repair MFs were skewed compared with controls (Fig. 3, G and H, top), suggesting a decreased conversion of inflammatory to repair MFs. Although the observed alteration in the proportion of MFs was significant, it must be noted that the ratio and absolute numbers (Fig. 3 I and Fig. S4 S) of infiltrating neutrophils (Ly6G^+^ F4/80^−^Ly6C^int^) were also significantly higher in the absence of GDF-15, further suggesting that the proper clearance of neutrophils and the overall resolution of inflammation was altered. Concurrently, the recruitment of dendritic cells (DCs; Fig. S4, M–O) and natural killer (NK) cells (Fig. S4, P–R) is reduced at day 4 after CTX in the *Gdf15* KO. These findings indicate that the dynamics of immune cell recruitment are significantly impacted in the *Gdf15* KO.

Assessing more functional markers, such as the MHCII molecules, which were incorporated recently as an alternative gating strategy for muscle-infiltrating MFs (Panduro et al., 2018), we observed a decrease in the ratio of MHCII^+^ F4/80^high^ cells at day 4 (Fig. 3, J and H, bottom), suggesting a potential impairment in the antigen-presenting capacity of these cells. Collectively, these results show that GDF-15 deficiency has quantitatively and qualitatively affected myeloid cells’ infiltration and in situ differentiation and reveal a critical role for myeloid-secreted GDF-15 as a potent effector and coordinator of the resolution of inflammation in regenerating muscle.

### MF-secreted GDF-15 regulates myoblast proliferation and influences MF antigen-presenting capacity

**Figure 4.**
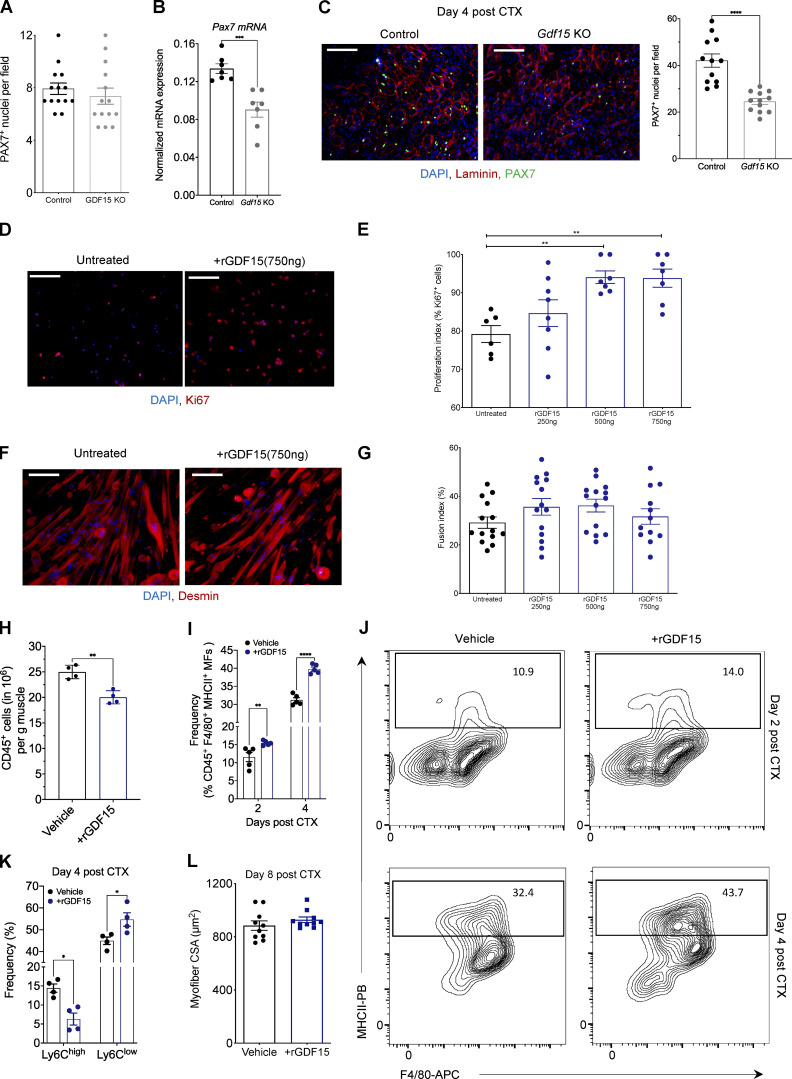
**Effects of recombinant GDF-15 on muscle progenitor proliferation in vitro and MHCII expression of muscle MFs in vivo*.* (A)** Number of PAX7^+^ cells in uninjured TA muscles of WT-control and *Gdf15 KO* (*n* = 14 muscles per group). **(B)** Quantification of *Pax7* gene expression (mRNA) using qPCR in WT-control and *Gdf15 KO* muscles at day 4 after CTX injury (*n* = 6 or 7 biological replicates per group). *Pax7* was normalized over *Rpl32*. **(C)** Left: Representative images of IHC detection of laminin (red), PAX7 (green), and nuclei (blue) in WT-control and *Gdf15 KO* at day 4 after CTX injury. Scale bars in the upper left corner represent 100 µm. Right: Number of PAX7^+^ cells in WT-control and *Gdf15 KO* at day 4 after CTX injury (*n* = 12 muscles per group). **(D)** Ki67 (red) and DAPI (blue) immunofluorescence staining shows a robust increase in myoblast proliferation in the presence of rGDF-15 in primary myoblasts. Representative images from untreated and 750 ng/ml rGDF-15–treated myoblasts are shown. Scale bars in the upper left corner represent 100 μm. **(E)** Proliferation index of primary myoblasts in the presence of indicated concentrations of recombinant GDF-15 (*n* = at least 6 independent experiments). **(F)** Immunofluorescence against Desmin (red) and DAPI (blue) shows no difference in myotube formation in the presence of rGDF-15 in primary myoblasts (*n* = 12 independent experiments). Representative images from untreated and 750 ng/ml rGDF-15–treated myoblasts are shown. Scale bars in the upper left corner represent 100 μm. **(G)** Fusion index of primary myoblasts in the presence of various concentrations of recombinant GDF-15 (*n* = at least 6 independent experiments). **(H)** Number of infiltrating CD45^+^ cells in TA muscle of WT mice administered with saline (control) or rGDF-15 (30 µg/kg intramuscularly) at day 2 after CTX injury (*n* = 4 biologically independent samples per treatment group). **(I)** Frequency (in %) of CD45^+^ F4/80^+^ MHCII^+^ MFs from saline (control) and rGDF-15–treated animals at indicated time points following CTX injury (*n* = 5 mice per group). **(J)** Representative flow cytometry 10% quantile contour plots of CD45^+^ F4/80^+^ MHCII^+^ MFs from vehicle (saline) and rGDF-15–treated animals at days 2 and 4 after CTX injury. Images represent four independent experiments with similar results. x and y axis numbers indicate the fluorescence intensity (on the log_10_ scale) of the indicated fluorescent-labeled antibodies for all the plotted events. PB, Pacific Blue; APC, allophycocyanin. **(K)** Frequency (in %) of inflammatory (Ly6C^high^ F4/80^low^) and repair (Ly6C^low^ F4/80^high^) MFs from vehicle (saline) and rGDF-15–treated (30 µg/kg intramuscularly) animals at day 4 following CTX injury (*n* = 5 mice per group). **(L)** Average fiber CSA of regenerating muscle in saline (control) and rGDF-15–treated (30 µg/kg intramuscularly at day 4) animals at day 8 after CTX injury (*n* = 10 per group). In all bar graphs, bars represent mean ± SEM. Exact P values were determined using unpaired Student’s *t* test or ANOVA to compare three or more groups, *, P < 0.05; **, P < 0.01; ***, P < 0.001; ****, P < 0.0001.

As only a few paracrine signaling pathways between MFs and tissue progenitors have been described thus far, we decided to identify the possible effector functions of GDF-15 that might connect muscle-infiltrating repair MFs to the regenerating cell milieu in a paracrine manner. A possible cell target interaction in the regenerating muscle microenvironment is, of course, the muscle progenitor cells. To determine if satellite cells are affected in the absence of GDF-15, we first quantified the number of PAX7^+^ cells in uninjured muscles of adult control and *Gdf15* KO animals through immunohistochemistry (Fig. 4 A). We didn’t detect any significant a priori differences in the numbers of satellite cells at this stage. Next, we measured the mRNA expression of a commonly used marker, *Pax7* (von Maltzahn et al., 2013), via quantitative PCR (qPCR) and quantified the number of PAX7^+^ cells in regenerating *Gdf15 KO* muscles at day 4 after CTX injury. Our data show that *Pax7* mRNA expression is decreased in *Gdf15 KO* muscles compared with controls at day 4 after injury (Fig. 4 B). In agreement with these results, PAX7 staining revealed fewer PAX7^+^ cells in the *Gdf15 KO* muscles at the same time point (Fig. 4 C). These results suggest that the expansion of PAX7^+^ satellite cells upon injury is sensitive to GDF-15 signaling interactions and is likely to be one of the major causes of the muscle regeneration deficiency observed in the *Gdf15 KO* animals. Thus, we hypothesize that GDF-15 may act on satellite cells by affecting their proliferation and differentiation. To assess the effect of GDF-15 on proliferation and fusion, cultured primary myoblasts were treated with recombinant GDF-15 at various doses (Fig. 4, D–G). Using Ki67^+^ staining as a positive indicator of proliferation, or desmin for myotube formation, the addition of 500–750 ng of recombinant (r) GDF-15 to the culture increased myoblast proliferation (Fig. 4, D and E) but had no effect on their differentiation (Fig. 4, F and G), suggesting a regulatory role in activating satellite cell proliferation pathways.

MF-secreted GDF-15 can also have direct effects on the myeloid cell compartment. To assess the potential autocrine function of GDF-15 on the inflammatory component of the regenerating muscle, we injected intramuscularly a single dose of recombinant GDF-15 (30 µg/kg) into CTX-injured muscles of WT mice on day 1 or 3 and accessed the myeloid cell composition at day 2 and 4 after CTX by FACS, respectively. We found that the exogenously added GDF-15 decreased the total number of infiltrating CD45^+^ cells at day 2 after injury (Fig. 4 H), in line with previous observations (Kempf et al., 2011; Zhang et al., 2017), skewed MFs toward expressing higher levels of MHCII molecules both at day 2 and day 4 after CTX (Fig. 4, I and J), and increased the ratio of Ly6C^low^/Ly6C^high^ MFs at day 4 (Fig. 4 K). These data suggest that GDF-15 promotes an accelerated phenotypic transition and can have a positive effect on the maturation and antigen-presenting capacity of these MFs. Last, administration of a single dose of exogenous GDF-15 (administered at day 4 after CTX) in WT mice had a modest but not statistically significant regeneration-enhancing effect in vivo (Fig. 4 L). This finding suggests that (1) the endogenous physiological levels of GDF-15 are sufficient for proper regeneration, and (2) regeneration must be impaired for the rGDF-15 treatment to have any effect. These findings are in line with a previous study on the beneficial role of rGDF-3 in regeneration, where only aged animals with impaired regeneration, but not young animals, benefited from the addition of this growth factor (Patsalos et al., 2018).

Overall, GDF-15 appears to be an effector in regeneration with bivalent and pleiotropic roles in skeletal muscle inflammation/resolution and regeneration.

### GDF-15 is a bona fide transcriptional target of liganded PPARγ and RXR in MFs

**Figure 5.**
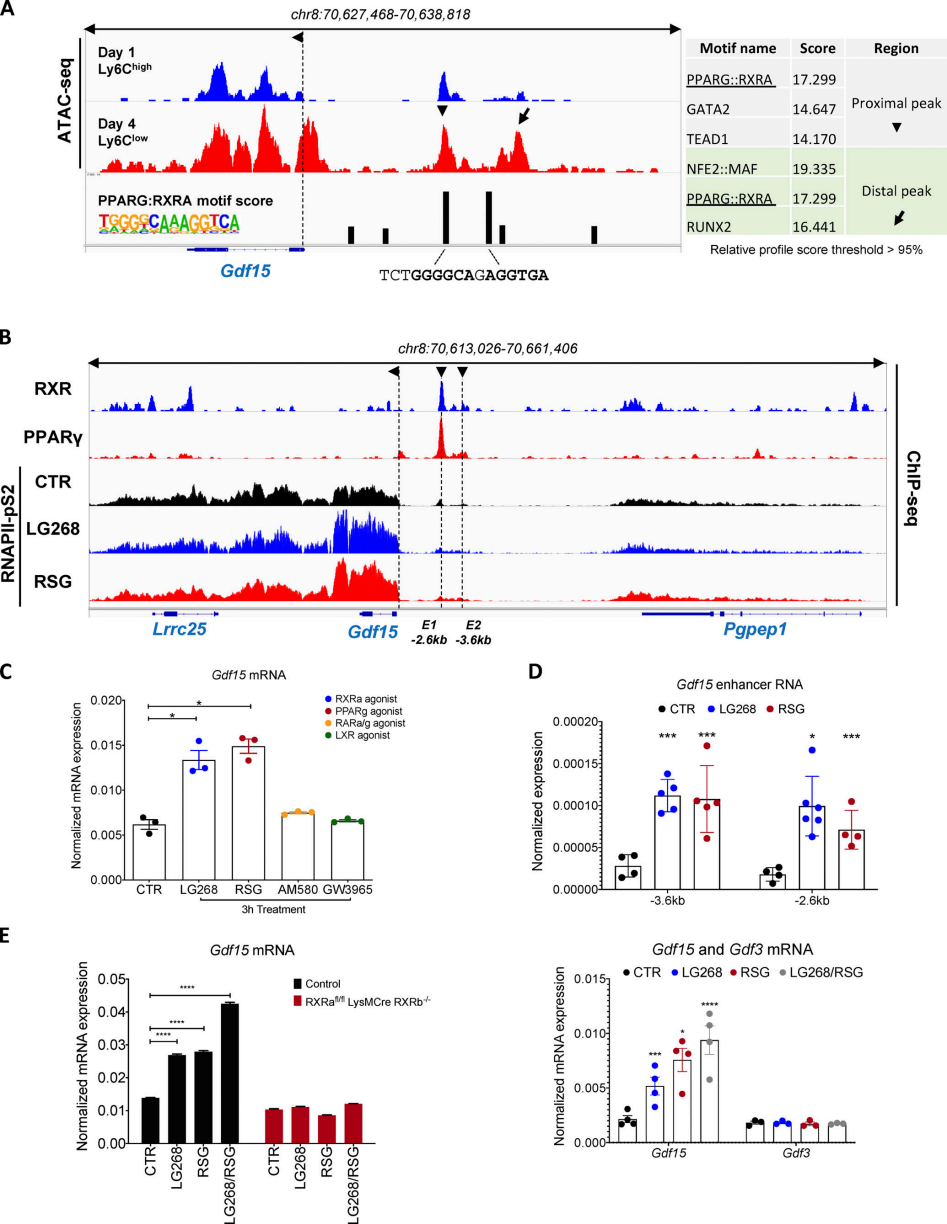
***Gdf15* is a PPARγ and RXRα regulated gene. (A)** Identification of PPARγ and RXRα regulatory elements around the *Gdf15* locus. Genome browser view of ATAC-seq signals from muscle-derived MFs at the indicated genomic region (*Gdf15* locus) showing peak intensities and DR1 predicted motifs scores. De novo motif scores (HOMER) are shown on a table on the right (relative profile score threshold >95%). Arrows indicated the regions upstream of the TSS selected for the in silico motif prediction. The exact motif sequence, highlighting the nuclear receptor (NR) half sites including the 5′ extension, are shown. **(B)** PPARγ and RXRα bind to proximal regulatory elements around the *Gdf15* locus. Genome browser view of the *Gdf15* locus with the indicated ChIP-seq experiments performed in WT BMDMs. ChIP-seq experiments for RNAPII-pS2 were performed in the presence of the indicated nuclear receptor ligands. **(C)** Quantification of *Gdf15* gene expression (mRNA) using qPCR in WT BMDMs treated with the indicated nuclear receptor agonists (*n* = 3 biological replicates per treatment group). **(D)** eRNA measurements of putative enhancer regions around the *Gdf15* locus in WT BMDMs treated with PPARγ or RXR agonists (*n* = at least 4 independent experiments). **(E)** Quantification of *Gdf15* gene expression (mRNA) using qPCR in WT-control and *Rxra*^fl/fl^ *Rxrb*^−/−^LysM-Cre BMDMs treated with indicated ligands individually or in combination (*n* = 3 biological replicates per treatment group). **(F)** Quantification of *Gdf15* and *Gdf3* gene expression (mRNA) using qPCR in WT BMDMs treated with the indicated nuclear receptor agonists individually or in combination (*n* = at least 3 biological replicates per treatment group). Both genes were normalized over *Ppia* (*n* = 3 independent experiments). In all bar graphs, bars represent mean ± SEM. Exact P values were determined using unpaired Student’s *t* test or ANOVA to compare three or more groups. *, P < 0.05; ***, P < 0.001; ****, P < 0.0001.

Next, we decided to pursue the identification of the putative regulatory circuit upstream of *Gdf15*. We have recently described the chromatin accessibility landscape in muscle-infiltrating MFs (Patsalos et al., 2019). We used these Assay for Transposase-Accessible Chromatin using sequencing (ATAC-seq) datasets to gain insights into the regulation of *Gdf15* in muscle-infiltrating MFs. Initially, we analyzed the cistrome around the *Gdf15* locus with the goal to identify distal differentially accessible chromatin regions, which could act as potential enhancers, and then try to predict in silico binding motifs at these sites. We identified two sites located ∼2.6 kb (proximal, E1) and ∼3.6 kb (distal, E2) upstream of the *Gdf15* transcription start site that are changing during the course of regeneration in the muscle-infiltrating MFs. These putative enhancer regions show both differential chromatin accessibility (Fig. 5 A), in line with the gene expression data (Fig. 2 D), and strong DR1 (PPARG:RXRA) binding motifs (Fig. 5 A, right). These motifs are identical, although the distal one is located in a repetitive, lower-complexity region with smaller chromatin openness. Nevertheless, these sequences contain the PPAR-specific 5′ extension that provides minor groove binding, so overall, tighter DNA–protein interactions (Nagy and Nagy, 2020). In addition, based on prior knowledge of expected effects between transcriptional regulators and their target genes stored in the Ingenuity Knowledge Base, we identified PPARγ and RXRα as likely relevant transcriptional regulators of *Gdf15* expression (Fig. S3 A, highlighted in bold). Furthermore, in a previous study (Varga et al., 2016b) of muscle-infiltrating MFs, *Gdf15* showed partial PPARγ dependency as it was among the DE genes in PPARγ-deficient muscle MFs (Fig. S3 F). Based on these findings, we hypothesized that MF PPARγ and its partner, RXRα, target *Gdf15* to establish the repair MF identity and regulate skeletal muscle regeneration.

To determine the molecular mechanisms of how PPARγ and RXRα regulate *Gdf15* expression, we performed chromatin immunoprecipitation sequencing (ChIP-seq) in BM-derived MFs (BMDMs). Consistent with our ATAC-seq data, and the in silico motif analysis, we observed that both RXRα and PPARγ binding occur along the predicted *Gdf15* enhancer regions (Fig. 5 B). In addition, PPARγ and RXRα are nuclear receptors that can sense and interpret fatty acid signals, and thus can be activated by pharmacological targeting. BMDMs were treated for 1 h with LG268 or Rosiglitazone, a potent RXRα- and PPARγ-specific agonist, respectively, and RNA polymerase II–specific ChIP-seq was performed to map the ligand-specific genome changes. We observed a significant increase of RNA pol II binding in the *Gdf15* coding region in response to both agonists, suggesting active transcription (Fig. 5 B). To validate these results at the mRNA level, we proceeded to treat cultured BMDMs with LG268, Rosiglitazone, AM580, and GW2965, the latter two being RARα and LXRβ agonists, respectively, and measured *Gdf15* mRNA levels at 3 h after treatment by qPCR (Fig. 5 C). In response to LG268 and Rosiglitazone, we observed a significant increase of *Gdf15* mRNA, whereas treatment with AM580 and GW3965 resulted in mRNA levels consistent with basal *Gdf15* expression observed in the nontreated control (Fig. 5 C). Furthermore, to confirm whether the predicted enhancer regions are indeed accessible and active upon PPARγ and RXRα ligand treatments, we measured the enhancer RNA (eRNA) expression of these loci in untreated versus LG268- and Rosiglitazone-treated BMDMs (Fig. 5 D). As expected, the enhancer RNAs around the *Gdf15* locus are activated by both ligand treatments (Fig. 5 D). To further expand on these findings, we compared the *Gdf15* mRNA expression between WT and *RXRa*^fl/fl^ LysMCre/*RXRb*^−/−^ BMDMs in response to the same ligands (Fig. 5 E). In the WT-control BMDMs, *Gdf15* mRNA expression is substantially elevated in response to either LG268 or Rosiglitazone treatment, as observed previously (Fig. 5 C), and even more so when treated in tandem (Fig. 5, E and F). In contrast, *Gdf15* mRNA expression in *RXRa*^fl/fl^ LysMCre/*RXRb*^−/−^ BMDMs shows no response to either LG268 or Rosiglitazone treatment (Fig. 5 E). In parallel, *Gdf15* was among the 132 genes that belong to cluster 5 and show RXR dependency (down-regulated) in RNA-seq data from unstimulated RXR-deficient BMDMs (Fig. S3 G). Interestingly, in comparison with a recently discovered myogenic factor (Varga et al., 2016b) with high similarity to GDF-15, namely GDF-3 (it belongs to the same superfamily of growth factors as GDF-15), and the discovery of ligand-independent gene regulation by PPARγ (Daniel et al., 2018), we were interested to explore if these two factors are regulated and behave in a similar fashion. *Gdf3* is expressed and secreted by repair MFs under the control of PPARγ but does not respond to either PPARγ or RXRα ligand treatments (Fig. 5 F), in contrast with *Gdf15*, which responds to both. This ligand-independent regulation of *Gdf3* suggests that both these growth factors may be regulated by the same TFs at the same point in time but with different modes of action (ligand-sensitive versus ligand-insensitive). Taken together, these findings suggest that the *Gdf15* locus has multiple PPARγ:RXR heterodimer-bound active enhancers and that liganded PPARγ and RXR are direct regulators of *Gdf15* expression in MFs and subsequently propose their involvement in the muscle regeneration process.

### Skeletal muscle regeneration is impaired in RXRα/β myeloid-deficient animals

**Figure 6.**
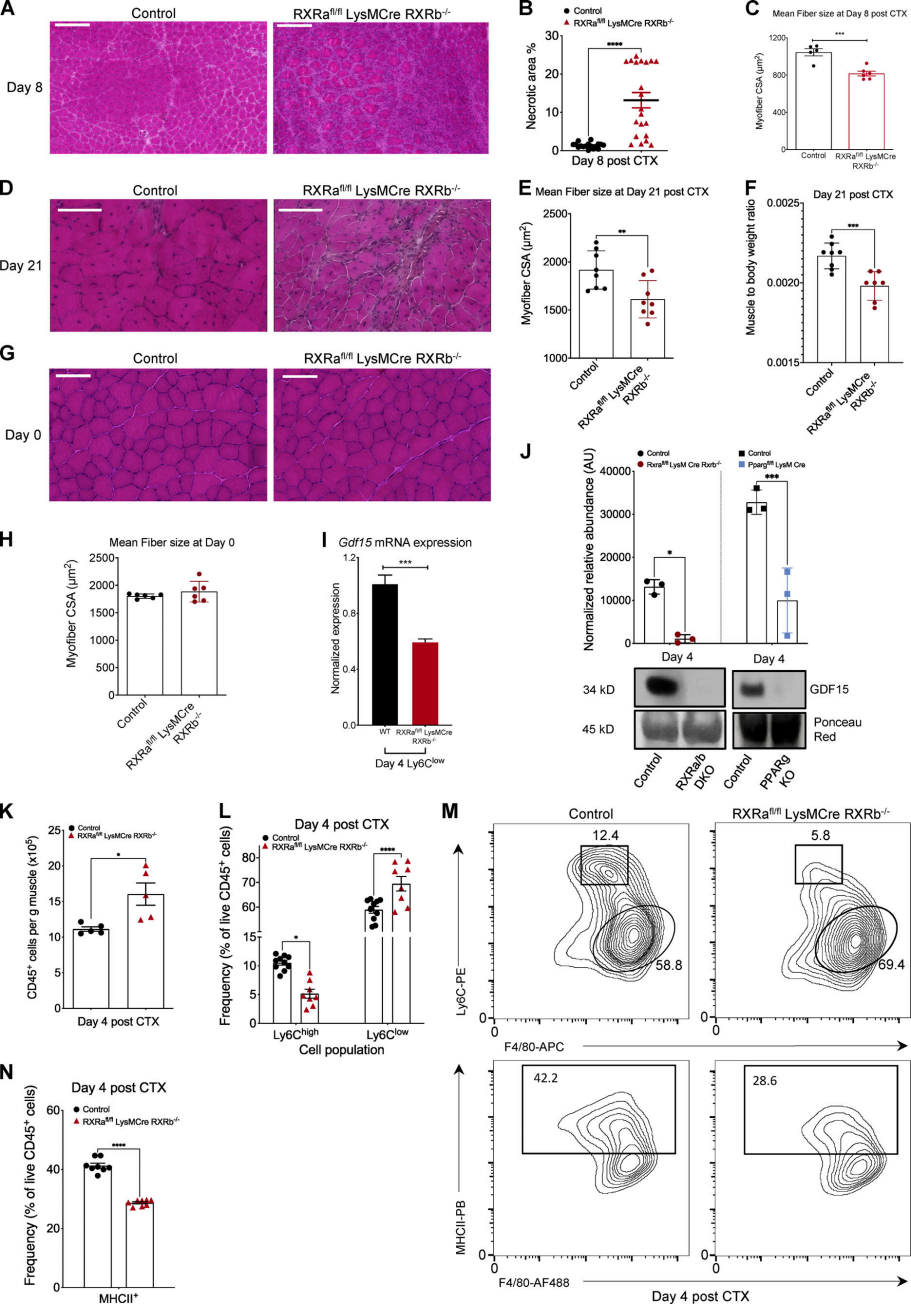
**Skeletal muscle regeneration is impaired in myeloid *RXRα/β*-deficient animals. (A)** Representative images of H&E-stained skeletal muscle (TA) from WT-control and *Rxra*^fl/fl^ *Rxrb*^−/−^LysM-Cre animals at day 8 after CTX-induced injury are shown. Scale bars in the upper left corner represent 100 µm. **(B)** Necrotic fiber area percentage relative to the regeneration area at day 8 after CTX in control (*Rxra*^fl/fl^ *Rxrb*^−/−^) and *Rxra*^fl/fl^ *Rxrb*^−/−^LysM-Cre, muscles (*n* = at least 20 muscles per group). **(C)** Average fiber CSA of regenerating muscle at day 8 after CTX injury in WT-control and *Rxra*^fl/fl^ *Rxrb*^−/−^ LysM-Cre animals at day 8 after CTX injury (*n* = 5 or 6 mice per group). **(D)** Representative images of H&E-stained skeletal muscle (TA) from day 21 post–CTX injury WT-control and *Rxra*^fl/fl^ *Rxrb*^−/−^LysM-Cre animals. Scale bars in the upper left corner represent 100 µm. **(E)** Average fiber CSA at day 21 after CTX injury in muscles from WT-control and *Rxra*^fl/fl^ *Rxrb*^−/−^LysM-Cre animals (*n* = 8 mice per group). **(F)** Muscle to body weight ratio at day 21 after CTX injury in muscles from WT-control and *Rxra*^fl/fl^ *Rxrb*^−/−^LysM-Cre animals (*n* = 7 or 8 muscles per group). **(G)** Representative images of H&E-stained skeletal muscle (TA) from uninjured WT-control and *Rxra*^fl/fl^ *Rxrb*^−/−^LysM-Cre animals. Scale bars in the upper left corner represent 100 µm. **(H)** Average fiber CSA of uninjured muscle WT-control and *Rxra*^fl/fl^ *Rxrb*^−/−^LysM-Cre animals (*n* = 6 mice per group). **(I)** *Gdf15* mRNA expression in WT-control and *Rxra*^fl/fl^ *Rxrb*^−/−^LysM-Cre repair MFs (Ly6C^low^ F4/80^high^) sorted at day 4 after injury. *Gdf15* was normalized over *Ppia* (*n* = 3 independent experiments). **(J)** GDF-15 protein expression in whole-muscle lysates of regenerating muscles from control (respective floxed control littermate), *Rxra*^fl/fl^ *Rxrb*^−/−^LysM-Cre, and *Pparγ*^fl/fl^ LysM-Cre KO mice at day 4 after CTX. Signal quantification is shown in the upper panel. Ponceau Red staining was used for loading control and signal normalization (*n* = 3 mice per group). **(K)** Number of infiltrating myeloid (CD45^+^) cells in regenerating muscle from WT-control and *Rxrα*^fl/fl^ *Rxrβ*^−/−^ LysM-Cre animals at day 4 after CTX injury (*n* = 5 mice per group). **(L)** Percentage of inflammatory (Ly6C^high^ F4/80^low^) and repair (Ly6C^low^ F4/80^high^) MFs from WT-control and *Rxra*^fl/fl^ *Rxrb*^−/−^LysM-Cre muscles at day 4 following CTX injury (*n* = 8 mice per group). To determine P values, two-way ANOVA with multiple comparison test was used. **(M)** Representative flow cytometry 10% quantile contour plots of inflammatory and repair MFs from WT-control and *Rxra*^fl/fl^ *Rxrb*^−/−^LysM-Cre muscles at day 4 after CTX injury. Shapes indicate the gating used for cell frequency quantification (square = Ly6C^high^ inflammatory MFs, circle = Ly6C^low^ repair MFs, rectangle = MHCII^+^ MFs). Representative frequencies for each cell population are shown adjacent or inside each gate. x and y axis numbers indicate the fluorescence intensity (on the log_10_ scale) of the indicated fluorescent-labeled antibodies for all the plotted events. PE, phycoerythrin; PB, Pacific Blue; AF488, Alexa Fluor 488. **(N)** Frequency (in %) of CD45^+^ F4/80^+^ MHCII^+^ MFs from WT-control and *Rxra*^fl/fl^ *Rxrb*^−/−^ LysM-Cre mice at day 4 following CTX injury (*n* = 8 mice per group). In all graphs, bars and lines represent mean ± SEM. Exact P values were determined using unpaired Student’s *t* test unless otherwise noted. *, P < 0.05; **, P < 0.01; ***, P < 0.001; ****, P < 0.0001.

While some aspects of PPARγ’s role in muscle regeneration have been previously demonstrated (Varga et al., 2016b), the role of MF RXR in skeletal muscle injury and regeneration is not known. We hypothesized that MF RXR is a regulator of skeletal muscle regeneration, in part by controlling GDF-15’s expression in repair MFs. This model posits that RXR deficiency in MFs should yield impairment in regeneration. However, the extent and direction of the impairment cannot be predicted given the pleiotropic nature of the role of a TF. To test this hypothesis, we used the double knockout *RXRa*^fl/fl^ LysMCre/*RXRb*^−/−^ mouse strain, which is deficient in *Rxrα* specifically in myeloid lineages and *Rxrb* in all cell types (Kiss et al., 2017). Histological analysis reveals impaired regeneration at day 8 after CTX injury in *RXRa*^fl/fl^ LysMCre/*RXRb*^−/−^ animals versus controls (Fig. 6 A). In addition, at this time point, we observe a significant increase in necrotic fiber content (Fig. 6 B) and a 22% reduction in mean fiber CSA in *RXRa*^fl/fl^ LysMCre/*RXRb*^−/−^ versus control muscles (Fig. 6 C), indicating either a delayed clearance of dying myofibers or altered dynamics of muscle fiber death in the KO animals. Interestingly, this delay in regeneration is still evident at day 21 after injury (Fig. 6 D), as evident by the significant reduction in mean fiber CSA (Fig. 6 E) and muscle mass (Fig. 6 F). At the same time, no developmental impairment was observed in *RXRa*^fl/fl^ LysMCre/*RXRb*^−/−^ uninjured muscles (Fig. 6, G and H), suggesting that the muscle regeneration impairment phenotype in this mouse strain is evident only after injury.

Based on our prior *Gdf15* expression data in BMDMs, we hypothesized that *Gdf15* could be one of the genes dysregulated in the RXRα/β null muscle-MFs. Thus, to assess the impact of RXRα/β deficiency and validate the regulation of *Gdf15* by RXR, specifically in the day 4 repair muscle-MFs, we quantified the *Gdf15* mRNA in FACS-sorted Ly6C^low^ MFs (Fig. 6 I). We observed a significant reduction in *Gdf15* mRNA levels in this day 4 MF subpopulation, which is in accordance with the reduction of GDF-15 protein levels in either RXRα/β or PPARγ MF-specific KO whole muscle lysates (Fig. 6 J). These results prompted us to ask whether the MF infiltration and cellular dynamics have been altered in the RXRα/β double knockout animals upon CTX injury. Indeed, quantification of CD45^+^ cells at day 4 after CTX injury reveals a pronounced increase in the accumulation of myeloid cells in the *RXRa*^fl/fl^ LysMCre/*RXRb*^−/−^ animals (Fig. 6 K). However, analyzing the fractions of MF subpopulations present in these mice via FACS, we observed an increased frequency of Ly6C^low^ F4/80^high^ (Fig. 6, L and M, top) and a lower frequency of MHCII^+^ (Fig. 6 M, bottom, and Fig. 6 N) repair MFs at day 4 after injury. These results suggest that while GDF-15 expression is altered and likely contributes to the observed increased infiltration phenotype, RXRα/β-deficient muscle-MFs have an impaired ability to retain the inflammatory phenotype (most likely due to the cumulative impact of the dysregulation of multiple important genes that are under RXR control), resulting in an inability to clear necrotic fibers, and a premature shift to the Ly6C^low^ repair phenotype. It has been established that if the myeloid cell subpopulation’s orderly transition is impacted in either direction, it will lead to a defect in regeneration, as seen in other models (Patsalos et al., 2019).

### GDF-15 marks a novel repair MF subpopulation with a functionally distinct effector-expressing signature at the single-cell level

**Figure S5.**
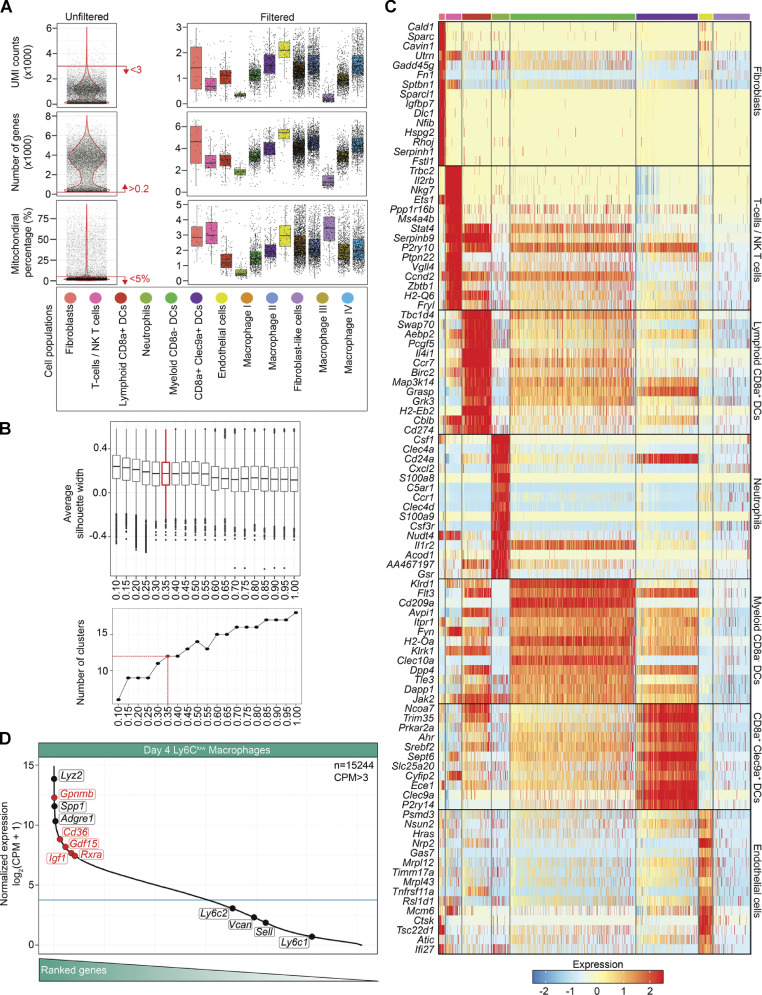
**Technical and quality control measures for scRNA-seq dataset and its analysis (related to** **Fig. 7****). (A)** Violin plots representing the (upper left) number of UMIs, (middle left) genes per cell, and (lower left) mitochondrial gene percentage of all cells before quality control filtering and clustering. Red lines indicate the filtering parameter values. Box plots representing the (upper right) number of UMIs, (middle right) genes per cell, and (lower right) mitochondrial gene percentage per cluster of the cells passing the quality control filtering. The annotated cell populations are color-coded as in Fig. 7 A, and individual cells are shown in black dots. **(B)** Silhouette analysis of SNN clustering resolution parameter. Top: Box-and-whisker plot representing the average silhouette width as a function of SNN resolution parameters. Bottom: Number of clusters identified as a function of SNN resolution parameter. The red box plot and dot correspond to the SNN parameter value (0.35) chosen for cluster annotation and all subsequent analyses. **(C)** Expression heatmap (row Z-score) for top marker genes identified in the eight CD45^+^ non-MF subpopulations at day 4 after CTX classified by SNN. Fibroblast-like cell genes did not pass the unique marker selection criteria (>85% expression in the cells of the cluster and <35% expression in other clusters) to analyze. The columns represent cells and are organized by cell type (color-coded as in A). **(D)** Ranked expression of all genes (*n* = 15,244) in the day 4 Ly6C^low^ RNA-seq dataset in log_2_ CPM reads mapped (CPM >3). This figure illustrates that the expressions of predicted GFEM marker genes (*Igf1*, *Gpnmb*, *Cd36*, *Rxrα*, and *Gdf15*) are among the highest expressed genes and well above the median expression (blue line) of all genes in this repair MF dataset.

**Figure 7.**
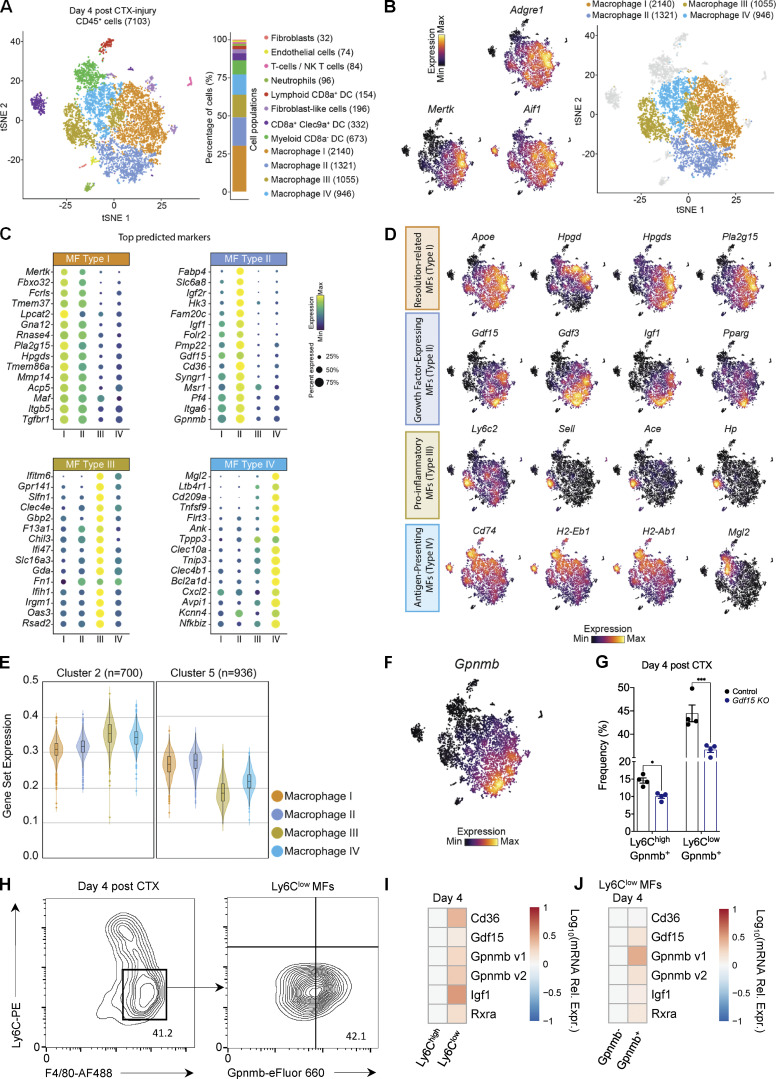
**scRNA-seq analysis of CD45^+^ cells at day 4 after injury reveals the myeloid cell source of GDF-15. (A)** Single-cell transcriptomes derived from CD45^+^ cells isolated from injured TA muscle at day 4 after CTX injury. A total number of 7,103 cells expressing 16,979 different genes were used for the downstream analysis. Data are presented as a t-distributed stochastic neighbor embedding (tSNE) projection to visualize variation in single-cell transcriptomes. Unsupervised SNN clustering resolved at least 12 distinct types of cells (color-coded in legend) and was achieved using a hierarchical tree algorithm (Seurat’s Leiden algorithm). Identification of cell types from SNN clusters was based on cluster-average expression of canonical genes included in the EnrichR Mouse Gene Atlas (Chen et al., 2013; Kuleshov et al., 2016). The composition of cell types presented as a percentage, as well as the absolute number of cells per identified cluster, are shown on the right. The cumulatively largest and most ambiguous group are F4/80^+^ MFs (consisting of 76.9% of the total single-cell transcriptomes). **(B)** Left: Feature plots of classical MF-defining markers (*Adgre1*, *Aif1*, *MerTK*) across all cells (row Z-score). Right: tSNE plot of single-cell transcriptomes representing the clusters of only the F4/80 (Adgre1)^+^ cells. **(C)** Top 15 marker genes for the four identified MF clusters. The dot size represents the percentage of cells within a group with an expression level >0, and color-scale represents the average expression level (row Z-score) across all cells within the cluster (determined by nonparametric Wilcoxon rank-sum test). **(D)** Single-cell expression levels (row Z-score) for selected functional MF markers based on prior literature (Varga et al., 2016a; Varga et al., 2016b; Tidball, 2017; Chazaud, 2020). These markers allowed delineation of four functionally distinct MF subtypes at day 4 after injury. **(E)** Average expression of all genes included in the RPPs of clusters 2 and 5 (defined in Fig. 1 E) in the four MF subtypes identified by scRNA-seq analysis. Cluster 2 genes (700 out of 716) with a stably decreasing expression pattern show a predominance for MF type III (functionally annotated as a pro-inflammatory subtype), while cluster 5 genes (936 out of 952) with a stably increasing expression pattern along the regeneration time course show a predominance for MF type I and II (functionally annotated as resolution-related and growth factor–expressing subtypes, respectively). **(F)** Feature plot of *Gpnmb* expression (row Z-score) defining the MF type II that corresponds to the functionally annotated GFEM subtype. **(G)** Frequency (in %) of CD45^+^ inflammatory (Ly6G^−^ Ly6C^high^ F4/80^low^ GPNMB^+^) and repair (Ly6G^−^ Ly6C^low^ F4/80^high^ GPNMB^+^) MFs from WT-control and *Gdf15* KO mice at day 4 following CTX injury (*n* = 4 mice per group). Bars represent mean ± SEM. Exact P values were determined using unpaired Student’s *t* test. *, P < 0.05; ***, P < 0.001. **(H)** Representative FACS 10% quantile contour plots and gating strategy of CD45^+^ Ly6C^low^ GPNMB^+^ F4/80^+^ MFs at day 4 after CTX in control mice, representing the GFEM subtype. x and y axis numbers indicate the fluorescence intensity (on the log_10_ scale) of the indicated fluorescent-labeled antibodies for all the plotted events. AF488, Alexa Fluor 488. **(I and J)** Heatmaps showing the relative mRNA expression (assessed by qPCR and visualized as log relative expression [Rel. Expr.] values over *Ppia*) pattern of GFEM predicted marker genes in (I) Ly6C^high^ or Ly6C^low^ muscle-infiltrating MFs and (J) FACS-sorted CD45^+^ Ly6C^low^ GPNMB^+^ F4/80^+^ and CD45^+^ Ly6C^low^ GPNMB^−^ F4/80^+^ MFs. mRNA expression values reflect the average of three biological replicates per population. “Gpnmb v1” and “Gpnmb v2” reflect two separate primer sets targeting different exons of the *Gpnmb* gene.

**Figure 8.**
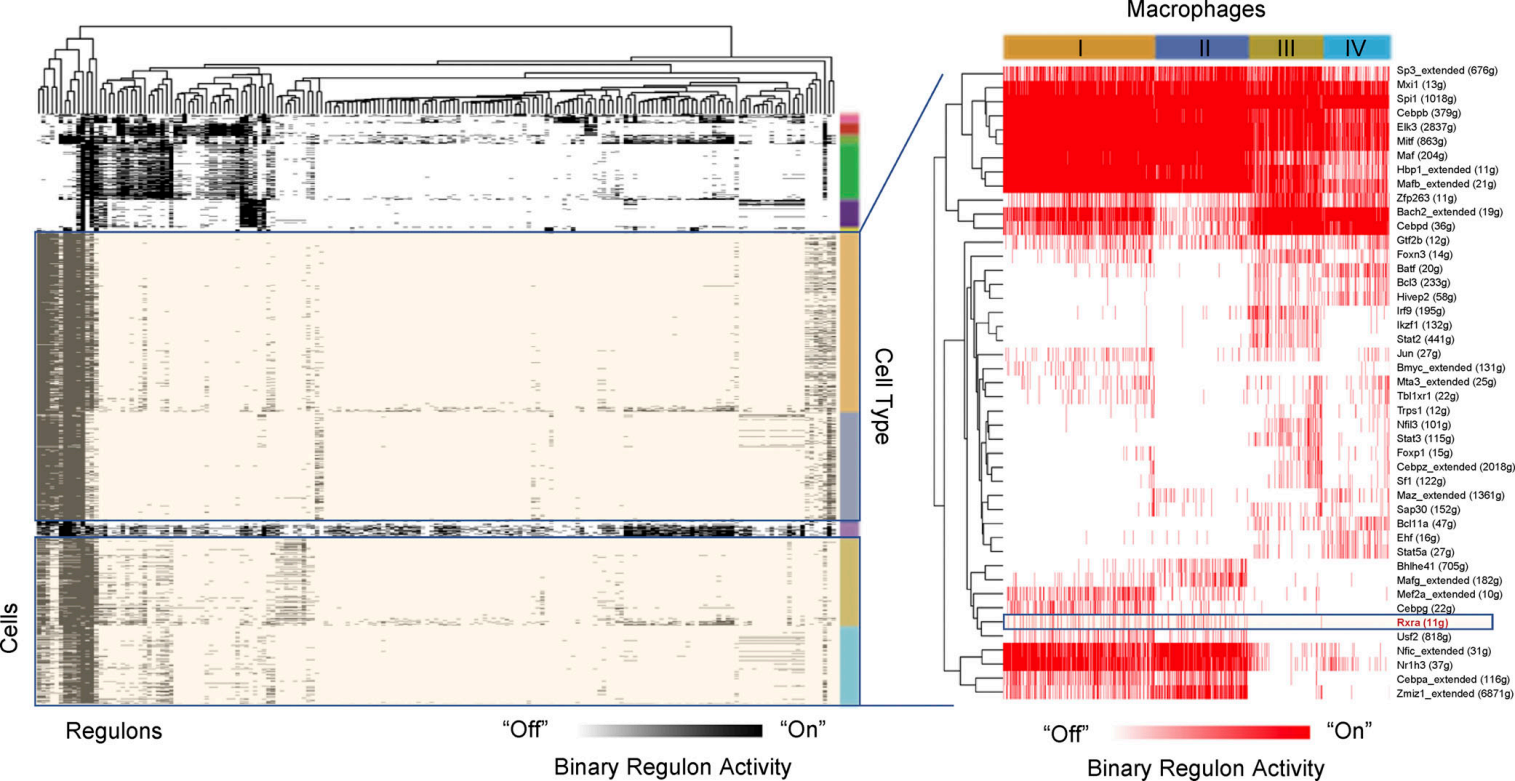
**SCENIC reveals RXR regulon activity in reparatory MFs (GFEM and resolution-related MFs).** Binary activity for each cell is generated from the SCENIC area under curve distribution and plotted as a heatmap, with black (left) or red (right) blocks representing cells that are “on.” For selected MF subtypes (right), the figure depicts regulators. The number of predicted target genes is also given for each regulator. The annotated cell populations are color-coded as in Fig. 7 A. For example, the regulons based on RXRα (highlighted) and Cebpa are active only in MFs associated with growth factor–expressing (type II, dark blue) and anti-inflammatory functions (type I, orange). The known MF regulators Cebpb, Maf, and Spi1 (PU.1) are defining and validating the identity of the entire MF compartment. The cluster of pro-inflammatory MFs (type III, gold) contains a unique set of regulators that include Irf9, Ikzf1, and Stat2. Ehf, Stat5a, and Bcl11a show high activity in MFs with increased antigen-presenting capacity (type IV, light blue).

Due to the large heterogeneity of the regenerating cell milieu (De Micheli et al., 2020), we asked (1) if the source(s) of GDF-15 is (are) all repair MFs or a subpopulation and (2) whether the RPP (involving clusters 2 and 5; Fig. 1, E–H) we observed by profiling sorted monocytes and MFs, marked by genes such as *Gdf15* and *Igf1*, can be assigned to one or multiple groups of cells. To address these questions, we performed droplet-based single-cell 3′ RNA-seq in CD45^+^ cells isolated from CTX-injured tibialis anterior (TA) muscles at day 4. We used the Seurat package for scRNA-seq data filtering and processing (see Materials and methods). Briefly, we removed cells with <200 genes detected, <1,000 unique molecular identifiers (UMIs), or >5% of UMIs mapped to mitochondrial genes (Fig. S5 A, left). Applying these filters eliminated dying cells and doublets presented as outliers with >30,000 UMIs. After filtering, the scRNA-seq dataset contained 7,103 cells, expressing a total of 16,979 different genes (Fig. S5 A, right). We then performed unsupervised shared nearest neighbor (SNN) clustering, which partitioned cells into 12 groups based on their transcriptomic programs after optimizing the SNN resolution parameter by silhouette analysis (Fig. S5 B). Next, we annotated the cell types present in this dataset representing the entire immune cell milieu of the regeneration phase following injury (Fig. 7 A). Identification of cell types from SNN clusters was based on cluster-average expression of canonical genes included in the *EnrichR* Mouse Gene Atlas (Chen et al., 2013; Kuleshov et al., 2016). As expected, the cumulatively largest and most ambiguous group is MFs (76.9% of the total single-cell transcriptomes; Fig. 7 A, right), classified by the expression of known MF markers like F4/80 (*Adgre1*), *Aif1*, and *Mertk* (Fig. 7 B). To further discriminate the ambiguous populations, we also performed differential gene expression analysis between cells within each group and all other cells in the dataset (Fig. 7 C and Fig. S5 C). Both analyses revealed four different subtypes of MFs with varying cell number composition and unique gene expression profiles (Fig. 7, A–C). We labeled them as types I, II, III, and IV and focused our analysis on these four cell populations (Fig. 7 B). Next, we asked (1) what markers define these distinct MF subtypes, (2) whether we can draw conclusions on the potential function of these four different states of MFs based on their unique gene expression patterns, and (3) whether *Gdf15* has any distinct expression pattern within these MF populations. Interestingly, *Gdf15* was predicted unbiasedly as one of the specific markers for the type II MFs (Fig. 7 C). Significantly, the majority of this repair MF subset is positive for *Pparγ* and *Rxrα* but also for the majority of other known secreted growth factors, including *Igf1* and *Gdf3* (Fig. 7 D). Type I MFs, the largest MF group, are defined by high expression of MF maturation markers like *Mertk*, but also seem to exclusively express several enzymes involved in the production of pro-resolving lipid mediators like *Hpgd*, *Hpgds*, and *Pla2g15*, as well as *Apoe* and *Tgfbr1*, characteristic of the M2-like anti-inflammatory phenotype (Baitsch et al., 2011; Ho et al., 2016; Ho et al., 2017; Giannakis et al., 2019), and thus most likely involved in the resolution phase of the regenerative response (Fig. 7, C and D). Type III MFs, the smallest group, seem to be the remaining pro-inflammatory monocytes/MFs with high expression of inflammatory monocyte markers *Ly6c2*, *Sell*, *Ace*, and *Hp*, while type IV MFs seem to have higher antigen-presenting capacity characterized by expression of classical MHCII proteins, such as Cd74 and the H2 family, as well as C-type lectins like *Mgl2* (Fig. 7 D; Denda-Nagai et al., 2010; Panduro et al., 2018). Interestingly, we also found that 98.08% of the genes expressed in the RPPs of clusters 2 and 5 (defined in Fig. 1 E) are detected in the four MF subtypes identified by scRNA-seq analysis. More specifically, cluster 2 genes (700 out of 716, 97.7%) with a steadily decreasing expression pattern show a predominance for MF type III (functionally annotated as a pro-inflammatory subtype), while cluster 5 genes (936 out of 952, 98.3%) with a steadily increasing expression pattern along the regeneration time course show a predominance for type I (functionally annotated as resolution-related MFs) and II MFs (functionally annotated as the GFEM subtype; Fig. 7 E). In a complementary analysis, we applied the single-cell regulatory network inference and clustering (SCENIC) workflow on our dataset (Aibar et al., 2017; Van de Sande et al., 2020). This analysis provides insight into the transcriptional regulators that define the identity of cell types constituting the regenerating cell milieu (Fig. 8). Binarization of the AUCell scores (see Materials and methods) for the predicted regulators and subsequent clustering of the cell-regulon matrix reveals clusters of regulators characteristic of each MF subtype and thus potential mechanisms driving cellular heterogeneity (Fig. 8). The prediction of cell states is based on the shared activity of regulatory subnetworks. For example, the regulators Cebpb, Maf, and Spi1 (PU.1) are defining the identity of the entire MF compartment, thus validating the predictions of the SCENIC workflow (Fig. 8). Significantly, regulons based on RXRα and Cebpa are active only in MFs associated with growth factor–expressing (type II, dark blue) and anti-inflammatory functions (type I, orange). In addition, the cluster of pro-inflammatory MFs (type III, gold) contains a unique set of regulators that includes Irf9, Ikzf1, and Stat2, while Ehf, Stat5a, and Bcl11a show high activity in MFs with increased antigen-presenting capacity (type IV, light blue). This analysis predicts RXR unbiasedly as a direct regulator of the GFEM subtype and further implicates this pathway and subsequently its targets as a defining regulatory network during regeneration. In summary, by testing the hypothesis that GDF-15 can predict the core effector repair signature at the single-cell level, we identified *Gdf15*-expressing immune cells as a novel and distinct repair MF subpopulation of the myeloid-driven regeneration cellular ecosystem, hereby termed GFEMs. In addition, the SCENIC workflow predicts RXRa as one of the core regulators (and the only signal-dependent TF) of the GFEM transcriptional program.

As an additional proof of concept and to validate these results at the protein level, we decided to test whether a predicted cell surface marker (based on the scRNA-seq dataset) could distinguish the GFEMs from the rest of the MF subtypes by FACS. We chose a top predicted cell surface marker protein called GPNMB (also known in the literature as osteoactivin) for further analysis (Fig. 7 F). Notably, *Gpnmb* was also predicted as a top DE gene with effector functions during the day 4 Ly6C^low^ MF versus blood monocytes comparison (Fig. 2, A and B) and is ranked among the top 10 genes with the highest counts per million (CPM) values overall in the entire day 4 Ly6C^low^ RNA-seq dataset (Fig. S5 D). It also belongs to cluster 5 following *Gdf15* and *Igf1* gene dynamics (Fig. 1, E and H; Fig. 2 E; and Fig. S5 D). FACS analysis at this time point on *Gdf15* KO mice using the cell surface expression of GPNMB as an additional marker of MF subtype specification suggests an impairment in the formation of the GFEM population (Fig. 7 G). These findings also suggest that GPNMB expression can accurately predict and thus validate the presence and abundance of type II MFs (GFEMs) detected in the scRNA-seq data (∼18.5%) when gated for its cell surface protein expression on CD45^+^ Ly6C^low^ F4/80^high^ repair MFs at day 4 after CTX (Fig. 7 H). To further validate that GPNMB is a bona fide marker of type II MFs (GFEMs), we FACS-sorted CD45^+^ Ly6C^low^ F4/80^high^ Gpnmb^+^ and CD45^+^ Ly6C^low^ F4/80^high^ Gpnmb^−^ repair MFs at day 4 after CTX (Fig. 7 H) and quantified the expression of predicted GFEM markers (Fig. 7 C) such as *Cd36*, *Gdf15*, *Gpnmb*, *Igf1*, and *Rxrα* by qPCR (Fig. 7, I and J). Notably, all these predicted markers are expressed well above the median expression (based on CPM values) of any gene in the day 4 Ly6C^low^ repair MF RNA-seq dataset (Fig. S5 D). Collectively, these results validated the existence of the GPNMB^+^ Ly6C^low^ F4/80^high^ repair MF subpopulations.

In summary, our data show (1) that the PPARγ–RXR–GDF-15 axis is a novel and essential component of MF-mediated skeletal muscle repair by acting locally in a paracrine and autocrine manner, and (2) that GDF-15 marks and is exclusively expressed by a novel and functionally distinct MF subtype, GFEM, within the regenerating cell milieu.

## Discussion

The immune system is emerging as a critical regulator of many physiological processes, including skeletal muscle regeneration. Although several distinct and isolated immune-mediated mechanisms have been described in recent studies (Serrano et al., 2008; Ruffell et al., 2009; Perdiguero et al., 2011; Deng et al., 2012; Mounier et al., 2013; Tonkin et al., 2015; Corna et al., 2016; Varga et al., 2016b; Patsalos et al., 2017; Patsalos et al., 2019; reviewed recently by Juban, 2021), the full-fledged dynamic aspects of the regenerative immunity as manifested in MF phenotype specification, as well as the potential existence of a specific RPP, remain elusive. In this study, (1) we performed a comprehensive bulk transcriptomic analysis of inflammatory monocytes and derived MF subpopulations; (2) we identified two clusters of genes with steadily and continuously increasing/decreasing expression levels from circulating monocytes culminating in repair promoting MFs, suggesting that signaling events drive the repair phenotype without evidence of transitions between pro-inflammatory and pro-regenerative phenotypes; (3) as a component of it, we have uncovered the PPARγ–RXR–GDF-15 axis as a novel and essential component of MF-mediated skeletal muscle repair by acting locally in a paracrine and autocrine manner; and (4) by using scRNA-seq, we validated that *Gdf15* along with other regeneration-promoting factors mark and are predominantly expressed by a novel and functionally distinct GFEM subtype within the regeneration-associated myeloid cell population, as the product of the RPP.

Dynamically changing MF phenotypes as the result of plasticity are a leading paradigm in innate immunology. They explain the phenotypic transition from circulating monocytes to inflammatory Ly6C^high^ and then to repair Ly6C^low^ MFs, a process highly correlated with the tissue regeneration kinetics. These transitions are accompanied by a dynamic crosstalk between MFs and other muscle tissue components driven by a transcriptional reprogramming process. To our knowledge, this is the first time circulating monocyte profiling, and multiple time points using the most inclusive MF gating strategy, are taken into consideration simultaneously for a more comprehensive analysis of the MF phenotypic and functional state continuum. There is also strong evidence that the indicated time points we selected reflect all phases of regeneration (pro-inflammatory, resolution, and repair phases) and at the same time provide sufficient cell numbers to perform the RNA-seq immune profiling (Giannakis et al., 2019; Patsalos et al., 2021). The aforementioned switches in MF phenotype have been documented by multiple transcriptomic, epigenomic, lineage tracing, and lipidomic approaches by several laboratories, including ours (Perdiguero et al., 2011; Mounier et al., 2013; Varga et al., 2013; Wang et al., 2014; Tonkin et al., 2015; Varga et al., 2016a; Panduro et al., 2018; Giannakis et al., 2019; Patsalos et al., 2019). These findings collectively suggest that the successive immune cell states from circulating inflammatory monocytes via inflammatory MFs to repair type MFs can be interpreted as a hierarchical continuum of cell states (Novak and Koh, 2013). However, it remains to be resolved how effectors, regulatory factors, and surface markers define this monocytic/MF continuum and where cells isolated from acute sterile muscle injuries fall in this spectrum. It was also an open question if there are additional functionally distinct cellular subtypes. Our work goes a considerable distance toward answering these questions. We used bulk RNA-seq to take advantage of its superior sensitivity and robustness to detect low copy number mRNA species (i.e., TFs) compared with single-cell approaches on sorted circulating monocytes and muscle-infiltrating MFs isolated from a comprehensive set of time points after CTX injury. We detected transcriptional programs with transiently or steadily changing dynamics. The transient nature of some of the changes (such as the several thousands of genes represented in clusters 1, 3, 4, 6, and 7) is not very surprising because it represents switches from unstimulated to inflammatory states and back to the opposing repair or anti-inflammatory/resolution state. However, the existence of clusters 2 and 5 representing >1,600 protein-coding genes steadily up- or down-regulated over this continuum of cellular states and over the time course is not compatible with a simple switch forward and then backward model of gene expression. This intriguing finding prompted us to ask the following questions: (1) What are the effectors that participate in the steadily changing RPP transcriptional programs? (2) How do these effectors contribute overall to the MF phenotype switch during muscle regeneration? And (3) how are they regulated at the transcriptional level? Using the expression pattern of well-established repair growth factor *Igf1* as a benchmark or guide, we narrowed down our studies to the MF-secreted growth factor GDF-15, using it as a proof-of-concept that could act as an effector of myoblast and MF activity, similar to IGF-1, and would initiate a sequence of events as part of a regulatory axis with PPARγ and RXRα. The timing and localization of GDF-15 in the CTX injury model firmly suggested that GDF-15 is a general, MF-specific regulator of muscle regeneration.

To our knowledge, only seven other gene deletion paradigms, DUSP1, AMPKa1, BACH1-HMOX1, Metrnl-STAT3, NFIX, C/EBPβ–IL-10 or IGF1 deficiency in muscle-infiltrative MFs, were reported to lead to altered MF phenotype switch (Ruffell et al., 2009; Perdiguero et al., 2011; Mounier et al., 2013; Tonkin et al., 2015; Patsalos et al., 2019; Baht et al., 2020; Saclier et al., 2020; Welc et al., 2020). However, the molecular mechanism of the MF phenotypic switch mediated by GDF-15 remains to be fully elucidated. Based on our findings, GDF-15 expression can be potentially used as a marker of the phenotype switch from inflammatory to repair MFs, reflecting a functional difference in growth factor secretion and antigen-presenting status between the two distinct repair MF subtypes. In addition, our in vitro results with primary myoblasts suggested the presence of a regulatory circuit between MFs and muscle cells. Indeed, exogenous GDF-15 appeared to be an especially robust enhancer of myoblast proliferation, while local administration of rGDF-15 in vivo leads to a subtle but significant increase in the antigen-presenting capacity of repair MFs. From a physiological perspective, the MHCII increase could also either signify the terminal maturation of repair MFs or confer protection from a potential subsequent infection (Jin et al., 2018). Circulating Ly6C^high^ monocytes that enter the tissue during inflammation or injury differentiate into repair and anti-inflammatory MFs or pro-inflammatory and immune-stimulatory DCs (Geissmann et al., 2010). They express CD11c and CD11b and exhibit some antigen-presenting activity, although much less than CD103^+^ DCs (Stables et al., 2011; Ramachandran et al., 2012; Rivollier et al., 2012; Zigmond et al., 2012). Thus, the ability of GDF-15 to impact functional features and potentially the differentiation of monocytes or DCs has important ramifications on immunity and tissue homeostasis. In this context, it will be important to uncover receptors and pathways that enable and mediate the activity of GDF-15 within distinct cellular compartments after its secretion. In addition, as other cell types are also involved in the regeneration process (Joe et al., 2010; Uezumi et al., 2010; Heredia et al., 2013), it cannot be excluded that GDF-15 has effects on other cell types such as fibro/adipogenic progenitors (Hidestrand et al., 2008; Joe et al., 2010; Lemos et al., 2015). However, a key component of the signaling, the receptor(s) of GDF-15, remains elusive. Given the wide associations of GDF-15 with a variety of biological processes, including pregnancy, metabolism, and inflammation, it is very likely that GDF-15 plays additional roles to those described in our studies (Tsai et al., 2018; Patel et al., 2019; Breit et al., 2021) and may act on multiple different low(er) affinity receptors on different cell types and in concert with other bone morphogenic proteins or TGF-β family members, as has been demonstrated and postulated for these proteins (Antebi et al., 2017). Some of these receptors, like Tmed1, are expressed in cells present during muscle regeneration (McKellar et al., 2020 *Preprint*). It is also possible that its high-affinity receptor, GFRAL (Mullican et al., 2017; Yang et al., 2017), may be expressed on other rare cell types outside of the area postrema in the brain, or more likely, additional receptors for GDF-15 may exist but have not yet been discovered. Identifying these receptors and the potential interaction with other TGF-β family members would further increase our understanding of the roles of GDF-15 in physiology and pathology and potentially allow the identification of novel therapeutic targets for regenerative immunotherapy.

The family of GDFs, like GDF-3 and GDF-15, are secreted effectors with pleiotropic functions in different tissues and organs. However, they are among the few growth-promoting factors released locally by muscle-infiltrating inflammatory cells to trigger and control the distinct actions of satellite cells throughout the myogenic process. GDF signaling has been previously associated with stimulation of hypertrophic muscle growth and myogenesis by regulating the proliferative and differentiation capacity of muscle stem cells. Overall, they have been characterized as regulators of muscle development, homeostasis, and regeneration (Varga et al., 2016b; Kleinert et al., 2018; Gil et al., 2019; Assadi et al., 2020; Borner et al., 2020; Breit et al., 2021; Laurens et al., 2020). Paradoxically, global, potentially maladaptive actions for GDF-15 have also been proposed, such as promotion of atrophy, malaise, and muscle wasting (Johnen et al., 2007; Tsai et al., 2018; Patel et al., 2019). Such a dichotomy is not uncommon with cytokines and growth factors, molecules that are, by definition, pleiotropic (i.e., IL-6 and IGF-1). For example, IL-6, principally defined as a proinflammatory cytokine in the circulation, is also one of the few genuine myokines produced by and/or acting on skeletal muscle (Hirano, 1998; Muñoz-Cánoves et al., 2013). In regenerating muscle, IL-6 produced locally by various cell types, including infiltrating MFs, has a positive impact on the proliferative capacity of muscle stem cells, similar to GDF-15 (Serrano et al., 2008). This local physiological mechanism functions to provide sufficient muscle progenitors under circumstances that require a high number of these cells, such as following injury. These positive effects are typically associated with their transient production and short-term action. On the contrary, persistent inflammatory conditions and other chronic disease states (i.e., cancer) are associated with elevated systemic levels that are long-lasting. In such situations, the actions of these molecules are coupled with increased muscle wasting, very often acting in combination with other molecules or functioning indirectly to promote atrophy. Elevated levels of circulating IL-6 are believed to be mediating the tumor cachexia phenotype, including muscle wasting (Strassmann et al., 1992; Zhang et al., 2009). Thus, our findings uncovered a local mode of action for GDF-15 consistent for being both an endocrine and paracrine growth factor, similar to IL-6 and IGF-1. Circulating IGF-1 is mainly produced by the liver and acts as the primary mediator of growth hormone–dependent growth, as an important mitogenic factor regulating growth, nutrient metabolism, reproduction, and aging, while local IGF-1 is produced by peripheral tissues acting as a paracrine/autocrine factor for local tissue growth (Stuard et al., 2020).

The nature and biological significance of GDF-15 in muscle regeneration are further supported by the two lines of our molecular investigations. First, regarding the upstream regulators, from the perspective of muscle regeneration, we consider the most important finding to be the identification of GDF-15 as a regeneration factor, which is subject to robust regulation by PPARγ and RXRα in all relevant MF subtypes. To ascertain that GDF-15 is indeed a direct transcriptional target, we analyzed an extensive range of genomic and epigenomic data. GDF-15 is expressed in an RXRα/PPARγ-dependent fashion and can be induced by specific RXRα/PPARγ synthetic ligands in BMDMs but does not belong to the group of canonical PPARγ-regulated genes (such as *Angptl4* or *Fabp4*) described in earlier myeloid cell–related studies (Welch et al., 2003; Szanto et al., 2010). This level of detail goes much beyond what has been known regarding ligand regulation of GDF-15 in unrelated cell types and in silico predictions (Baek et al., 2004; Suzuki et al., 2006; Araki et al., 2009; Yu et al., 2010; Hofer et al., 2018). In parallel, recent reports suggested cell metabolism as a defining factor in MF identity and functional status (Vats et al., 2006; Odegaard and Chawla, 2011; Lavin et al., 2014; Okabe and Medzhitov, 2014). PPARγ and RXRα are metabolic sensors and regulators controlling several effector genes implicated in MF polarization (Daniel et al., 2018) and muscle regeneration (Varga et al., 2016b). Thus, the role of the RXR signaling pathway is intriguing and goes beyond this gene alone. It covers a network of genes as identified using the SCENIC approach. We have previously reported that GDF-3 is dependent on the presence of PPARγ (Varga et al., 2016a). Moreover, several other genes from clusters 2 and 5 are likely to be subject to regulation by these nuclear receptors. This raises the intriguing possibility that this signaling pathway is one of the drivers of the repair phenotype and thus of the RPP. This also implies that lack of RXR in MFs is likely to have a broader effect on regeneration, which is the sum of all the altered gene expression events and not necessarily a phenocopy of GDF-15 deficiency. Second, regarding the cell type selectivity of GDF-15 expression, our results indicate that repair MFs are the predominant, if not the only, source of GDF-15 within the injured tissue. To validate this finding, we used expression profiling at the single-cell level to fully resolve the heterogeneity and cellular complexity and further understand the different functions of each MF subset. This effectively complemented and extended our bulk RNA-seq analyses. Our scRNA-seq suggests that these effectors are expressed simultaneously and in a stage-specific manner within GFEM. Their expression is specific and highest in this cell population but not selective or exclusive.

Recent studies presented transcriptomic atlases of regenerating muscle, focusing on satellite and progenitor cells from homeostatic and toxin-injured muscles (Dell’Orso et al., 2019; De Micheli et al., 2020; Oprescu et al., 2020). Here, we present an annotated and comprehensive single-cell transcriptomic immune dataset of the regeneration phase following injury with over 7,000 single-cell transcriptomes with an average of 3,808 expressed genes per cell, adding to the growing repository of scRNA-seq datasets in skeletal muscle regeneration and complementing, but not replacing, the bulk RNA-seq data presented above. Our scRNA-seq analyses confirm prior consensus regarding the immune cell populations involved in the temporal response to muscle injury and provide a deeper annotation of additional immune cell types, subpopulations, and states with higher resolution, compared with prior scRNA-seq studies (12 different cell types via SNN clustering), including four novel and likely functionally distinct MF subtypes. In our unbiased analysis, we followed an elaborate workflow to identify the number of clusters and marker genes based on multiple bioinformatic packages, including but not limited to the most widely used, named *Seurat* by the Satija laboratory (Butler et al., 2018). However, many of these clustering workflows rely on user-tuned parameter values that need to get tailored to each dataset, which is one of the major computational limitations in the analysis of single-cell datasets. To address this issue, we took an independent approach to determine cluster resolution by using a subsampling-based approach (*chooseR*) that was recently published and simultaneously guides parameter selection while characterizing cluster robustness (Patterson-Cross et al., 2021). In addition, we applied manual marker selection as well as automated cell annotation pipelines (*SingleR*) that perform unbiased cell type recognition by leveraging reference transcriptomic datasets of pure cell types to infer the cell of origin of each single cell independent of clustering, ensuring that we do not overcluster our cell types and subtypes (Aran et al., 2019). These unbiased analyses firmly pointed to the existence of four MF clusters at day 4 after CTX. We found that GDF-15, and other growth factors like IGF-1 and GDF-3, are highly expressed in a distinct repair-MF subpopulation we termed growth factor–expressing macrophages (GFEMs). Furthermore, we characterized this repair-MF subset by FACS using a predicted and highly enriched cell surface molecule (GPNMB), which is again a specific but not exclusive marker of this subset. Future studies could use functional as well as a cytometry by time of flight dataset composed of several markers to provide an orthogonal validation of MF subtypes and their surface receptor expression variability during the time course of regeneration. Trajectory analysis could also allow parsing the MF differentiation and subtype specification after injury in distinct states like anti-inflammatory/resolution–related, growth factor–secreting, pro-inflammatory, and antigen-presenting, with diverse gene expression signatures, as recently hypothesized (Patsalos et al., 2021).

The mechanistic role of GDF-15 in regulating myoblast signal transduction in the context of regeneration remains poorly understood. Several reports suggest that GDFs may serve as ligands that interplay with numerous ligand-receptor systems involved in myogenic cell fate regulatory pathways (Heldin and Moustakas, 2016). Future studies will take advantage of the available technology that allows the selective interference with GDF-15 production, GDF-15 receptors, and downstream signaling in specific cell types at a desired experimental stage to fully decipher the contribution of GDF-15 in different contexts. This knowledge will also potentially allow selective interference of the deleterious actions of GDF-15 in pathological contexts and promotion of the beneficial effects of GDF-15 for therapeutic purposes. There are several additional questions raised by our study. What other effects are induced by GDF-15, and how do they affect acute and chronic regenerative inflammation outcomes? What are the regulatory factors that coordinate the production of key growth factors? Finally, the therapeutic applicability of this pathway is yet to be determined.

Taken together, PPARγ/RXRα–controlled GDF-15 induction in MFs appears to be an exploitable therapeutic approach for regeneration immunotherapy, immunomodulation, and regulation of acute exercise-induced stress responses (Gil et al., 2019). Our findings also have implications for pathological processes in which recurrent muscle damage and asynchrony in repair due to genetic conditions lead to debilitating, degenerative muscle diseases, such as Duchenne muscular dystrophy. Therefore, it will be of great importance to determine if GDF-15 is also a regulator of muscle regeneration in Duchenne muscular dystrophy or other types of myopathies, which are most of the time associated with the permanent presence of inflammatory cells, and especially MFs.

## Materials and methods

### Ethical approval

All animal experiments were performed in accordance with ethical regulations and approved by the Institutional Animal Care and Use Committees (IACUCs) at Johns Hopkins University (license no. MO18C251). Animals were handled according to our animal facility’s regulatory standards at Johns Hopkins All Children’s Hospital, managed by Charles River Laboratories.

### Mice

WT 8-wk-old BoyJ (B6.SJL-Ptprca Pepcb /BoyJ, stock #002014) and C57BL/6J (stock #000664) control mice were obtained from The Jackson Laboratory and bred under specific pathogen–free (SPF) conditions. Mice were housed five per cage, kept on a 12-h light cycle (6 a.m. to 6 p.m.) in an SPF vivarium that conforms to IACUC and Association for Assessment and Accreditation of Laboratory Animal Care International specifications. *Gdf15* KO mice were obtained from Dr. Se-Jin Lee at Johns Hopkins University School of Medicine (Baltimore, MD), *Rxra*^fl/fl^ *Rxrb*^−/−^LysM-Cre mice were obtained from Pierre Chambon (Institut de Génétique et de Biologie Moléculaire et Cellulaire, Strasbourg, France), and mice carrying floxed alleles of *Pparγ* (Pparγ^fl/fl^ LysM-Cre) were created as described previously (Szanto et al., 2010). All irradiation experiments were performed under anesthesia in cohorts of 12 animals per experiment as previously described (Patsalos et al., 2017; Giannakis et al., 2019; Patsalos et al., 2019). Briefly, mice were anesthetized with a single intraperitoneal dose of ketamine/xylazine (ketamine 80–100 mg/kg and xylazine 10–12.5 mg/kg). Irradiated and BM-transplanted mice were maintained in SPF status (autoclaved top filter cages) for the entire course of experimentation. Antibiotics (amoxicillin antibiotic and clavulanic acid [500 mg/125 mg/liter of drinking water]) were administered in the drinking water for 4 wk after transplantation to minimize bacterial contamination within the water source and potentially decrease the burden of gastrointestinal bacteria. Irradiated mice were also fed autoclaved rodent chow ad libitum. Animals that undergo irradiation for BMT typically lose a considerable amount of weight, only to gain it back relatively quickly after successful transplantation. At our institutions, weight loss of 20% or greater was used as a rationale for euthanasia before the intended experimental end point according to the IACUC guidelines. When necessary and for tissue collection, mice were euthanized by either isoflurane overdose (adjusted flow rate or concentration to 5% or greater) or CO_2_ exposure (adjusted flow rate 3 liter/min) in accordance with Johns Hopkins University’s IACUC guidelines. When indicated, recombinant GDF-15 (30 µg/kg) was administrated intramuscularly under anesthesia.

### Acute sterile muscle injury

Mice (8–12-wk-old males) were anesthetized with isoflurane (adjusted flow rate or concentration to 1.5%), and 50 µl of 10 µM CTX (217503-1MG; EMD Millipore) was injected in the TA muscle. Mice were brought out of anesthesia and monitored until they were euthanized and processed at various time points. Muscles were recovered for flow cytometry analysis at day 1 to day 4 after injury or for muscle histology at day 8 to day 21 after injury.

### Histological analysis of muscle regeneration

Muscles were removed, mounted on precut cork discs (63305; EMS) using tragacanth gum (104792; MP Biomedicals), and snap-frozen in nitrogen-chilled isopentane (−160°C). 8-µm-thick cryosections were cut and stained with H&E. For each histological analysis, at least five slides (per condition) were selected where the total regenerative region within the CTX-injured TA muscle was at least 70%. For each TA, myofibers in the entire injured area were counted and measured. H&E-stained muscle sections were scanned with the Mirax or Leica Aperio High-Definition digital slide scanner. The CSA and necrosis (expressed as a percentage of the total number of myofibers) were quantified with HALO software (Indica Labs). CSAs for these samples are reported in square micrometers. Areas of necrosis were identified based on the following histological criteria: blurring of cell borders, cytoplasmic fragmentation, caliber variation, cell distances, loss of nuclei, and increased immune cell infiltration (Al-Sawaf et al., 2014). Necrotic/phagocyted myofibers were further defined as pink, pale, patchy fibers invaded by basophil cells (MFs). The necrotic fiber content data presented here were quantified using both immunohistochemistry (Desmin staining) and histology.

### BMT

Recipient congenic BoyJ mice (8 wk old) were irradiated with 11 Gy using an X-rad 320 (Precision X-ray Irradiation Systems) x-ray unit for the ablation of the recipient BM. During irradiation, one of the hindlimbs was shielded as described previously (Patsalos et al., 2017). Following the irradiation, isolated BM cells (in sterile RPMI-1640 medium) were flushed out the femur; tibia and humerus from donor C57Bl/6J mice were transplanted into the recipient mice by retro-orbital injection (20 × 10^6^ BM cells per mouse). This experimental BMT CD45 congenic model allows us to detect donor, competitor, and host contribution in hematopoiesis and repopulation efficiency of donor cells (congenic mice with CD45.1 versus CD45.2). The CD45.1 and CD45.2 contributions were then detected by flow cytometry, usually 8–12 wk following the BMT. In short, a cut at the tail tip of the mice provided a drop of blood that was placed into 0.5 ml PBS + 1% FBS + 10 U/ml heparin buffer (samples kept on ice). The cells were directly stained by mouse anti-mouse CD45.2-FITC (clone 104) and rat anti-mouse GR1-PE (clone RB6-8C5) antibodies (BD PharMingen; 1/50 dilution) and incubated on ice for 30 min. After two washes with ice-cold PBS/FBS/heparin buffer, we resuspended the cells in 0.5–1 ml FACS Lysing solution (BD Cat #349202). We incubated for 5 min at room temperature and then centrifuged the cells (400 *g*, 5 min, 4°C). We ran the double-stained samples on FACS (MoFlo Astrios, Cytoflex) and determined the ratio of donor cells. The repopulation (blood chimerism) is usually >90% gated on either the granulocyte or monocyte fraction, as described previously (Patsalos et al., 2017).

### In vivo muscle force measurement

In vivo twitch and tetanic forces were measured as described previously (Giannakis et al., 2019). Briefly, animals were first anesthetized with 3% vaporized isoflurane mixed with O_2_ and then positioned under a heat lamp to maintain the body temperature at 37°C. Fur was removed from hindlimbs using fine electric hair clippers (Wahl). The right hindlimb was restrained at the knee firmly with a clamp (secured to a fixed steel post), and the foot was strapped to a footplate/force transducer with a dual motor-arm attached (Aurora Scientific) to prevent movement from the contraction of other muscle groups. Electrical stimulations were applied across two 30-G needle platinum electrodes placed through the skin just below the knee and beneath the TA muscle to stimulate the tibial nerve. In all measurements, we used 0.1-ms pulses at a predetermined supramaximal stimulation voltage. TA muscles were stimulated with a single 0.1-ms pulse for twitch force measurements and a train of 150 Hz for 0.3-s pulses for tetanic force measurements. A 2-min rest was given to the animal while under anesthesia to allow muscles to return to normal function after tetanus. We performed five twitch and then five tetanic measurements on each muscle, with 2–3 min of recovery between each measurement. For these measurements, we used the 610A Dynamic Muscle Control (DMC) software from Aurora Scientific.

### In vivo grip strength

The grip strength meter (Harvard Apparatus) allows the study of neuromuscular functions in rodents by determining the maximum force displayed by an animal. In this context, grip strength changes are interpreted as evidence of motor neurotoxicity or impairments in muscle development. The procedure was performed as described previously by the International Mouse Phenotyping Consortium. Briefly, the grip strength meter is positioned horizontally, and the animals are held by the tail and lowered toward the apparatus. The animals are allowed to grasp the metal grid with their hindlimbs and are then pulled backward in the horizontal plane. The force applied to the grid just before it loses grip is recorded as the peak tension. This force was measured in grams. Data are visualized on the control unit display and exported for analysis. Five consecutive grip strength measurements for each mouse were performed with 1 min rest between measurements.

### Immunohistochemistry

Tissue sections were fixed and permeabilized in ice-cold acetone for 5 min and blocked for 30 min at 20°C (room temperature) in PBS containing 5% BSA. Tissues were stained for 1 h at room temperature using a primary antibody diluted in 2% BSA. For PAX7 staining, antigen epitope retrieval was performed as described previously (Feng et al., 2018). The primary antibodies used for immunofluorescence were rabbit anti-laminin (Sigma-Aldrich; L9393) at a dilution of 1/200, mouse anti-PAX7 (DSHB) at a dilution of 1/20, rabbit anti-Desmin (Abcam; 32362) at a dilution of 1/200, and rat anti-F4/80 (Abcam; 6640) at a dilution of 1/200. In all cases, the primary antibody was detected using secondary antibodies (dilution 1/200) conjugated to FITC (JIR 703–095-155) or Cy3 (JIR 711–165-152). The nuclei were counterstained with 0.1–1 µg/ml Hoechst. Fluorescent microscopy was performed using either a Carl Zeiss Axio Imager Z2 microscope or a Nikon Eclipse Ti2 inverted microscope equipped with lasers at 488, 568, and 633 nm. Images were analyzed and assembled using Fiji and Illustrator CS5 (Adobe).

### In vivo isolation of myeloid cells from muscle

Isolation of muscle-infiltrating MFs was performed as described previously (Giannakis et al., 2019; Patsalos et al., 2019). Briefly, the fascia of the TA was removed, and muscles were dissociated in either RPMI containing 0.2% collagenase B (Roche Diagnostics GmbH) at 37°C for 1 h or by using the magnetic-activated cell sorting (MACS) Skeletal Muscle Dissociation Kit (130–098-305; Miltenyi) or gentleMACS Octo Dissociator, per kit instructions. Cell homogenate was filtered through a 100-µm and a 40-µm filter, and CD45^+^ cells were isolated using magnetic sorting (Miltenyi Biotec). For FACS, MFs were treated with Fcγ receptor blocking antibodies, 5% normal rat serum, and 5% normal mouse serum, then stained with a combination of PE-conjugated anti-Ly6C antibody (eBioscience; HK1.4), APC-conjugated or FITC-conjugated anti-F4/80 antibody (eBioscience; BM8), FITC-conjugated anti-Ly6G antibody (BioLegend; 1A8), Pacific Blue–conjugated anti-MHCII antibody (BioLegend; M5/114.15.2), and eFluor660-conjugated anti-GPNMB antibody (eBioscience; CTSREVL). Ly6C^high^ F4/80^low^ MFs, Ly6C^low^ F4/80^high^ MFs, and Ly6G^high^ Ly6C^med^ F4/80^−^ neutrophils were quantified. Gating strategy is shown in Fig. S1, B–D. For DC and NK cells quantification, APC-conjugated anti-NK1.1 (BioLegend; PK136), and PE-conjugated anti-CD11c (BD PharMingen; HL3) antibodies were also used. In each experiment, compared samples were processed in parallel to minimize experimental variation. Cells were analyzed on a Cytoflex S (Beckman Coulter), BD FACSAria III, or MoFlo Astrios EQ (Beckman Coulter) sorter, and data analysis was performed using BD FACSDIVA and FlowJo V10 software.

### Isolation and purification of mouse peripheral blood monocytes

The monocyte purification procedure was performed as described previously (Houthuys et al., 2010) with slight modifications. In short, the blood from C57BL/6 mice was taken by cardiac puncture to maximize the amount of blood obtained in an endotoxin-free manner. Up to 5.3 × 10^7^ ± 4 × 10^6^ white blood cells (viability >96%) were obtained from 15 ml of blood from 15 animals after red blood cell lysis (*n* = 3). To minimize cell aggregation and adhesion to plastic, all purifications were performed at 0–4°C in PBS/BSA/EDTA (MACS buffer). For red blood cell lysis, cells were resuspended in 0.5–1 ml FACS Lysing solution (BD; Cat #349202), incubated for 5 min at room temperature, and then centrifuged (400 *g*, 5 min, 4°C). For FACS and sorting, MFs were treated with Fcγ receptor blocking antibodies, 5% normal rat serum, and 5% normal mouse serum. They were then stained with a combination of APC-conjugated anti-Ly6C antibody (eBioscience; HK1.4), PE/Cy7-conjugated CD11b antibody (BD Bioscience; M1/70), PE-conjugated Ly6G antibody (BioLegend; 1A8), and PerCP/Cy5.5-conjugated MHCII antibody (BioLegend; M5/114.15.2). Gating and sorting strategy are shown in Fig. S1 A.

### Differentiation of BMDMs and ligand treatments

Isolation and differentiation were completed as described earlier (Daniel et al., 2014). Briefly, BMDMs were obtained from BM precursor cells. Total BM was obtained from mice by flushing femurs and tibiae BM with DMEM. Cells were cultured in DMEM medium containing 20% FBS and 30% conditioned medium of L929 cell line (enriched in CSF-1) for 6 d. MFs were seeded at 50,000 cells/cm^2^ for all experiments. Media were changed to serum-free MF media (Gibco; #12065074), and MFs were activated with vehicle (DMSO), 0.1 μM LG268, 1 μM rosiglitazone, 1 μM AM580, 1 μM GW3965, or in combination in DMEM containing 20% FBS medium for 3 h.

### Primary myoblast culture and in vitro effects of GDF-15 on myogenesis

Murine myoblasts were obtained from TA muscle and cultured using standard conditions in DMEM/F12 (Gibco Life Technologies) containing 20% FBS and 2% Ultroser G (Pall Inc.). Briefly, young mice’s TA muscles were opened and cleared of nerves/blood vessels/fascia. Muscle preparations were lightly digested with collagenase, and the resulting cells were plated, then serially expanded. For proliferation studies, myoblasts were seeded at 10,000 cells/cm^2^ on Matrigel (1/10 dilution) and incubated for 1 d with 2.5% FBS medium containing GDF-15 mouse recombinant protein (250–750 ng/ml). Cells were then fixed with 4% PFA, incubated with anti-Ki67 antibodies (Abcam; #15580; 1/400 dilution) for 1 h at room temperature, and subsequently visualized using Cy3-conjugated secondary antibodies (Jackson ImmunoResearch Inc.; 1/200 dilution). For differentiation studies, myoblasts were seeded at 30,000 cells/cm^2^ on Matrigel (1/10) and incubated for 3 d in a medium with 2% horse serum and mouse recombinant GDF-15 protein (250–750 ng/ml). Cells were then incubated with anti-desmin antibodies (Abcam; #32362; 1/200 dilution) in combination with a Cy3-conjugated secondary antibody (Jackson ImmunoResearch Inc). The nuclei were counterstained with 0.1–1 µg/ml Hoechst. Myogenic cell fusion (calculated as the number of nuclei within myotubes divided by the total number of nuclei) was evaluated as described earlier (Saclier et al., 2013). Fluorescent microscopy was performed using a Carl Zeiss Axio Imager Z2 microscope equipped with lasers at 488, 568, and 633 nm. Images were analyzed for proliferation and fusion index using Fiji.

### SDS-PAGE and Western Blot

GDF-15 protein expression was measured using Western blot analysis. Homogenates were prepared from frozen CTX-injected TA muscles using a TissueLyser II (QIAGEN) and stainless-steel beads in RIPA buffer (Abcam), with a protease and phosphatase inhibitor cocktail (Thermo Fisher Scientific), or from primary CD45-selected cells from CTX-injured TA muscle. Samples were run on Bolt 4–12% Bis-Tris Plus (Invitrogen; NW04127BOX) and subsequently transferred onto 0.45-µm polyvinylidene fluoride membranes via a Mini Blot Wet Transfer Module (Thermo Fisher Scientific; NW2000) for 1 h at 20 V. Membranes were blocked with Odyssey Blocking Buffer with 0.1% Tween 20 for 1 h at room temperature, and GDF-15 was targeted using a rabbit polyclonal anti–GDF-15 primary antibody (Abcam; ab105738) at 1:1,000 dilution in Odyssey Blocking Buffer with 0.1% Tween 20 overnight at 4°C. Secondary antibody goat anti-rabbit IgG-HRP (Santa Cruz; sc-2030; 1/10,000) was then used for 1 h at room temperature. Total protein was measured using REVERT total protein stain (LI-COR) or Ponceau S solution 0.2% (SERVA; 33427.01). Bands were visualized using an Odyssey Digital Infrared Imaging System (LI-COR) or a BioRad ChemiDoc MP Imaging System and quantified using Odyssey Application Software version 3.0 (LI-COR) or BioRad Image Lab Software (v6.1), respectively. For monomer detection, membranes were visualized using SuperSignal West Pico Luminol Substrate (Thermo Fisher Scientific; 34080) for 5 min and processed using a Kodak X-Ray Film Developer. Images were quantified using ImageJ software.

### Recombinant GDF-15 production

For the production of the in-house GDF-15 protein, the mature peptide of GDF-15 was cloned into a pET20b(+) plasmid and produced in *Escherichia coli*. Recombinant mature GDF-15 was His-tag purified, underwent endotoxin removal, was lyophilized, and then was freeze-dried.

### RNA isolation

Total RNA was isolated with TRIZOL reagent according to the manufacturer’s recommendations (Zymo Research; Direct-zol RNA MiniPrep Plus). 20 µg glycogen (Ambion) was added as a carrier for RNA precipitation.

### Real time qPCR and enhancer RNA measurements

Transcript quantification was performed by real-time RT-qPCR using SYBR Green assays. RT-qPCR results were analyzed with the standard delta Ct method, and results were normalized to the expression of *Ppia* or *Rpl32*. For eRNA measurements, RNA was DNase-treated and reverse-transcribed with the High-Capacity cDNA Reverse Transcription Kit (Applied Biosystems) according to the manufacturer’s protocol. Enhancer transcript quantification was performed by qPCR reactions using SYBR green master mix (BioRad), and eRNA levels were normalized to *Ppia*. mRNA and eRNA primer sequences and locus coordinates are provided in Table 1.

**Table 1.** Primers for qRT-PCR detection of eRNA and mRNA

Gene	Forward primer, 5′**–**3′	Reverse primer, 5′**–**3′	Genomic coordinates
*Gdf15* eRNA −2.6 kb	TAG​GAT​CCC​ACT​TCG​CCA​GG	TTA​ACC​CCC​AAG​TGA​CAC​CC	chr8: 70633546–70633793
*Gdf15* eRNA −3.6 kb	GAC​ATC​TCC​CCT​CGG​GTT​CTA	CAC​TAC​ACC​ACA​GCA​CCA​GC	chr8: 70634857–70634974
*Gdf15* mRNA	GCT​GTC​CGG​ATA​CTC​AGT​CC	CTT​CAG​GGG​CCT​AGT​GAT​GTC	-
*Gdf3* mRNA	GGGTGTTCGTGGGAACCT	CCA​TCT​TGG​AAA​GGT​TTC​TGT​G	-
*Ppia* mRNA	GCG​TCT​CCT​TCG​AGC​TGT​TT	ACC​ACC​CTG​GCA​CAT​GAA​TC	-
*Myh2* mRNA	TCC​AAG​TTC​CGC​AAG​ATC​CA	GCG​CAT​GAC​CAA​AGG​TTT​CA	-
*Pax7* mRNA	GGC​ACA​GAG​GAC​CAA​GCT​C	GCACGCCGGTTACTGAAC	-
*Rpl32* mRNA	ACA​TTT​GCC​CTG​AAT​GTG​GT	ATC​CTC​TTG​CCC​TGA​TCC​TT	-
*Igf1* mRNA	TGG​ATG​CTC​TTC​AGT​TCG​TG	GCA​ACA​CTC​ATC​CAC​AAT​GC	-
*Cd36* mRNA	TTG​TAC​CTA​TAC​TGT​GGC​TAA​ATG​AGA	CTT​GTG​TTT​TGA​ACA​TTT​CTG​CTT	-
*Rxrα* mRNA	ACA​TTT​CCT​GCC​GCT​CGA​CTT	TGA​TGA​CAG​AGA​AGG​GCG​GA	-
*Gpnmb v1* mRNA	ACGGCAGGTGGAAGGACT	CGG​TGA​GTC​ACT​GGT​CAG​G	-
*Gpnmb v2* mRNA	AGC​CAA​TAG​GAA​ACT​GCC​CC	AAC​AAC​AGT​TCC​CAG​CCA​CA	-

### RNA-seq library preparation

cDNA libraries for RNA-seq were generated from 100–400 ng total RNA using the TruSeq RNA Sample Preparation Kit (Illumina) or NEBNext Ultra II RNA Library Prep Kit (Illumina), according to the manufacturer’s protocol. Briefly, poly-A tailed RNA molecules were pulled down with poly-T oligo attached magnetic beads. Following purification, mRNA was fragmented with divalent cations at 85°C, and cDNA was generated by random primers and SuperScript II enzyme (Life Technologies). Second strand synthesis was performed, followed by end repair, single "A" base addition, and ligation of barcode-indexed adaptors to the DNA fragments. Adapter specific PCRs were performed to generate sequencing libraries. Libraries were size-selected with E-Gel EX 2% agarose gels (Life Technologies) and purified by the QIAquick Gel Extraction Kit (QIAGEN). Libraries were sequenced on either a HiSeq 2500 or a NextSeq 550 instrument using the NextSeq500/550 High Output Kit v2.5. At least three biological replicates were sequenced for each sorted population.

### Gene expression data processing and analysis

RNA-seq samples of the sorted and isolated blood monocytes, Ly6C^high^, and Ly6C^low^ MFs of days 1, 2, and 4 after CTX were analyzed in parallel using the *nf-core/rnaseq* v3.2 pipeline (Ewels et al., 2020). Briefly, raw single-end reads were quality-checked by FastQC (https://github.com/s-andrews/FastQC) and aligned to the *mm10* (GRCm38) genome assembly with STAR using default parameters (Dobin et al., 2013). Genes were quantified using Salmon (Patro et al., 2017). Normalized coverage density tracks (bigwig files) for RNA-seq data were generated by *deeptools* and *bamCoverage* (Ramírez et al., 2016). Genes with CPM <10 were filtered out, and only protein-coding genes were kept for downstream analysis. Statistically significant difference was considered false discovery rate (FDR) <0.05 from GLM test using R package *edgeR* (Robinson et al., 2010). We assessed the overall relationship of the datasets by using multidimensional scaling on the normalized values and hierarchical clustering on the distance measures of Spearman correlation values and visualized in R. For *k-means* clustering, we calculated the optimal cluster number by evaluating the sum of squared error between increasing number of clusters (elbow-plot on Fig. S2 C) along with gap statistics (Fig. S2 C, inset). Next, we applied the *kmeans* function on the scaled data using seven centers. For Pearson similarity metric analysis and to find genes that follow a similar trend and rate to *Igf1*, the “Find Similar Entities” feature of the Strand NGS 3.4 software was used, with similarity cutoff set at ≥0.9. Heatmaps were generated based on scaled log_2_-transformed CPM values using the *pheatmap* R package.

### GO and GSEA

GSEA was performed using *hypeR* (Federico and Monti, 2020). We used the hallmark gene sets of MSigDB (Subramanian et al., 2005; Liberzon et al., 2011; Liberzon et al., 2015) keeping significantly enriched terms with P < 0.05. Lists of genes were also analyzed using the Panther tool (http://www.geneontology.org/), REACTOME (https://reactome.org/), and the GO pathway databases for GSEA. GOs with P values <0.05 were selected (Fisher’s exact test with FDR correction), and results were presented according to their −log_10_ P value.

### IPA Upstream Regulator Analysis

To explain the biological activities of each cluster, we identified the upstream transcriptional regulators in each module with a P value of overlap <0.05 using the IPA Upstream Regulator Analysis (QIAGEN; https://www.qiagenbioinformatics.com/products/ingenuity-pathway-analysis). Regulators with at least 20 known gene targets in the analyzed dataset were chosen for further analysis.

### scRNA-seq

After tissue digestion and bead selection, CD45^+^ single-cell–sorted suspensions were washed and resuspended in 0.04% BSA in PBS at a concentration of at least 400 cells/µl. Cells were counted manually with a hemocytometer to determine their concentration. scRNA-seq libraries were then prepared using the Chromium Single-Cell 3′ reagent kit v3.1 (10X Genomics) in accordance with the manufacturer’s protocol. Briefly, the cells were diluted into the Chromium Single-Cell A Chip to yield recovery of ∼10,000 single-cell transcriptomes with <5% doublet rate. Following the library preparation, the libraries were sequenced on the NovaSeq 6000 sequencer (Illumina) to produce ∼450 million reads per library and, on average, a minimum of 40,000 reads per single cell.

### scRNA-seq data analysis

scRNA-seq reads were processed and aligned to the mouse reference transcriptome (mm10) with the Cell Ranger version 3.1.0 (10x Genomics). We used CellBender to eliminate technical artifacts. From the gene expression matrix, the downstream analysis was performed with R version 4.0.2 (2020–06-22). Quality control, filtering, data clustering and visualization, and the differential expression analysis were performed using *Seurat* (v3.2.2) R package (Butler et al., 2018) with some custom modifications to the standard pipeline. Genes expressed in less than three cells and cells with <1,000 UMIs and <200 genes were removed from the gene expression matrix. In addition, we removed any single cell with >5% UMIs mapped to mitochondrial genes, as well as obvious outliers in the number of UMIs (cell doublets; Fig. S1, A and B). After log-normalizing the data, the expression of each gene was scaled, regressing out the number of UMIs and the percent mitochondrial gene expressed in each cell. We performed principal component analysis on the gene expression matrix and used the first 30 principal components for clustering and visualization. Unsupervised SNN clustering was performed with a resolution of 0.35, and visualization was done using t-distributed stochastic neighbor embedding (Becht et al., 2018). We performed a silhouette analysis (R *cluster* package) to select an optimal SNN resolution parameter that balanced the number of expected clusters (given known marker expression) with a maximal average silhouette width. Finally, differential expression analysis was achieved using Seurat’s *FindAllMarkers* function using a likelihood ratio test that assumes the data follow a negative binomial distribution and only considering genes with >log_2_(0.25) fold-change and expressed in at least 40% of cells in the cluster. Feature plots were generated using the *Nebulosa* package (Alquicira-Hernandez and Powell, 2020 *Preprint*). In the SCENIC workflow, coexpression modules between TFs and candidate target genes are first inferred using GENIE3 (Aibar et al., 2017; Van de Sande et al., 2020). RcisTarget then identifies modules for which the regulator’s binding motif is significantly enriched across the target genes and creates regulons with only direct targets. AUCell uses the area under the curve (AUC) to score the activity of each regulon in each cell, thereby yielding a binarized activity matrix.

### Mapping, normalization, and analysis of ATAC-seq

The primary analysis of ATAC-seq has been performed using the newest version of ChIP-seq analysis command-line pipeline (Patsalos et al., 2019; Daniel et al., 2018) including the following steps: alignment to the mm10 mouse genome assembly was done by the Burrows-Wheeler Aligner tool (Li and Durbin, 2009), and binary alignment map files were created by *SAMTools* (Li et al., 2009). Signals (peaks) were predicted by *MACS2* (Zhang et al., 2008). Artifacts were removed according to the blacklist from the Encyclopedia of DNA Elements (ENCODE; ENCODE Project Consortium, 2012) and filtered for further analysis by removing low mapping quality reads (mapping quality score <10), duplicated reads, and reads located in blacklisted regions. All regions derived from at least any two samples were united within 0.5 kb, and those summits having the highest MACS2 peak score in any sample were assigned to each region. Promoter-distal regions were selected by excluding the transcription start site ± 0.5 kb regions according to the mouse GRCm38.p1 (*mm10*) annotation version. In total, we identified 57,409 peaks from muscle-derived MF samples. Tag directories used by HOMER (Hypergeometric Optimization of Motif EnRichment) in the following steps were generated with a 120-nucleotide fragment length with *makeTagDirectory* (Heinz et al., 2010). Genome coverage (bedgraph and tdf) files were generated by makeUCSCfile.pl (HOMER) and *igvtools*, respectively, and used for visualization with IGV2 (Thorvaldsdóttir et al., 2013). Coverage values were further normalized by the upper decile value detected in the consensus regions for each sample to minimize the inter-sample variance.

### Differential chromatin accessibility analysis

To identify the open chromatin regions involved in muscle-derived MF differentiation, we compared the two end point cell populations of this process: day 1 Ly6C^high^ versus day 4 Ly6C^low^. *DiffBind* v2.6.6 was used to identify differentially opened regions, with *DESeq2* (method = DBA_DESEQ2, bFullLibrarySize = FALSE; Love et al., 2014). An ATAC-seq region was defined as differentially changed if the peak showed |log_2_ FC| >1.5 and FDR-corrected P value <0.05.

### Motif analysis

De novo motif analysis of differentially opened chromatin regions was performed using HOMER’s *findMotifsGenome.pl* (*-len 12 -size 200 -dumpFasta -bits -fdr*; Heinz et al., 2010). Motif matrices of HOMER’s collection selected by the resulting top de novo motifs were used to calculate motif enrichments using HOMER’s annotatePeaks.pl program and plotted in R (Heinz et al., 2010). Motif logos were created with *seqLogo* in R.

### ChIP-seq analysis

The primary analysis of ChIP-seq-derived raw sequence reads has been performed similarly as described for the ATAC-seq analysis. Peaks were predicted by *MACS2*, and artifacts were removed by *BEDTools* according to the blacklist of ENCODE. Motif enrichment analyses of the ±50-bp vicinity of the highest RXR peak summits (up to 1,000) were performed by findMotifsGenome.pl using *-mask*, *-len 10,12,14,16*, *-bits*, *-preparse*, and *-homer2* parameters (HOMER). Three RXR, PPARγ, and RNA polymerase II-pS2 ChIP-seq replicates derived from differentiated BMDMs were analyzed by *DiffBind* v1.0.9 (consensus peak set was formed from those peaks predicted from at least two of six samples). RNAPII-pS2 abundance on gene bodies (using *mm10* RefSeq annotation) was calculated and tested using package *Rsubread* and *edgeR* (P ≤ 0.05 and FC ≥ 1.5), respectively.

### Statistics

ANOVA with Bonferroni correction for multiple testing was used to determine statistical significance. Adjusted P values are stated within the figure legends. All experiments were performed using at least three independent experiments from distinct samples. No repeated measures were performed. For RT-qPCR analyses, at least three biological samples were used for each condition. For FACS marker analysis, at least four independent samples were analyzed, and at least 5 × 10^5^ cells were counted for each FACS cell population. For the histology experiments, at least 10 biological samples were used (each animal provides two biological samples). For the CSA distribution, two-way ANOVA was used to mark significance for each size class. In scatter dot plots, mean and SEM are shown in addition to individual data points. In bar graphs, bars show the mean of the indicated number of samples, and error bars represent SEM. Student’s *t* tests and ANOVA analyses were performed in GraphPad Prism 8 (GraphPad Software) with 95% CIs, and P < 0.05 was considered statistically significant (*, P < 0.05; **, P < 0.01; ***, P < 0.001; ****, P < 0.0001).

### Online supplemental material

Fig. S1 shows the sorting/gating strategy for the circulating monocytes and muscle-infiltrating MFs. Fig. S2 shows the hierarchical and *k-means* clustering of the muscle-infiltrating MF expression dynamics along with gene enrichment analysis and coexpression modules for each cluster. Fig. S3 shows the GO pathway and upstream regulator analysis of DE genes between blood monocytes and muscle-infiltrating repair MFs and the overlap with RXR- and PPARγ-regulated genes. Fig. S4 shows that GDF-15 ablation allows normal muscle development and muscle growth in uninjured animals but impacts the cellular composition of the injured milieu. Fig. S5 provides technical and quality control measures for the scRNA-seq dataset and its downstream analysis. Table S1 provides the gene expression changes between MF subsets. Table S2 provides the genes with cluster indication of the *k-means* clustering analysis and membership score.

## Supplementary Material

Table S1 shows that gene expression changes between MF subsets corresponding to the maturation from circulating monocytes to Ly6C^high^ inflammatory MFs, between day 1 and day 4 transition from Ly6C^high^ inflammatory to Ly6C^low^ repair MFs, and between the two ends of the differentiation spectrum (blood monocytes versus day 4 repair MFs).

Table S2 shows gene lists with cluster indication of the *k-means* clustering analysis and membership score (indicates how closely they correlate/match with the cluster core) that revealed the dynamically changing transcriptomic profile of immune cell subsets after CTX injury (related to Fig. 1 E).

## Data Availability

The RNA-seq data presented in this article have been deposited in GEO under accession nos. GSE182455 and GSE164722. The scRNA-seq data have been deposited in GEO under accession no. GSE161467. The ATAC-seq data analyzed in this article have been deposited in GEO under accession no. GSE129393. The ChIP-seq data are available in GEO Superseries accession no. GSE110465 and GEO Subseries accession no. GSE107456.

## References

[bib1] Aibar, S., C.B. González-Blas, T. Moerman, V.A. Huynh-Thu, H. Imrichova, G. Hulselmans, F. Rambow, J.C. Marine, P. Geurts, J. Aerts, . 2017. SCENIC: single-cell regulatory network inference and clustering. Nat. Methods. 14:1083–1086. 10.1038/nmeth.4463 28991892 PMC5937676

[bib2] Al-Sawaf, O., A. Fragoulis, C. Rosen, N. Keimes, E.A. Liehn, F. Hölzle, Y.W. Kan, T. Pufe, T.T. Sönmez, and C.J. Wruck. 2014. Nrf2 augments skeletal muscle regeneration after ischaemia-reperfusion injury. J. Pathol. 234:538–547. 10.1002/path.4418 25111334

[bib3] Alquicira-Hernandez, J., and J.E. Powell. 2020. Nebulosa recovers single cell gene expression signals by kernel density estimation. bioRxiv. (Preprint posted September 30, 2020) 10.1101/2020.09.29.315879 33459785

[bib4] Antebi, Y.E., J.M. Linton, H. Klumpe, B. Bintu, M. Gong, C. Su, R. McCardell, and M.B. Elowitz. 2017. Combinatorial Signal Perception in the BMP Pathway. Cell. 170:1184–1196.e24. 10.1016/j.cell.2017.08.015 28886385 PMC5612783

[bib5] Araki, H., Y. Tamada, S. Imoto, B. Dunmore, D. Sanders, S. Humphrey, M. Nagasaki, A. Doi, Y. Nakanishi, K. Yasuda, . 2009. Analysis of PPARalpha-dependent and PPARalpha-independent transcript regulation following fenofibrate treatment of human endothelial cells. Angiogenesis. 12:221–229. 10.1007/s10456-009-9142-8 19357976

[bib6] Aran, D., A.P. Looney, L. Liu, E. Wu, V. Fong, A. Hsu, S. Chak, R.P. Naikawadi, P.J. Wolters, A.R. Abate, . 2019. Reference-based analysis of lung single-cell sequencing reveals a transitional profibrotic macrophage. Nat. Immunol. 20:163–172. 10.1038/s41590-018-0276-y 30643263 PMC6340744

[bib7] Arnold, L., A. Henry, F. Poron, Y. Baba-Amer, N. van Rooijen, A. Plonquet, R.K. Gherardi, and B. Chazaud. 2007. Inflammatory monocytes recruited after skeletal muscle injury switch into antiinflammatory macrophages to support myogenesis. J. Exp. Med. 204:1057–1069. 10.1084/jem.20070075 17485518 PMC2118577

[bib8] Arnold, L., H. Perrin, C.B. de Chanville, M. Saclier, P. Hermand, L. Poupel, E. Guyon, F. Licata, W. Carpentier, J. Vilar, . 2015. CX3CR1 deficiency promotes muscle repair and regeneration by enhancing macrophage ApoE production. Nat. Commun. 6:8972. 10.1038/ncomms9972 26632270 PMC4686853

[bib9] Assadi, A., A. Zahabi, and R.A. Hart. 2020. GDF15, an update of the physiological and pathological roles it plays: a review. Pflugers Arch. 472:1535–1546. 10.1007/s00424-020-02459-1 32936319

[bib10] Baek, S.J., J.S. Kim, J.B. Nixon, R.P. DiAugustine, and T.E. Eling. 2004. Expression of NAG-1, a transforming growth factor-beta superfamily member, by troglitazone requires the early growth response gene EGR-1. J. Biol. Chem. 279:6883–6892. 10.1074/jbc.M305295200 14662774

[bib11] Baht, G.S., A. Bareja, D.E. Lee, R.R. Rao, R. Huang, J.L. Huebner, D.B. Bartlett, C.R. Hart, J.R. Gibson, I.R. Lanza, . 2020. Meteorin-like facilitates skeletal muscle repair through a Stat3/IGF-1 mechanism. Nat. Metab. 2:278–289. 10.1038/s42255-020-0184-y 32694780 PMC7504545

[bib12] Baitsch, D., H.H. Bock, T. Engel, R. Telgmann, C. Müller-Tidow, G. Varga, M. Bot, J. Herz, H. Robenek, A. von Eckardstein, and J.R. Nofer. 2011. Apolipoprotein E induces antiinflammatory phenotype in macrophages. Arterioscler. Thromb. Vasc. Biol. 31:1160–1168. 10.1161/ATVBAHA.111.222745 21350196 PMC3529398

[bib13] Becht, E., L. McInnes, J. Healy, C.A. Dutertre, I.W.H. Kwok, L.G. Ng, F. Ginhoux, and E.W. Newell. 2018. Dimensionality reduction for visualizing single-cell data using UMAP. Nat. Biotechnol. 10.1038/nbt.4314 30531897

[bib14] Bondesen, B.A., S.T. Mills, K.M. Kegley, and G.K. Pavlath. 2004. The COX-2 pathway is essential during early stages of skeletal muscle regeneration. Am. J. Physiol. Cell Physiol. 287:C475–C483. 10.1152/ajpcell.00088.2004 15084473

[bib15] Bootcov, M.R., A.R. Bauskin, S.M. Valenzuela, A.G. Moore, M. Bansal, X.Y. He, H.P. Zhang, M. Donnellan, S. Mahler, K. Pryor, . 1997. MIC-1, a novel macrophage inhibitory cytokine, is a divergent member of the TGF-beta superfamily. Proc. Natl. Acad. Sci. USA. 94:11514–11519. 10.1073/pnas.94.21.11514 9326641 PMC23523

[bib16] Borner, T., H.S. Wald, M.Y. Ghidewon, B. Zhang, Z. Wu, B.C. De Jonghe, D. Breen, and H.J. Grill. 2020. GDF15 Induces an Aversive Visceral Malaise State that Drives Anorexia and Weight Loss. Cell Rep. 31:107543. 10.1016/j.celrep.2020.107543 32320650 PMC7271892

[bib17] Breit, S.N., D.A. Brown, and V.W. Tsai. 2021. The GDF15-GFRAL Pathway in Health and Metabolic Disease: Friend or Foe? Annu. Rev. Physiol. 83:127–151. 10.1146/annurev-physiol-022020-045449 33228454

[bib18] Butler, A., P. Hoffman, P. Smibert, E. Papalexi, and R. Satija. 2018. Integrating single-cell transcriptomic data across different conditions, technologies, and species. Nat. Biotechnol. 36:411–420. 10.1038/nbt.4096 29608179 PMC6700744

[bib19] Capote, J., I. Kramerova, L. Martinez, S. Vetrone, E.R. Barton, H.L. Sweeney, M.C. Miceli, and M.J. Spencer. 2016. Osteopontin ablation ameliorates muscular dystrophy by shifting macrophages to a pro-regenerative phenotype. J. Cell Biol. 213:275–288. 10.1083/jcb.201510086 27091452 PMC5084275

[bib20] Chazaud, B. 2014. Macrophages: supportive cells for tissue repair and regeneration. Immunobiology. 219:172–178. 10.1016/j.imbio.2013.09.001 24080029

[bib21] Chazaud, B. 2020. Inflammation and Skeletal Muscle Regeneration: Leave It to the Macrophages! Trends Immunol. 41:481–492. 10.1016/j.it.2020.04.006 32362490

[bib22] Chen, E.Y., C.M. Tan, Y. Kou, Q. Duan, Z. Wang, G.V. Meirelles, N.R. Clark, and A. Ma’ayan. 2013. Enrichr: interactive and collaborative HTML5 gene list enrichment analysis tool. BMC Bioinformatics. 14:128. 10.1186/1471-2105-14-128 23586463 PMC3637064

[bib23] ENCODE Project Consortium. 2012. An integrated encyclopedia of DNA elements in the human genome. Nature. 489:57–74. 10.1038/nature11247 22955616 PMC3439153

[bib24] Corna, G., I. Caserta, A. Monno, P. Apostoli, A.A. Manfredi, C. Camaschella, and P. Rovere-Querini. 2016. The Repair of Skeletal Muscle Requires Iron Recycling through Macrophage Ferroportin. J. Immunol. 197:1914–1925. 10.4049/jimmunol.1501417 27465531

[bib25] Dadgar, S., Z. Wang, H. Johnston, A. Kesari, K. Nagaraju, Y.W. Chen, D.A. Hill, T.A. Partridge, M. Giri, R.J. Freishtat, . 2014. Asynchronous remodeling is a driver of failed regeneration in Duchenne muscular dystrophy. J. Cell Biol. 207:139–158. 10.1083/jcb.201402079 25313409 PMC4195829

[bib26] Daniel, B., B.L. Balint, Z.S. Nagy, and L. Nagy. 2014. Mapping the genomic binding sites of the activated retinoid X receptor in murine bone marrow-derived macrophages using chromatin immunoprecipitation sequencing. Methods Mol. Biol. 1204:15–24. 10.1007/978-1-4939-1346-6_2 25182757

[bib27] Daniel, B., G. Nagy, Z. Czimmerer, A. Horvath, D.W. Hammers, I. Cuaranta-Monroy, S. Poliska, P. Tzerpos, Z. Kolostyak, T.T. Hays, . 2018. The Nuclear Receptor PPARγ Controls Progressive Macrophage Polarization as a Ligand-Insensitive Epigenomic Ratchet of Transcriptional Memory. Immunity. 49:615–626.e6. 10.1016/j.immuni.2018.09.005 30332629 PMC6197058

[bib28] De Micheli, A.J., E.J. Laurilliard, C.L. Heinke, H. Ravichandran, P. Fraczek, S. Soueid-Baumgarten, I. De Vlaminck, O. Elemento, and B.D. Cosgrove. 2020. Single-Cell Analysis of the Muscle Stem Cell Hierarchy Identifies Heterotypic Communication Signals Involved in Skeletal Muscle Regeneration. Cell Rep. 30:3583–3595.e5. 10.1016/j.celrep.2020.02.067 32160558 PMC7091476

[bib29] Dell’Orso, S., A.H. Juan, K.D. Ko, F. Naz, J. Perovanovic, G. Gutierrez-Cruz, X. Feng, and V. Sartorelli. 2019. Single cell analysis of adult mouse skeletal muscle stem cells in homeostatic and regenerative conditions. Development. 146:dev181743. 10.1242/dev.181743 30890574 PMC6602351

[bib30] Denda-Nagai, K., S. Aida, K. Saba, K. Suzuki, S. Moriyama, S. Oo-Puthinan, M. Tsuiji, A. Morikawa, Y. Kumamoto, D. Sugiura, . 2010. Distribution and function of macrophage galactose-type C-type lectin 2 (MGL2/CD301b): efficient uptake and presentation of glycosylated antigens by dendritic cells. J. Biol. Chem. 285:19193–19204. 10.1074/jbc.M110.113613 20304916 PMC2885198

[bib31] Deng, B., M. Wehling-Henricks, S.A. Villalta, Y. Wang, and J.G. Tidball. 2012. IL-10 triggers changes in macrophage phenotype that promote muscle growth and regeneration. J. Immunol. 189:3669–3680. 10.4049/jimmunol.1103180 22933625 PMC3448810

[bib32] Dobin, A., C.A. Davis, F. Schlesinger, J. Drenkow, C. Zaleski, S. Jha, P. Batut, M. Chaisson, and T.R. Gingeras. 2013. STAR: ultrafast universal RNA-seq aligner. Bioinformatics. 29:15–21. 10.1093/bioinformatics/bts635 23104886 PMC3530905

[bib33] Duffield, J.S., S.J. Forbes, C.M. Constandinou, S. Clay, M. Partolina, S. Vuthoori, S. Wu, R. Lang, and J.P. Iredale. 2005. Selective depletion of macrophages reveals distinct, opposing roles during liver injury and repair. J. Clin. Invest. 115:56–65. 10.1172/JCI200522675 15630444 PMC539199

[bib34] Ewels, P.A., A. Peltzer, S. Fillinger, H. Patel, J. Alneberg, A. Wilm, M.U. Garcia, P. Di Tommaso, and S. Nahnsen. 2020. The nf-core framework for community-curated bioinformatics pipelines. Nat. Biotechnol. 38:276–278. 10.1038/s41587-020-0439-x 32055031

[bib35] Fadok, V.A., D.L. Bratton, A. Konowal, P.W. Freed, J.Y. Westcott, and P.M. Henson. 1998. Macrophages that have ingested apoptotic cells in vitro inhibit proinflammatory cytokine production through autocrine/paracrine mechanisms involving TGF-beta, PGE2, and PAF. J. Clin. Invest. 101:890–898. 10.1172/JCI1112 9466984 PMC508637

[bib36] Federico, A., and S. Monti. 2020. hypeR: an R package for geneset enrichment workflows. Bioinformatics. 36:1307–1308. 10.1093/bioinformatics/btz700 31498385 PMC7998712

[bib37] Feng, X., F. Naz, A.H. Juan, S. Dell’Orso, and V. Sartorelli. 2018. Identification of Skeletal Muscle Satellite Cells by Immunofluorescence with Pax7 and Laminin Antibodies. J. Vis. Exp. 134. 10.3791/57212 PMC6100681 29733324

[bib38] Geissmann, F., M.G. Manz, S. Jung, M.H. Sieweke, M. Merad, and K. Ley. 2010. Development of monocytes, macrophages, and dendritic cells. Science. 327:656–661. 10.1126/science.1178331 20133564 PMC2887389

[bib39] Giannakis, N., B.E. Sansbury, A. Patsalos, T.T. Hays, C.O. Riley, X. Han, M. Spite, and L. Nagy. 2019. Dynamic changes to lipid mediators support transitions among macrophage subtypes during muscle regeneration. Nat. Immunol. 20:626–636. 10.1038/s41590-019-0356-7 30936495 PMC6537107

[bib40] Gil, C.I., M. Ost, J. Kasch, S. Schumann, S. Heider, and S. Klaus. 2019. Role of GDF15 in active lifestyle induced metabolic adaptations and acute exercise response in mice. Sci. Rep. 9:20120. 10.1038/s41598-019-56922-w 31882966 PMC6934564

[bib41] Hardy, D., A. Besnard, M. Latil, G. Jouvion, D. Briand, C. Thépenier, Q. Pascal, A. Guguin, B. Gayraud-Morel, J.-M. Cavaillon, . 2016. Comparative Study of Injury Models for Studying Muscle Regeneration in Mice. PLoS One. 11:e0147198. 10.1371/journal.pone.0147198 26807982 PMC4726569

[bib42] Heinz, S., C. Benner, N. Spann, E. Bertolino, Y.C. Lin, P. Laslo, J.X. Cheng, C. Murre, H. Singh, and C.K. Glass. 2010. Simple combinations of lineage-determining transcription factors prime cis-regulatory elements required for macrophage and B cell identities. Mol. Cell. 38:576–589. 10.1016/j.molcel.2010.05.004 20513432 PMC2898526

[bib43] Heldin, C.H., and A. Moustakas. 2016. Signaling Receptors for TGF-β Family Members. Cold Spring Harb. Perspect. Biol. 8:a022053. 10.1101/cshperspect.a022053 27481709 PMC4968163

[bib44] Heredia, J.E., L. Mukundan, F.M. Chen, A.A. Mueller, R.C. Deo, R.M. Locksley, T.A. Rando, and A. Chawla. 2013. Type 2 innate signals stimulate fibro/adipogenic progenitors to facilitate muscle regeneration. Cell. 153:376–388. 10.1016/j.cell.2013.02.053 23582327 PMC3663598

[bib45] Hidestrand, M., S. Richards-Malcolm, C.M. Gurley, G. Nolen, B. Grimes, A. Waterstrat, G.V. Zant, and C.A. Peterson. 2008. Sca-1-expressing nonmyogenic cells contribute to fibrosis in aged skeletal muscle. J. Gerontol. A Biol. Sci. Med. Sci. 63:566–579. 10.1093/gerona/63.6.566 18559630 PMC2755567

[bib46] Hill, M., and G. Goldspink. 2003. Expression and splicing of the insulin-like growth factor gene in rodent muscle is associated with muscle satellite (stem) cell activation following local tissue damage. J. Physiol. 549:409–418. 10.1113/jphysiol.2002.035832 12692175 PMC2342958

[bib47] Hirano, T. 1998. Interleukin 6 and its receptor: ten years later. Int. Rev. Immunol. 16:249–284. 10.3109/08830189809042997 9505191

[bib48] Ho, A.T.V., A.R. Palla, M.R. Blake, N.D. Yucel, Y.X. Wang, K.E.G. Magnusson, C.A. Holbrook, P.E. Kraft, S.L. Delp, and H.M. Blau. 2017. Prostaglandin E2 is essential for efficacious skeletal muscle stem-cell function, augmenting regeneration and strength. Proc. Natl. Acad. Sci. USA. 114:6675–6684. 10.1073/pnas.1705420114 28607093 PMC5495271

[bib49] Ho, M.M., A. Manughian-Peter, W.R. Spivia, A. Taylor, and D.A. Fraser. 2016. Macrophage molecular signaling and inflammatory responses during ingestion of atherogenic lipoproteins are modulated by complement protein C1q. Atherosclerosis. 253:38–46. 10.1016/j.atherosclerosis.2016.08.019 27573737 PMC5064879

[bib50] Hofer, M., Z. Hoferová, J. Remšík, M. Nováková, J. Procházková, R. Fedr, J. Kohoutek, L. Dušek, A. Hampl, and K. Souček. 2018. Hematological findings in non-treated and gamma-irradiated mice deficient for MIC-1/GDF15. Physiol. Res. 67:623–636. 10.33549/physiolres.933810 29750874

[bib51] Houthuys, E., K. Movahedi, P. De Baetselier, J.A. Van Ginderachter, and P. Brouckaert. 2010. A method for the isolation and purification of mouse peripheral blood monocytes. J. Immunol. Methods. 359:1–10. 10.1016/j.jim.2010.04.004 20457160

[bib52] Hsiao, E.C., L.G. Koniaris, T. Zimmers-Koniaris, S.M. Sebald, T.V. Huynh, and S.J. Lee. 2000. Characterization of growth-differentiation factor 15, a transforming growth factor beta superfamily member induced following liver injury. Mol. Cell. Biol. 20:3742–3751. 10.1128/MCB.20.10.3742-3751.2000 10779363 PMC85678

[bib53] Iavarone, F., O. Guardiola, A. Scagliola, G. Andolfi, F. Esposito, A. Serrano, E. Perdiguero, S. Brunelli, P. Muñoz-Cánoves, and G. Minchiotti. 2020. Cripto shapes macrophage plasticity and restricts EndMT in injured and diseased skeletal muscle. EMBO Rep. 21:e49075. 10.15252/embr.201949075 32107853 PMC7132341

[bib54] Jin, R.M., J. Warunek, and E.A. Wohlfert. 2018. Chronic infection stunts macrophage heterogeneity and disrupts immune-mediated myogenesis. JCI Insight. 3:e121549. 10.1172/jci.insight.121549 PMC6237226 30232283

[bib55] Joe, A.W., L. Yi, A. Natarajan, F. Le Grand, L. So, J. Wang, M.A. Rudnicki, and F.M. Rossi. 2010. Muscle injury activates resident fibro/adipogenic progenitors that facilitate myogenesis. Nat. Cell Biol. 12:153–163. 10.1038/ncb2015 20081841 PMC4580288

[bib56] Johnen, H., S. Lin, T. Kuffner, D.A. Brown, V.W. Tsai, A.R. Bauskin, L. Wu, G. Pankhurst, L. Jiang, S. Junankar, . 2007. Tumor-induced anorexia and weight loss are mediated by the TGF-beta superfamily cytokine MIC-1. Nat. Med. 13:1333–1340. 10.1038/nm1677 17982462

[bib57] Juban, G. 2021. Transcriptional control of macrophage inflammatory shift during skeletal muscle regeneration. Semin. Cell Dev. Biol. 119:82–88. 10.1016/j.semcdb.2021.06.011 34183241

[bib58] Kempf, T., A. Zarbock, C. Widera, S. Butz, A. Stadtmann, J. Rossaint, M. Bolomini-Vittori, M. Korf-Klingebiel, L.C. Napp, B. Hansen, . 2011. GDF-15 is an inhibitor of leukocyte integrin activation required for survival after myocardial infarction in mice. Nat. Med. 17:581–588. 10.1038/nm.2354 21516086

[bib59] Kiss, M., Z. Czimmerer, G. Nagy, P. Bieniasz-Krzywiec, M. Ehling, A. Pap, S. Poliska, P. Boto, P. Tzerpos, A. Horvath, . 2017. Retinoid X receptor suppresses a metastasis-promoting transcriptional program in myeloid cells via a ligand-insensitive mechanism. Proc. Natl. Acad. Sci. USA. 114:10725–10730. 10.1073/pnas.1700785114 28923935 PMC5635866

[bib60] Kleinert, M., C. Clemmensen, K.A. Sjøberg, C.S. Carl, J.F. Jeppesen, J.F.P. Wojtaszewski, B. Kiens, and E.A. Richter. 2018. Exercise increases circulating GDF15 in humans. Mol. Metab. 9:187–191. 10.1016/j.molmet.2017.12.016 29398617 PMC5870087

[bib61] Kuleshov, M.V., M.R. Jones, A.D. Rouillard, N.F. Fernandez, Q. Duan, Z. Wang, S. Koplev, S.L. Jenkins, K.M. Jagodnik, A. Lachmann, . 2016. Enrichr: a comprehensive gene set enrichment analysis web server 2016 update. Nucleic Acids Res. 44(W1):W90-7. 10.1093/nar/gkw377 27141961 PMC4987924

[bib62] Laflamme, M.A., and C.E. Murry. 2011. Heart regeneration. Nature. 473:326–335. 10.1038/nature10147 21593865 PMC4091722

[bib63] Laurens, C., A. Parmar, E. Murphy, D. Carper, B. Lair, P. Maes, J. Vion, N. Boulet, C. Fontaine, M. Marquès, . 2020. Growth and differentiation factor 15 is secreted by skeletal muscle during exercise and promotes lipolysis in humans. JCI Insight. 5:e131870. 10.1172/jci.insight.131870 PMC7213799 32106110

[bib64] Lavin, Y., D. Winter, R. Blecher-Gonen, E. David, H. Keren-Shaul, M. Merad, S. Jung, and I. Amit. 2014. Tissue-resident macrophage enhancer landscapes are shaped by the local microenvironment. Cell. 159:1312–1326. 10.1016/j.cell.2014.11.018 25480296 PMC4437213

[bib65] Lawton, L.N., M.F. Bonaldo, P.C. Jelenc, L. Qiu, S.A. Baumes, R.A. Marcelino, G.M. de Jesus, S. Wellington, J.A. Knowles, D. Warburton, . 1997. Identification of a novel member of the TGF-beta superfamily highly expressed in human placenta. Gene. 203:17–26. 10.1016/S0378-1119(97)00485-X 9426002

[bib66] Lemos, D.R., F. Babaeijandaghi, M. Low, C.K. Chang, S.T. Lee, D. Fiore, R.H. Zhang, A. Natarajan, S.A. Nedospasov, and F.M. Rossi. 2015. Nilotinib reduces muscle fibrosis in chronic muscle injury by promoting TNF-mediated apoptosis of fibro/adipogenic progenitors. Nat. Med. 21:786–794. 10.1038/nm.3869 26053624

[bib67] Li, H., and R. Durbin. 2009. Fast and accurate short read alignment with Burrows-Wheeler transform. Bioinformatics. 25:1754–1760. 10.1093/bioinformatics/btp324 19451168 PMC2705234

[bib68] Li, H., B. Handsaker, A. Wysoker, T. Fennell, J. Ruan, N. Homer, G. Marth, G. Abecasis, and R. Durbin. 1000 Genome Project Data Processing Subgroup. 2009. The Sequence Alignment/Map format and SAMtools. Bioinformatics. 25:2078–2079. 10.1093/bioinformatics/btp352 19505943 PMC2723002

[bib69] Liberzon, A., C. Birger, H. Thorvaldsdóttir, M. Ghandi, J.P. Mesirov, and P. Tamayo. 2015. The Molecular Signatures Database (MSigDB) hallmark gene set collection. Cell Syst. 1:417–425. 10.1016/j.cels.2015.12.004 26771021 PMC4707969

[bib70] Liberzon, A., A. Subramanian, R. Pinchback, H. Thorvaldsdóttir, P. Tamayo, and J.P. Mesirov. 2011. Molecular signatures database (MSigDB) 3.0. Bioinformatics. 27:1739–1740. 10.1093/bioinformatics/btr260 21546393 PMC3106198

[bib71] Love, M.I., W. Huber, and S. Anders. 2014. Moderated estimation of fold change and dispersion for RNA-seq data with DESeq2. Genome Biol. 15:550. 10.1186/s13059-014-0550-8 25516281 PMC4302049

[bib72] Lu, H., D. Huang, N. Saederup, I.F. Charo, R.M. Ransohoff, and L. Zhou. 2011. Macrophages recruited via CCR2 produce insulin-like growth factor-1 to repair acute skeletal muscle injury. FASEB J. 25:358–369. 10.1096/fj.10-171579 20889618 PMC3005436

[bib73] McKellar, D.W., L.D. Walter, L.T. Song, M. Mantri, M.F.Z. Wang, I. De Vlaminck, and B.D. Cosgrove. 2020. Strength in numbers: Large-scale integration of single-cell transcriptomic data reveals rare, transient muscle progenitor cell states in muscle regeneration. bioRxiv. (Preprint posted December 23, 2020) 10.1101/2020.12.01.407460

[bib74] Mounier, R., M. Théret, L. Arnold, S. Cuvellier, L. Bultot, O. Göransson, N. Sanz, A. Ferry, K. Sakamoto, M. Foretz, . 2013. AMPKα1 regulates macrophage skewing at the time of resolution of inflammation during skeletal muscle regeneration. Cell Metab. 18:251–264. 10.1016/j.cmet.2013.06.017 23931756

[bib75] Mourkioti, F., and N. Rosenthal. 2005. IGF-1, inflammation and stem cells: interactions during muscle regeneration. Trends Immunol. 26:535–542. 10.1016/j.it.2005.08.002 16109502

[bib76] Mullican, S.E., X. Lin-Schmidt, C.N. Chin, J.A. Chavez, J.L. Furman, A.A. Armstrong, S.C. Beck, V.J. South, T.Q. Dinh, T.D. Cash-Mason, . 2017. GFRAL is the receptor for GDF15 and the ligand promotes weight loss in mice and nonhuman primates. Nat. Med. 23:1150–1157. 10.1038/nm.4392 28846097

[bib77] Muñoz-Cánoves, P., C. Scheele, B.K. Pedersen, and A.L. Serrano. 2013. Interleukin-6 myokine signaling in skeletal muscle: a double-edged sword? FEBS J. 280:4131–4148. 10.1111/febs.12338 23663276 PMC4163639

[bib78] Nagy, G., and L. Nagy. 2020. Motif grammar: The basis of the language of gene expression. Comput. Struct. Biotechnol. J. 18:2026–2032. 10.1016/j.csbj.2020.07.007 32802274 PMC7406977

[bib79] Novak, M.L., and T.J. Koh. 2013. Macrophage phenotypes during tissue repair. J. Leukoc. Biol. 93:875–881. 10.1189/jlb.1012512 23505314 PMC3656331

[bib80] Odegaard, J.I., and A. Chawla. 2011. Alternative macrophage activation and metabolism. Annu. Rev. Pathol. 6:275–297. 10.1146/annurev-pathol-011110-130138 21034223 PMC3381938

[bib81] Okabe, Y., and R. Medzhitov. 2014. Tissue-specific signals control reversible program of localization and functional polarization of macrophages. Cell. 157:832–844. 10.1016/j.cell.2014.04.016 24792964 PMC4137874

[bib82] Oprescu, S.N., F. Yue, J. Qiu, L.F. Brito, and S. Kuang. 2020. Temporal Dynamics and Heterogeneity of Cell Populations during Skeletal Muscle Regeneration. iScience. 23:100993. 10.1016/j.isci.2020.100993 32248062 PMC7125354

[bib83] Palani, S., M. Maksimow, M. Miiluniemi, K. Auvinen, S. Jalkanen, and M. Salmi. 2011. Stabilin-1/CLEVER-1, a type 2 macrophage marker, is an adhesion and scavenging molecule on human placental macrophages. Eur. J. Immunol. 41:2052–2063. 10.1002/eji.201041376 21480214

[bib84] Panduro, M., C. Benoist, and D. Mathis. 2018. T_reg_ cells limit IFN-γ production to control macrophage accrual and phenotype during skeletal muscle regeneration. Proc. Natl. Acad. Sci. USA. 115:E2585–E2593. 10.1073/pnas.1800618115 29476012 PMC5856564

[bib85] Patel, S., A. Alvarez-Guaita, A. Melvin, D. Rimmington, A. Dattilo, E.L. Miedzybrodzka, I. Cimino, A.C. Maurin, G.P. Roberts, C.L. Meek, . 2019. GDF15 Provides an Endocrine Signal of Nutritional Stress in Mice and Humans. Cell Metab. 29:707–718.e8. 10.1016/j.cmet.2018.12.016 30639358 PMC6408327

[bib86] Patro, R., G. Duggal, M.I. Love, R.A. Irizarry, and C. Kingsford. 2017. Salmon provides fast and bias-aware quantification of transcript expression. Nat. Methods. 14:417–419. 10.1038/nmeth.4197 28263959 PMC5600148

[bib87] Patsalos, A., A. Pap, T. Varga, G. Trencsenyi, G.A. Contreras, I. Garai, Z. Papp, B. Dezso, E. Pintye, and L. Nagy. 2017. In situ macrophage phenotypic transition is affected by altered cellular composition prior to acute sterile muscle injury. J. Physiol. 595:5815–5842. 10.1113/JP274361 28714082 PMC5577539

[bib88] Patsalos, A., Z. Simandi, T.T. Hays, M. Peloquin, M. Hajian, I. Restrepo, P.M. Coen, A.J. Russell, and L. Nagy. 2018. In vivo GDF3 administration abrogates aging related muscle regeneration delay following acute sterile injury. Aging Cell. 17:e12815. 10.1111/acel.12815 30003692 PMC6156497

[bib89] Patsalos, A., P. Tzerpos, L. Halasz, G. Nagy, A. Pap, N. Giannakis, K. Lyroni, V. Koliaraki, E. Pintye, B. Dezso, . 2019. The BACH1-HMOX1 Regulatory Axis Is Indispensable for Proper Macrophage Subtype Specification and Skeletal Muscle Regeneration. J. Immunol. 203:1532–1547. 10.4049/jimmunol.1900553 31405954 PMC6736746

[bib90] Patsalos, A., P. Tzerpos, X. Wei, and L. Nagy. 2021. Myeloid cell diversification during regenerative inflammation: Lessons from skeletal muscle. Semin. Cell Dev. Biol. 119:89–100. 10.1016/j.semcdb.2021.05.005 34016524 PMC8530826

[bib91] Patterson-Cross, R.B., A.J. Levine, and V. Menon. 2021. Selecting single cell clustering parameter values using subsampling-based robustness metrics. BMC Bioinformatics. 22:39. 10.1186/s12859-021-03957-4 33522897 PMC7852188

[bib92] Perdiguero, E., P. Sousa-Victor, V. Ruiz-Bonilla, M. Jardí, C. Caelles, A.L. Serrano, and P. Muñoz-Cánoves. 2011. p38/MKP-1-regulated AKT coordinates macrophage transitions and resolution of inflammation during tissue repair. J. Cell Biol. 195:307–322. 10.1083/jcb.201104053 21987635 PMC3198158

[bib93] Ramachandran, P., A. Pellicoro, M.A. Vernon, L. Boulter, R.L. Aucott, A. Ali, S.N. Hartland, V.K. Snowdon, A. Cappon, T.T. Gordon-Walker, . 2012. Differential Ly-6C expression identifies the recruited macrophage phenotype, which orchestrates the regression of murine liver fibrosis. Proc. Natl. Acad. Sci. USA. 109:E3186–E3195. 10.1073/pnas.1119964109 23100531 PMC3503234

[bib94] Ramírez, F., D.P. Ryan, B. Grüning, V. Bhardwaj, F. Kilpert, A.S. Richter, S. Heyne, F. Dündar, and T. Manke. 2016. deepTools2: a next generation web server for deep-sequencing data analysis. Nucleic Acids Res. 44(W1):W160-5. 10.1093/nar/gkw257 27079975 PMC4987876

[bib95] Rantakari, P., D.A. Patten, J. Valtonen, M. Karikoski, H. Gerke, H. Dawes, J. Laurila, S. Ohlmeier, K. Elima, S.G. Hübscher, . 2016. Stabilin-1 expression defines a subset of macrophages that mediate tissue homeostasis and prevent fibrosis in chronic liver injury. Proc. Natl. Acad. Sci. USA. 113:9298–9303. 10.1073/pnas.1604780113 27474165 PMC4995933

[bib96] Rapalino, O., O. Lazarov-Spiegler, E. Agranov, G.J. Velan, E. Yoles, M. Fraidakis, A. Solomon, R. Gepstein, A. Katz, M. Belkin, . 1998. Implantation of stimulated homologous macrophages results in partial recovery of paraplegic rats. Nat. Med. 4:814–821. 10.1038/nm0798-814 9662373

[bib97] Rivollier, A., J. He, A. Kole, V. Valatas, and B.L. Kelsall. 2012. Inflammation switches the differentiation program of Ly6Chi monocytes from antiinflammatory macrophages to inflammatory dendritic cells in the colon. J. Exp. Med. 209:139–155. 10.1084/jem.20101387 22231304 PMC3260867

[bib98] Robinson, M.D., D.J. McCarthy, and G.K. Smyth. 2010. edgeR: a Bioconductor package for differential expression analysis of digital gene expression data. Bioinformatics. 26:139–140. 10.1093/bioinformatics/btp616 19910308 PMC2796818

[bib99] Ruffell, D., F. Mourkioti, A. Gambardella, P. Kirstetter, R.G. Lopez, N. Rosenthal, and C. Nerlov. 2009. A CREB-C/EBPbeta cascade induces M2 macrophage-specific gene expression and promotes muscle injury repair. Proc. Natl. Acad. Sci. USA. 106:17475–17480. 10.1073/pnas.0908641106 19805133 PMC2762675

[bib100] Saclier, M., M. Lapi, C. Bonfanti, G. Rossi, S. Antonini, and G. Messina. 2020. The Transcription Factor Nfix Requires RhoA-ROCK1 Dependent Phagocytosis to Mediate Macrophage Skewing during Skeletal Muscle Regeneration. Cells. 9:E708. 10.3390/cells9030708 32183151 PMC7140652

[bib101] Saclier, M., H. Yacoub-Youssef, A.L. Mackey, L. Arnold, H. Ardjoune, M. Magnan, F. Sailhan, J. Chelly, G.K. Pavlath, R. Mounier, . 2013. Differentially activated macrophages orchestrate myogenic precursor cell fate during human skeletal muscle regeneration. Stem Cells. 31:384–396. 10.1002/stem.1288 23169615

[bib102] Serrano, A.L., B. Baeza-Raja, E. Perdiguero, M. Jardí, and P. Muñoz-Cánoves. 2008. Interleukin-6 is an essential regulator of satellite cell-mediated skeletal muscle hypertrophy. Cell Metab. 7:33–44. 10.1016/j.cmet.2007.11.011 18177723

[bib103] Silva, W.N., P.H.D.M. Prazeres, A.E. Paiva, L. Lousado, A.O.M. Turquetti, R.S.N. Barreto, E.C. de Alvarenga, M.A. Miglino, R. Gonçalves, A. Mintz, and A. Birbrair. 2018. Macrophage-derived GPNMB accelerates skin healing. Exp. Dermatol. 27:630–635. 10.1111/exd.13524 29505115 PMC6013359

[bib104] Stables, M.J., S. Shah, E.B. Camon, R.C. Lovering, J. Newson, J. Bystrom, S. Farrow, and D.W. Gilroy. 2011. Transcriptomic analyses of murine resolution-phase macrophages. Blood. 118:e192–e208. 10.1182/blood-2011-04-345330 22012065 PMC5362087

[bib105] Strassmann, G., M. Fong, J.S. Kenney, and C.O. Jacob. 1992. Evidence for the involvement of interleukin 6 in experimental cancer cachexia. J. Clin. Invest. 89:1681–1684. 10.1172/JCI115767 1569207 PMC443047

[bib106] Stuard, W.L., R. Titone, and D.M. Robertson. 2020. The IGF/Insulin-IGFBP Axis in Corneal Development, Wound Healing, and Disease. Front. Endocrinol. (Lausanne). 11:24. 10.3389/fendo.2020.00024 32194500 PMC7062709

[bib107] Subramanian, A., P. Tamayo, V.K. Mootha, S. Mukherjee, B.L. Ebert, M.A. Gillette, A. Paulovich, S.L. Pomeroy, T.R. Golub, E.S. Lander, and J.P. Mesirov. 2005. Gene set enrichment analysis: a knowledge-based approach for interpreting genome-wide expression profiles. Proc. Natl. Acad. Sci. USA. 102:15545–15550. 10.1073/pnas.0506580102 16199517 PMC1239896

[bib108] Suzuki, T., S. Hayashi, Y. Miki, Y. Nakamura, T. Moriya, A. Sugawara, T. Ishida, N. Ohuchi, and H. Sasano. 2006. Peroxisome proliferator-activated receptor gamma in human breast carcinoma: a modulator of estrogenic actions. Endocr. Relat. Cancer. 13:233–250. 10.1677/erc.1.01075 16601291

[bib109] Szanto, A., B.L. Balint, Z.S. Nagy, E. Barta, B. Dezso, A. Pap, L. Szeles, S. Poliska, M. Oros, R.M. Evans, . 2010. STAT6 transcription factor is a facilitator of the nuclear receptor PPARγ-regulated gene expression in macrophages and dendritic cells. Immunity. 33:699–712. 10.1016/j.immuni.2010.11.009 21093321 PMC3052437

[bib110] Thorvaldsdóttir, H., J.T. Robinson, and J.P. Mesirov. 2013. Integrative Genomics Viewer (IGV): high-performance genomics data visualization and exploration. Brief. Bioinform. 14:178–192. 10.1093/bib/bbs017 22517427 PMC3603213

[bib111] Tidball, J.G. 2017. Regulation of muscle growth and regeneration by the immune system. Nat. Rev. Immunol. 17:165–178. 10.1038/nri.2016.150 28163303 PMC5452982

[bib112] Tidball, J.G., and S.A. Villalta. 2010. Regulatory interactions between muscle and the immune system during muscle regeneration. Am. J. Physiol. Regul. Integr. Comp. Physiol. 298:R1173–R1187. 10.1152/ajpregu.00735.2009 20219869 PMC2867520

[bib113] Tonkin, J., L. Temmerman, R.D. Sampson, E. Gallego-Colon, L. Barberi, D. Bilbao, M.D. Schneider, A. Musarò, and N. Rosenthal. 2015. Monocyte/Macrophage-derived IGF-1 Orchestrates Murine Skeletal Muscle Regeneration and Modulates Autocrine Polarization. Mol. Ther. 23:1189–1200. 10.1038/mt.2015.66 25896247 PMC4817788

[bib114] Tsai, V.W.W., Y. Husaini, A. Sainsbury, D.A. Brown, and S.N. Breit. 2018. The MIC-1/GDF15-GFRAL Pathway in Energy Homeostasis: Implications for Obesity, Cachexia, and Other Associated Diseases. Cell Metab. 28:353–368. 10.1016/j.cmet.2018.07.018 30184485

[bib115] Uaesoontrachoon, K., D.K. Wasgewatte Wijesinghe, E.J. Mackie, and C.N. Pagel. 2013. Osteopontin deficiency delays inflammatory infiltration and the onset of muscle regeneration in a mouse model of muscle injury. Dis. Model. Mech. 6:197–205. 22917925 10.1242/dmm.009993 PMC3529351

[bib116] Uezumi, A., S. Fukada, N. Yamamoto, S. Takeda, and K. Tsuchida. 2010. Mesenchymal progenitors distinct from satellite cells contribute to ectopic fat cell formation in skeletal muscle. Nat. Cell Biol. 12:143–152. 10.1038/ncb2014 20081842

[bib117] Van de Sande, B., C. Flerin, K. Davie, M. De Waegeneer, G. Hulselmans, S. Aibar, R. Seurinck, W. Saelens, R. Cannoodt, Q. Rouchon, . 2020. A scalable SCENIC workflow for single-cell gene regulatory network analysis. Nat. Protoc. 15:2247–2276. 10.1038/s41596-020-0336-2 32561888

[bib118] Varga, T., R. Mounier, P. Gogolak, S. Poliska, B. Chazaud, and L. Nagy. 2013. Tissue LyC6- macrophages are generated in the absence of circulating LyC6- monocytes and Nur77 in a model of muscle regeneration. J. Immunol. 191:5695–5701. 10.4049/jimmunol.1301445 24133167

[bib119] Varga, T., R. Mounier, A. Patsalos, P. Gogolák, M. Peloquin, A. Horvath, A. Pap, B. Daniel, G. Nagy, E. Pintye, . 2016 a. Macrophage PPARγ, a Lipid Activated Transcription Factor Controls the Growth Factor GDF3 and Skeletal Muscle Regeneration. Immunity. 45:1038–1051. 10.1016/j.immuni.2016.10.016 27836432 PMC5142832

[bib120] Varga, T., R. Mounier, A. Horvath, S. Cuvellier, F. Dumont, S. Poliska, H. Ardjoune, G. Juban, L. Nagy, and B. Chazaud. 2016 b. Highly Dynamic Transcriptional Signature of Distinct Macrophage Subsets during Sterile Inflammation, Resolution, and Tissue Repair. J. Immunol. 196:4771–4782. 10.4049/jimmunol.1502490 27183604

[bib121] Vats, D., L. Mukundan, J.I. Odegaard, L. Zhang, K.L. Smith, C.R. Morel, R.A. Wagner, D.R. Greaves, P.J. Murray, and A. Chawla. 2006. Oxidative metabolism and PGC-1β attenuate macrophage-mediated inflammation. Cell Metab. 4:13–24. 10.1016/j.cmet.2006.05.011 16814729 PMC1904486

[bib122] von Maltzahn, J., A.E. Jones, R.J. Parks, and M.A. Rudnicki. 2013. Pax7 is critical for the normal function of satellite cells in adult skeletal muscle. Proc. Natl. Acad. Sci. USA. 110:16474–16479. 10.1073/pnas.1307680110 24065826 PMC3799311

[bib123] Wang, H., D.W. Melton, L. Porter, Z.U. Sarwar, L.M. McManus, and P.K. Shireman. 2014. Altered macrophage phenotype transition impairs skeletal muscle regeneration. Am. J. Pathol. 184:1167–1184. 10.1016/j.ajpath.2013.12.020 24525152 PMC3969996

[bib124] Welc, S.S., M. Wehling-Henricks, J. Antoun, T.T. Ha, I. Tous, and J.G. Tidball. 2020. Differential Effects of Myeloid Cell PPARδ and IL-10 in Regulating Macrophage Recruitment, Phenotype, and Regeneration following Acute Muscle Injury. J. Immunol. 205:1664–1677. 10.4049/jimmunol.2000247 32817369 PMC7484367

[bib125] Welch, J.S., M. Ricote, T.E. Akiyama, F.J. Gonzalez, and C.K. Glass. 2003. PPARgamma and PPARdelta negatively regulate specific subsets of lipopolysaccharide and IFN-gamma target genes in macrophages. Proc. Natl. Acad. Sci. USA. 100:6712–6717. 10.1073/pnas.1031789100 12740443 PMC164512

[bib126] Yang, L., C.C. Chang, Z. Sun, D. Madsen, H. Zhu, S.B. Padkjær, X. Wu, T. Huang, K. Hultman, S.J. Paulsen, . 2017. GFRAL is the receptor for GDF15 and is required for the anti-obesity effects of the ligand. Nat. Med. 23:1158–1166. 10.1038/nm.4394 28846099

[bib127] Yona, S., K.W. Kim, Y. Wolf, A. Mildner, D. Varol, M. Breker, D. Strauss-Ayali, S. Viukov, M. Guilliams, A. Misharin, . 2013. Fate mapping reveals origins and dynamics of monocytes and tissue macrophages under homeostasis. Immunity. 38:79–91. 10.1016/j.immuni.2012.12.001 23273845 PMC3908543

[bib128] Yu, J., B. Shen, E.S. Chu, N. Teoh, K.F. Cheung, C.W. Wu, S. Wang, C.N. Lam, H. Feng, J. Zhao, . 2010. Inhibitory role of peroxisome proliferator-activated receptor gamma in hepatocarcinogenesis in mice and in vitro. Hepatology. 51:2008–2019. 10.1002/hep.23550 20512989

[bib129] Zhang, L., J. Du, Z. Hu, G. Han, P. Delafontaine, G. Garcia, and W.E. Mitch. 2009. IL-6 and serum amyloid A synergy mediates angiotensin II-induced muscle wasting. J. Am. Soc. Nephrol. 20:604–612. 10.1681/ASN.2008060628 19158350 PMC2653674

[bib130] Zhang, Y., T. Liu, C.A. Meyer, J. Eeckhoute, D.S. Johnson, B.E. Bernstein, C. Nusbaum, R.M. Myers, M. Brown, W. Li, and X.S. Liu. 2008. Model-based analysis of ChIP-Seq (MACS). Genome Biol. 9:R137. 10.1186/gb-2008-9-9-r137 18798982 PMC2592715

[bib131] Zhang, Y., L.A. Moszczynski, Q. Liu, J. Jiang, D. Zhao, D. Quan, T. Mele, V. McAlister, A. Jevnikar, S.J. Baek, . 2017. Over-expression of growth differentiation factor 15 (GDF15) preventing cold ischemia reperfusion (I/R) injury in heart transplantation through Foxo3a signaling. Oncotarget. 8:36531–36544. 10.18632/oncotarget.16607 28388574 PMC5482674

[bib132] Zigmond, E., C. Varol, J. Farache, E. Elmaliah, A.T. Satpathy, G. Friedlander, M. Mack, N. Shpigel, I.G. Boneca, K.M. Murphy, . 2012. Ly6C hi monocytes in the inflamed colon give rise to proinflammatory effector cells and migratory antigen-presenting cells. Immunity. 37:1076–1090. 10.1016/j.immuni.2012.08.026 23219392

